# Noradrenergic and cholinergic innervation of the normal human heart and changes associated with cardiomyopathy

**DOI:** 10.1002/ar.25686

**Published:** 2025-05-14

**Authors:** Peter Hanna, Donald B. Hoover, Logan G. Kirkland, Elizabeth H. Smith, Megan D. Poston, Stanley G. Peirce, Chloe G. Garbe, Tasha K. Phillips, Steven Cha, Shumpei Mori, Jaclyn A. Brennan, John Andrew Armour, Eric Rytkin, Igor R. Efimov, Olujimi A. Ajijola, Jeffrey L. Ardell, Kalyanam Shivkumar

**Affiliations:** ^1^ David Geffen School of Medicine University of California Los Angeles (UCLA) Los Angeles California USA; ^2^ Molecular, Cellular, and Integrative Physiology Program University of California Los Angeles (UCLA) Los Angeles California USA; ^3^ Department of Biomedical Sciences, Quillen College of Medicine East Tennessee State University Johnson City Tennessee USA; ^4^ Center of Excellence in Inflammation, Infectious Disease and Immunity East Tennessee State University Johnson City Tennessee USA; ^5^ Department of Biomedical Engineering George Washington University Washington, DC USA; ^6^ Department of Biomedical Engineering Northwestern University Chicago Illinois USA; ^7^ Department of Medicine (Cardiology) Northwestern University Chicago Illinois USA

**Keywords:** cardiac innervation, cardiomyopathy, cholinergic, immunohistochemistry, intrinsic cardiac nervous system, neuronal remodeling, noradrenergic

## Abstract

Autonomic nerves are crucial in cardiac function and pathology. However, data on the distribution of cholinergic and noradrenergic nerves in normal and pathologic human hearts is lacking. Nonfailing donor hearts were pressure‐perfusion fixed, imaged, and dissected. Left ventricular cardiomyopathy samples were also obtained. Fixed frozen sections were immunostained for nerves, and adjacent tissue underwent clearing for 3D visualization. Cholinergic and noradrenergic nerves were evenly abundant in both atria, except the sinoatrial node, where vesicular acetylcholine transporter (VAChT) nerves were dominant. Noradrenergic consistently outnumbered cholinergic nerves in right (RV) and left ventricular (LV) regions. Noradrenergic innervation of LV regions varied between donors. Cholinergic innervation was higher in RV compared to LV samples, which generally had reduced VAChT nerves. Marked neural remodeling occurred in three cardiomyopathy cases. Tyrosine hydroxylase (TH) nerve density was increased in the right atrial appendage, and all nerves showed a trend to decrease in the left atrial appendage. Cholinergic innervation was reduced in the LV, and TH innervation was heterogeneous. Noradrenergic nerves were present in granulation tissue but absent in regions of dense scar. Some border zone regions had reduced TH innervation but no hyperinnervation. Dual innervation of most atrial regions supports balanced regulation of atrial function. Higher cholinergic input to the sinoatrial node favors vagal dominance in heart rate regulation. Innervation patterns support a significant role of noradrenergic input to the ventricle, especially on the left. Both atrial and ventricular nerves remodel in cardiomyopathy, providing a foundation for asymmetric neural input and dysregulation of cardiac electromechanical function.

**Abbreviations**
AChE: acetylcholinesterase
ACh: acetylcholine
ABC: avidin biotin complex
BSA: bovine serum albumin
ChAT: choline acetyltransferase
CM: cardiomyopathy
Connexin 43: Cx43
EF: ejection fraction
ICM: ischemic cardiomyopathy
iDISCO+: modified immunolabeling‐enabled three‐Dimensional Imaging of Solvent‐Cleared Organs
LA: left atrial
LV: left ventricular
MI: myocardial infarction
NET: norepinephrine transporter
NICM: non‐ischemic cardiomyopathy
PGP9.5: protein gene product 9.5
RA: right atrial
RV: right ventricular
SAN: sinoatrial node
TH: tyrosine hydroxylase
UCHL1: ubiquitin carboxyl‐terminal hydrolase L1
VAChT: vesicular acetylcholine transporter

## 1 INTRODUCTION

The autonomic nervous system has a crucial role in regulating all aspects of cardiac function, and this is in part accomplished by dual innervation by noradrenergic sympathetic and cholinergic parasympathetic nerve fibers (Habecker et al., 2016). These intracardiac neural networks have their origin in autonomic ganglia whose localization and function have been described for many species ranging in size from mice to humans (Hoard et al., 2008; Horackova et al., 1999; Pauza et al., 2000; Pauziene et al., 2017; Rysevaite et al., 2011; Saburkina et al., 2014; Saburkina et al., 2023; Yuan et al., 1994). Cardiac‐projecting sympathetic neurons are in the stellate and neighboring paravertebral ganglia, while cardiac postganglionic parasympathetic neurons are in numerous, interconnected ganglia distributed at subepicardial sites across the atria and basal aspects of the ventricles, near the level of the atrioventricular valves (Pauza et al., 2000). Collectively, these cardiac ganglionated plexuses and their projections within the heart are known as the intrinsic cardiac nervous system (Armour, 2008; Armour et al., 1997). Neurons located in sympathetic and intrinsic cardiac ganglia are not passive relay stations but integrate various inputs to regulate the activity of cardiac‐projecting nerve fibers (Ardell & Armour, 2016). The presence of postganglionic noradrenergic and cholinergic nerve fibers within the heart is a critical factor in maintaining normal, balanced regulation of cardiac function and responding appropriately to stressors. Despite this, little effort has been directed at elucidating regional innervation patterns in this anatomically complex organ in humans.

Various histological techniques have been used for selective visualization of noradrenergic and cholinergic nerve fibers. Early work identified noradrenergic neurons and their projections by using a histofluorescence technique that showed the localization of endogenous catecholamines (Jacobowitz, 1967; Laties et al., 1967). This approach was ultimately replaced by immunohistochemical methods using antibodies to specific noradrenergic marker proteins such as tyrosine hydroxylase (TH). Identification of cholinergic nerves has been more difficult since there is no method to visualize endogenous acetylcholine (ACh). Enzyme histochemistry for acetylcholinesterase, which hydrolyzes ACh to terminate cholinergic transmission, had been used extensively for labeling cholinergic nerve fibers (Jacobowitz, 1967; Kent et al., 1974). However, this marker is not entirely selective since cholinesterases are also expressed by some non‐cholinergic neurons. Depending on location, these AChE‐positive nerves contained predominantly cholinergic or noradrenergic nerve fibers or a mixture of both. More recent work has utilized immunohistochemistry to target proteins in the ACh synthesis and release process, and these markers are selective for cholinergic neurons. Specifically, this work has used antibodies directed at choline acetyltransferase (ChAT) (Yasuhara et al., 2007), the high affinity choline transporter (Hoover et al., 2004), and was recently used to identify all nerves traversing the hilum of human heart (Petraitiene et al., 2014). Antibodies against vesicular ACh transporter (VAChT) (Aras et al., 2022; Fregoso & Hoover, 2012; Hanna et al., 2021; Hoard et al., 2008) have also been used to expand our understanding of cholinergic innervation of the heart.

The most extensive mapping of noradrenergic and cholinergic nerves in the heart has been accomplished in small animal models, where the size maximizes content within tissue sections and enables use of sophisticated techniques such as imaging of immunostained whole mounts and cleared hearts to reveal the 3D, in situ distribution of nerves and nerve fibers (Bizanti et al., 2023; Rajendran et al., 2019; Zhang et al., 2023). This work has mapped an extensive network of noradrenergic and cholinergic nerve fibers throughout the atria (including the nodes), shown abundant transmural noradrenergic innervation throughout the ventricles, and established the presence of some cholinergic input to the ventricles (Dajani et al., 2023). While fewer studies have evaluated parasympathetic and sympathetic innervation of the human hearts, these studies have established a conserved nature of the intrinsic cardiac nervous system by (1) mapping the intrinsic cardiac ganglia and their connectivity (Armour et al., 1997; Pauza et al., 2000), and (2) identifying TH+ and AChE+ nerve fibers in tissue sections from a limited number of atrial and ventricular regions, including nodes and the proximal conducting system (Crick et al., 1994; Kawano et al., 2003; Kent et al., 1974; Wharton et al., 1990). Furthermore, the neurochemical anatomy of human intrinsic cardiac ganglia has been examined, providing evidence for an integrative function (Hoover et al., 2009; Singh et al., 1999; Singh et al., 2009).

There is also evidence that remodeling of sympathetic innervation occurs in the ventricles of humans with cardiac disease. While some supporting evidence comes from immunohistochemical evaluation (Cao, Fishbein, et al., 2000; Oki et al., 1994), most studies of noradrenergic remodeling in human cardiac disease have relied on low resolution in vivo imaging methodology, which suggests heterogeneous or widespread sympathetic nerve fiber loss in the ventricles (Fallavollita et al., 2014; Thackeray & Bengel, 2013). The impact of cardiac disease on cholinergic innervation in humans has received little attention and has primarily focused on the atrium (Li et al., 2013; Nguyen et al., 2009).

Our primary goals for this project were to (1) map and quantify cholinergic and noradrenergic innervation of the heart by immunohistochemical analysis of tissue sections obtained from over 30 regions spanning the bilateral atria and bilateral basal to apical ventricular walls and (2) identify patterns of neuronal remodeling that occur in cases of severe cardiomyopathy (CM). Cholinergic nerve fibers were labeled using an antibody to the specific marker, VAChT, and noradrenergic fibers were labeled using TH antibodies. Nerve bundles and fibers in separate sections were labeled with an antibody to the pan‐neuronal marker, protein gene product 9.5 (PGP9.5), which is also known as ubiquitin carboxyl‐terminal hydrolase L1 (UCHL1), an important regulator of protein ubiquitination.

## 2 EXPERIMENTAL PROCEDURES

### 2.1 Human heart specimens

This study used deidentified tissue samples collected from donor hearts in protocols deemed IRB exempt by the IRBs at the University of California, Los Angeles, George Washington University, and Northwestern University. Nine donor hearts, which were rejected for transplantation, served as a control group (D1–D9 in Table 1). Samples from three diseased hearts were collected at the time of heart transplant as approved by the IRB at the University of California, Los Angeles, and these comprised the CM group (CM1, CM2, and CM5 in Table 1). CM1 and CM2 had non‐ischemic CM (NICM), and CM5 had ischemic CM (ICM). Donor demographics and brief histories are provided in Table 1.

**TABLE 1:** Donor information.

Donor #	Age	Sex	History
D1	71	F	COD: IC/IV hemorrhage, brain edema. Diabetes mellitus and hypertension
D2	49	F	h/o elective resection of L cerebellopontine schwannoma with post‐op course c/b L cerebellar hemorrhage and hydrocephalus; EF 60–65%, mild LAE, no valvular heart disease
D3	48	F	COD: brain anoxia (drug overdose)
D4	58	M	COD: brain anoxia; h/o diabetes (6–10 years), hypertension (>10 years), hemorrhagic CVA, ischemic CVA, NSTEMI
D5	58	F	COD: cardiac arrest, brain anoxia; atrial fibrillation with rapid ventricular response, abnormal ECG
D6	60	F	COD: CVA/stroke; intracranial hemorrhage
D7	66	F	COD: Head trauma, accident
D8	52	M	COD: CVA/stroke, intracranial hemorrhage; troponin negative, EF 65, moderate concentric LVH, grade 1 diastolic dysfunction
D9	60	F	COD: stroke/brain anoxia. EF: 55–60%
CM1	21	F	Non‐ischemic cardiomyopathy, ventricular fibrillation arrest post implantable cardioverter‐defibrillator implantation; severely increased LV size, LV ejection fraction 18%, normal RV size, mildly reduced systolic function, severe LA enlargement, moderate mitral valve regurgitation
CM2	31	M	h/o asthma and NICM. Echo: mild inc LV size, EF 18%, nl RV size and mild red fxn, severe LAE, mild RAE, mild‐mod MR, mild TR<PASP 53
CM5	75	M	Coronary artery disease, ventricular fibrillation arrest, ICD implant, ischemic cardiomyopathy (~30%); LM, LAD, LCx stents; mild disease in LAD and RCA; Sev inc LV size, LVEF 16%, mild enl RV, mild aortic sclerosis, mild mitral and tricuspid regurgitation

### 2.2 Cardiac imaging, dissection, and tissue processing

Two hearts (D1 and D2) were perfusion fixed at 4°C for 24 h with 4% paraformaldehyde in 0.01M phosphate‐buffered saline (PBS, pH 7.3) while immersed in cold fixative. Hearts were next perfused 4 times for 30 min each with cold PBS to remove fixative and then stored in cold PBS with 0.02% sodium azide at 4°C until further processing. Hearts then underwent CT scanning (SOMATOM Definition AS; Siemens Healthcare, Forchheim, Germany) and gross photography using a Nikon D850 DSLR camera with Nikon AF Micro‐NIKKOR 200 mm f/4D IF‐ED lens. Adjacent sections were dissected and processed for either immunohistochemistry or tissue clearing. Dissected samples from D1 and D2 were transferred to cold PBS containing 20% sucrose and 0.02% sodium azide before shipping to East Tennessee State University (ETSU) for immunohistochemical study. All other samples were dissected from unfixed hearts, immersion fixed at 4°C for 24 h with 4% paraformaldehyde in PBS, washed in cold PBS, and transferred to cold PBS containing 20% sucrose and 0.02% sodium azide before shipping to ETSU for immunohistochemical study. Upon receipt, samples were stored in the cryoprotectant solution at 4°C for 3–7 days (including time since shipping). Samples were then frozen on powdered dry ice and stored in capped tubes at −80°C until sectioning. Frozen samples were attached to specimen plates with OCT compound (Ted Pella, Inc., Redding, CA), and 30 μm sections were cut at −20 to −25°C using a Leica CM3050S cryostat (Leica Microsystems Inc., Bannockburn, IL). Adjacent sections were collected on separate charged slides in a manner producing several sets of sections that each spanned the depth of the sample. After the sections dried, slide sets were transferred to separate slide boxes, wrapped in aluminum foil, and stored at −80°C until staining.

### 2.3 Immunohistochemistry

Boxed slides were equilibrated at room temperature, and one set of slides per sample was used for immunohistochemical identification of TH, VAChT, or PGP9.5 (Table 2). An additional set of slides from the sinoatrial node (SAN) samples was stained for connexin 43 (Cx43) (Table 2) to distinguish nodal tissue from surrounding right atrium (RA). For most regions, each set contained ~4–6 slides, generally with at least two sections per slide. More slides were used for identification of the SAN. Staining was done at room temperature using the Avidin‐Biotin Complex (ABC) immunohistochemistry method (Rabbit ABC‐horseradish peroxidase (HRP) Kit, PK‐4001, Vector Labs) as described previously (Ajijola et al., 2017; Aras et al., 2022). Briefly, slides were rinsed with PBS (pH 7.3), incubated for 10 min in PBS containing 0.4% Triton X‐100 and 0.5% bovine serum albumin (BSA), treated for 15 min with 1.0% H_2_O_2_ in PBS, rinsed with PBS, and incubated 10 min in PBS containing 0.4% Triton X‐100 and 0.5% BSA. Slides were then placed in an incubation box and covered with blocking buffer comprising PBS with 1% BSA, 0.4% Triton X‐100, and normal goat serum (Jackson ImmunoResearch Laboratories, Cat. No. 005‐000‐121). After 2 h, the blocking buffer was replaced with fresh blocking buffer containing one of the primary antibodies (Table 2) and incubated overnight at room temperature. Sections were washed with PBS and PBS containing 0.5% BSA, followed by a 2‐h incubation in biotinylated secondary antibody (1:200 dilution) from the kit. Slides were washed again before a 1.5‐h incubation with the ABC reagent from the kit (Jackson ImmunoResearch 111‐035‐003). Slides were next washed for 20 min in 50 mM Tris buffer (pH 7.6) before treatment for 1–10 min with the chromogen (Vector ImmPACT VIP Kit, SK4605) to visualize targets as a purple reaction product. Slides were washed, dehydrated, and cover glasses were attached using Cytoseal XYL (Thermo Scientific Cat. No. 8312‐4).

**TABLE 2:** Primary antibodies.

Antibody	Host	Immunogen	Company	Catalog No.	RRID	Dilution
Anti‐Tyrosine Hydroxylase (TH)	Rabbit	Native TH from rat pheocromocytoma	Pel‐Freez Biologicals	P40101‐150	RRID: AB_2617184	1:500
	Sheep	Native TH from rat pheocromocytoma	Millipore	AB1542	RRID: AB_90755	1:500
Anti‐Vesicular Acetylcholine Transporter (VAChT)	Rabbit	Recombinant protein, C‐terminal residues of rat VAChT	Synaptic Systems	139103	RRID: AB_887864	1:1000
Anti‐Protein Gene Product 9.5 (PGP9.5)	Rabbit	Synthetic peptide (proprietary)	abcam	ab108986 [EPR4118]	RRID: AB_10891773	1:2000
Anti‐Connexin‐43 (Cx‐43)	Rabbit	Synthetic C‐terminal peptide conjugated to KLH	Sigma‐Aldrich	C6219	RRID: AB_476857	1:2000

### 2.4 Antibody characterization

Primary antibodies used in this study are listed in Table 2 along with the immunogen, host species, company, catalog number, and dilution used. Detailed information regarding the TH antibodies was provided previously (Kaestner et al., 2019). Both antibodies gave identical results, and rabbit anti‐TH was used in most experiments for consistency. The VAChT antibody is the most widely used in the literature for labeling cholinergic neurons, especially nerve fibers, and it has been used by us for several recent studies (Downs et al., 2016; Hanna et al., 2021). It has been knockout validated and labels glycosylated and unglycosylated forms in Western blotting. The antibody to protein gene product 9.5 (PGP9.5) is a rabbit monoclonal with extensive use for labeling central and peripheral neurons, including our recent study of porcine intrinsic cardiac ganglia (Hanna et al., 2021). It labels a single band of the expected size in Western blotting experiments, and this band is absent in a PGP9.5 knockout cell line. The connexin antibody has been used widely to label gap junctions in the heart. It labels a single band in Western blotting, and this band is eliminated by preabsorbing with Cx43 peptide.

### 2.5 Tissue clearing

Specimens from nonfailing and CM human hearts underwent tissue clearing with the modified immunolabeling‐enabled three‐Dimensional Imaging of Solvent‐Cleared Organs (iDISCO+) protocol as previously described (Hanna et al., 2021). Nerve bundles and noradrenergic nerve fibers were identified using antibodies to the pan‐neuronal marker PGP9.5 and the sympathetic marker TH, respectively. An antibody for a cholinergic marker that is compatible with tissue clearing was not available. Autofluorescence was used to visualize the myocardium.

### 2.6 Image acquisition and analysis

Stained immunohistochemical sections were viewed with an Olympus BX41 microscope equipped with a motorized stage, an Olympus DP74 digital camera, and cellSens software (Olympus America Inc., Center Valley, PA; RRID:SCR_016238). For each region of interest, we obtained 20× images from six to nine unique fields over about three sections. To get the best focus, we first acquired a Z‐stack using the system‐recommended step size. Then the Z‐stack was processed using the reflected light algorithm of the extended focus imaging feature. This created the final image by selecting the most sharply focused pixels across the Z‐stack. These images were used to quantify nerve density with ImageJ Software (NIH, Bethesda, MD). Nerve density was calculated as the area occupied by nerves as a percentage of the entire image area and is reported as % area.

Full scans were obtained for selected sections using the Stage Navigator function of cellSens software. This function generates a seamless montage of the stained section from numerous 10× images covering the entire area.

Each iDISCO+‐cleared tissue specimen was placed in a chamber (SunJin Lab) filled with benzyl ether on a slide, and a coverslip was applied. Tilescan and Z stack images were obtained using a Zeiss LSM 880 confocal laser scanning microscope with a Fluor 5×/0.25M27 Plan‐Apochromat objective lens. Images were obtained at a resolution of 1024 × 1024 using 488, 561, and 633 nm laser lines. Z‐axis step size was commensurate with Nyquist sampling based on the numerical aperture of the specified objective. Pinhole was set to 1 airy unit. Stitched images were analyzed in Zeiss Zen Black SR.

### 2.7 Statistical analysis

Data analysis and generation of graphs were performed using GraphPad Prism, Version 8.4.3 (GraphPad Software, San Diego, CA). Nerve density values for each cardiac region of samples from individual donors are presented as the mean ± SD determined from each image. In cases where values for a specific region were available from three or more donors, the mean values for the individual donors were used for statistical comparisons. Between‐group comparisons were made using an unpaired or paired *t* test, as appropriate. *p* < 0.05 was considered statistically significant.

## 3 RESULTS

### 3.1 Quantitative regional analysis of cholinergic and noradrenergic innervation of control hearts and hearts with cardiomyopathy

**FIGURE 1:**
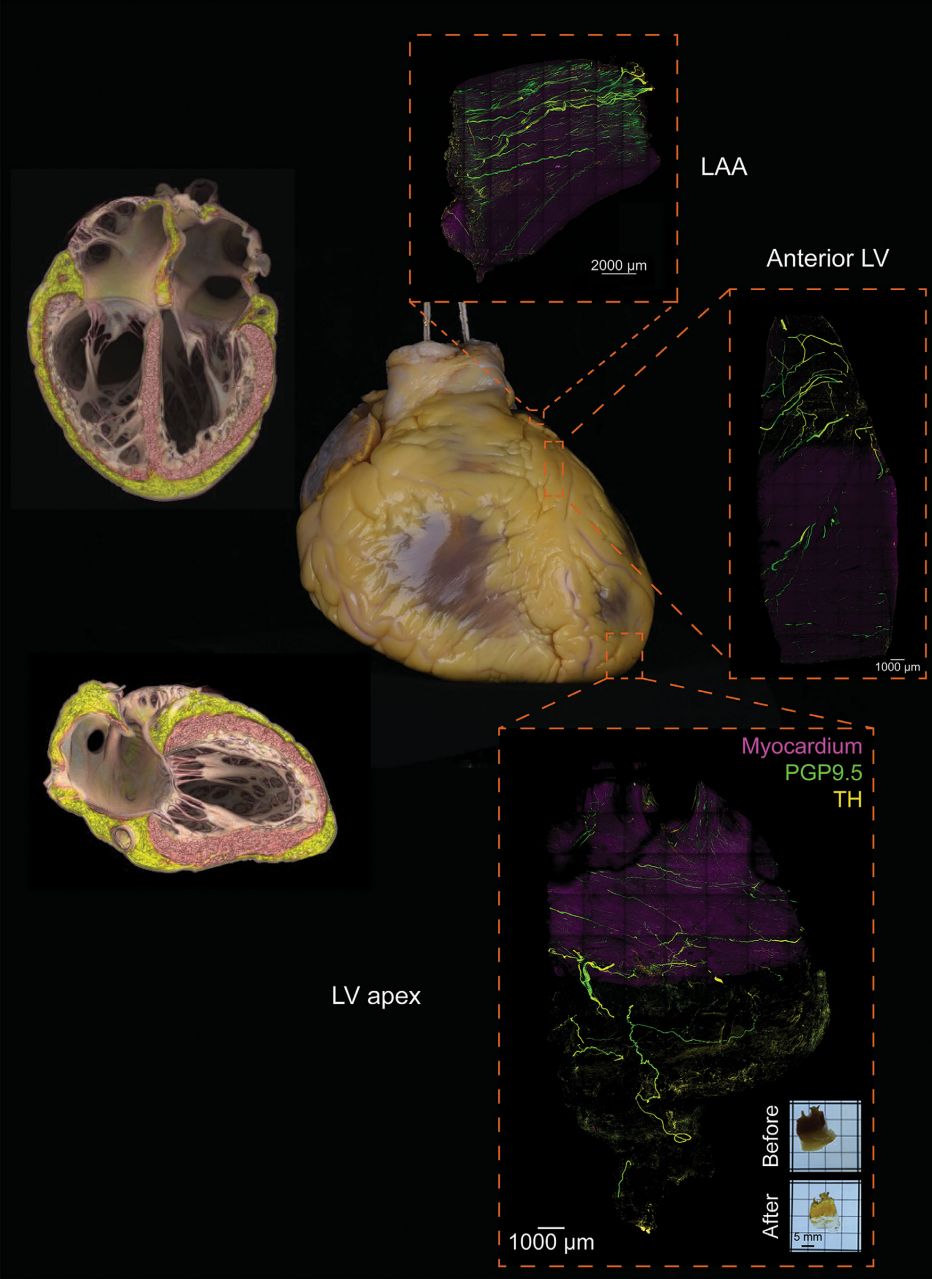
Pipeline of imaging of human hearts and tissue processing. Gross photograph (center panel) and CT scan (left panels) of nonfailing human hearts were used to develop a scaffold for tissue sampling. Adjacent sections were submitted for immunohistochemistry and tissue clearing with iDISCO+. Representative images from the base of the left atrial appendage (upper right panel), basal anterior left ventricle (middle right panel), and left ventricular apex (lower right panel) are shown. Representative photographs of the left ventricular apical sample before and after tissue clearing are shown in the lower right panel.

Extensive sampling and analysis of immunohistochemical sections of atrial and ventricular regions were performed for two hearts judged to be normal based on clinical history (D1 and D2; Table 1), two hearts with NICM (CM1 and CM2; Table 1), and one heart with ICM (CM3; Table 1). Following perfusion fixation, the nonfailing hearts underwent photographic imaging and CT scanning prior to dissection (Figure 1). Additional control data for select atrial and left ventricular regions were obtained using samples collected from seven other donor hearts (D3–D9; Table 1).

### 3.2 Innervation of atrial regions in control hearts

Cholinergic and noradrenergic innervation was evaluated in 12 regions across RA and left atrium (LA), including the SAN (Table 3).

**TABLE 3:** Quantification of nerve densities in atrial regions of human heart.

Region	Control		Non‐ischemic cardiomyopathy		Ischemic cardiomyopathy
	D1	D2	CM1	CM2	CM5
Sinoatrial node (SAN)					
TH	1.76 ± 0.29	1.81 ± 0.45			
VAChT	3.80 ± 0.36	2.22 ± 0.52			
PGP9.5	4.61 ± 0.67	nd			
Right atrium near SAN					
TH	1.43 ± 0.38	1.11 ± 0.33			
VAChT	1.15 ± 0.42	0.46 ± 0.09			
PGP9.5	0.89 ± 0.20	nd			
Crista terminalis					
TH	1.43 ± 0.38	1.33 ± 0.14			
VAChT	1.15 ± 0.42	0.74 ± 0.14			
PGP9.5	0.89 ± 0.20	nd			
Sinus venarum					
TH	0.97 ± 0.31	0.61 0.26			
VAChT	0.82 ± 0.27	0.43 ± 0.18			
PGP9.5	1.84 ± 0.62	0.59 ± 0.34			
Right atrial trabecular muscle					
TH	1.03 ± 0.28	0.86 ± 0.29	2.74 ± 1.03	0.91 ± 0.48	
VAChT	0.74 ± 0.42	1.23 ± 0.45	1.70 ± 0.44	0.28 ± 0.09	
PGP9.5	1.27 ± 0.38	2.14 ± 0.41	3.46 ± 0.44	1.55 ± 0.41	
Right atrial appendage					
TH	1.31 ± 0.32	0.89 ± 0.25	4.66 ± 0.81	2.18 ± 0.74	3.42 ± 0.86
VAChT	0.61 ± 0.30	1.52 ± 0.38	1.23 ± 0.23	0.99 ± 0.24	1.95 ± 0.56
PGP9.5	0.98 ± 0.54	3.00 ± 0.54	3.88 ± 2.04	1.93 ± 0.81	nd
Left atrial posterior wall					
TH	0.27 ± 0.09				
VAChT	0.62 ± 0.31				
PGP9.5	0.96 ± 0.38				
Left atrium near pulmonary vein					
TH		0.48 ± 0.41			
VAChT		1.09 ± 0.39			
PGP9.5		1.73 ± 0.60			
Ligament of marshal					
TH	0.60 ± 0.27	0.73 ± 0.28			
VAChT	0.36 ± 0.10	0.68 ± 0.21			
PGP9.5	1.15 ± 0.46	0.99 ± 0.27			
Base of left atrial appendage					
TH	0.62 ± 0.31	0.35 ± 0.17	1.47 ± 0.68	0.29 ± 0.10	
VAChT	0.46 ± 0.21	0.36 ± 0.14	0.62 ± 0.36	0.29 ± 0.12	
PGP9.5	1.79 ± 0.63	0.91 ± 0.32	1.83 ± 0.37	1.35 ± 0.37	
Left atrial appendage					
TH	1.49 ± 0.50	0.69 ± 0.22	2.31 ± 0.34	1.05 ± 0.36	0.08 ± 0.14
VAChT	0.64 ± 0.43	1.03 ± 0.29	0.66 ± 0.32	0.18 ± 0.13	0.06 ± 0.08
PGP9.5	2.04 ± 0.73	2.03 ± 0.62	2.25 ± 0.45	0.86 ± 0.32	nd

*Note*: Innervation density is expressed as % Area (nerve area as % of total area in field). Values are mean ± SD for images from 6 to 9 fields per sample. nd, not determined. Samples were not available for blank cells.

**FIGURE 2:**
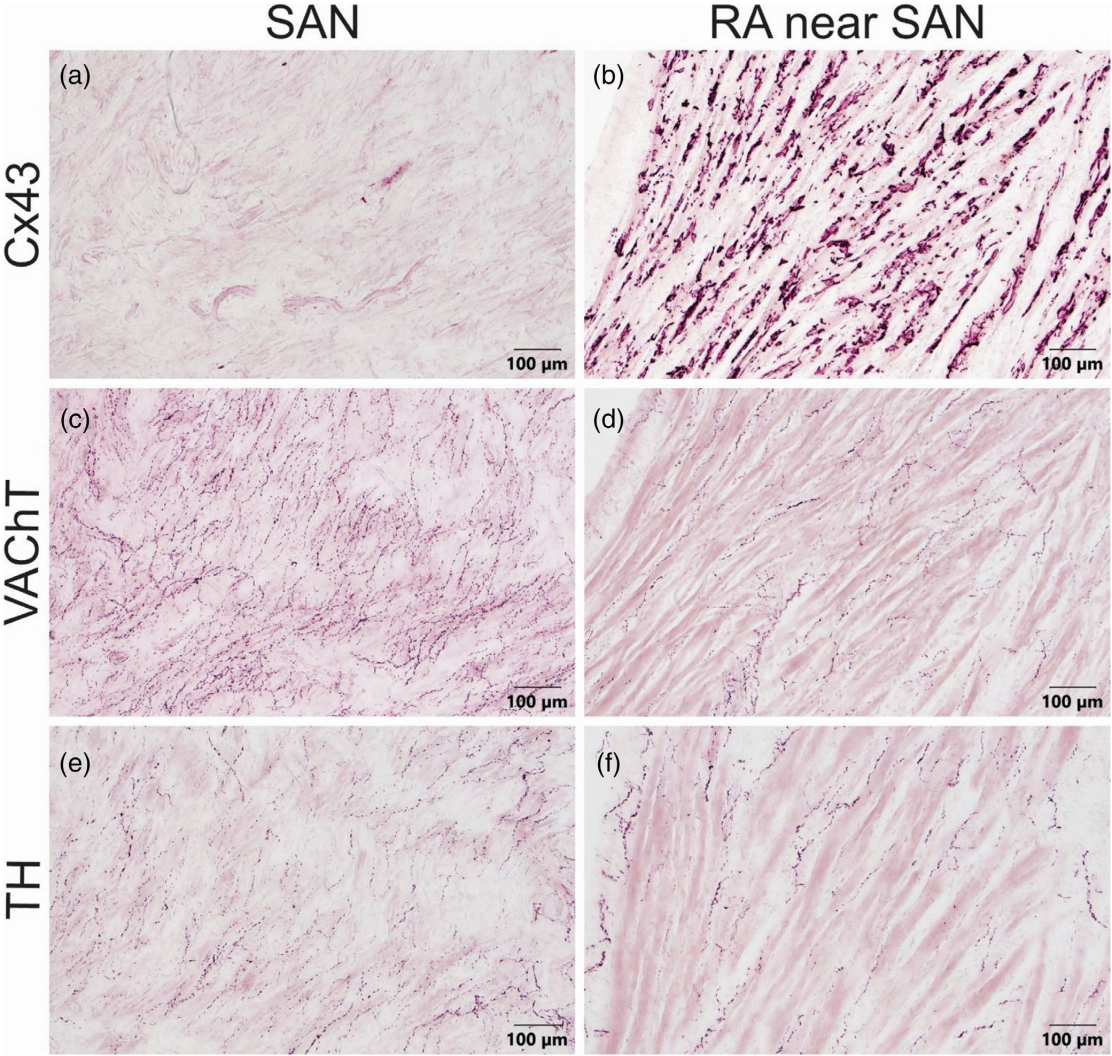
Photomicroscopic images showing staining for Cx43, VAChT, and TH in the SAN and nearby RA. (a, b) Nodal cells lack Cx43 (a) but RA muscle stains intensely for this marker (b). (c, d) VAChT+ cholinergic nerve fibers are more abundant in the SAN compared to surrounding RA muscle. (e, f) TH+ noradrenergic nerve fibers occur at similar density in the node and surrounding RA muscle.

**FIGURE 3:**
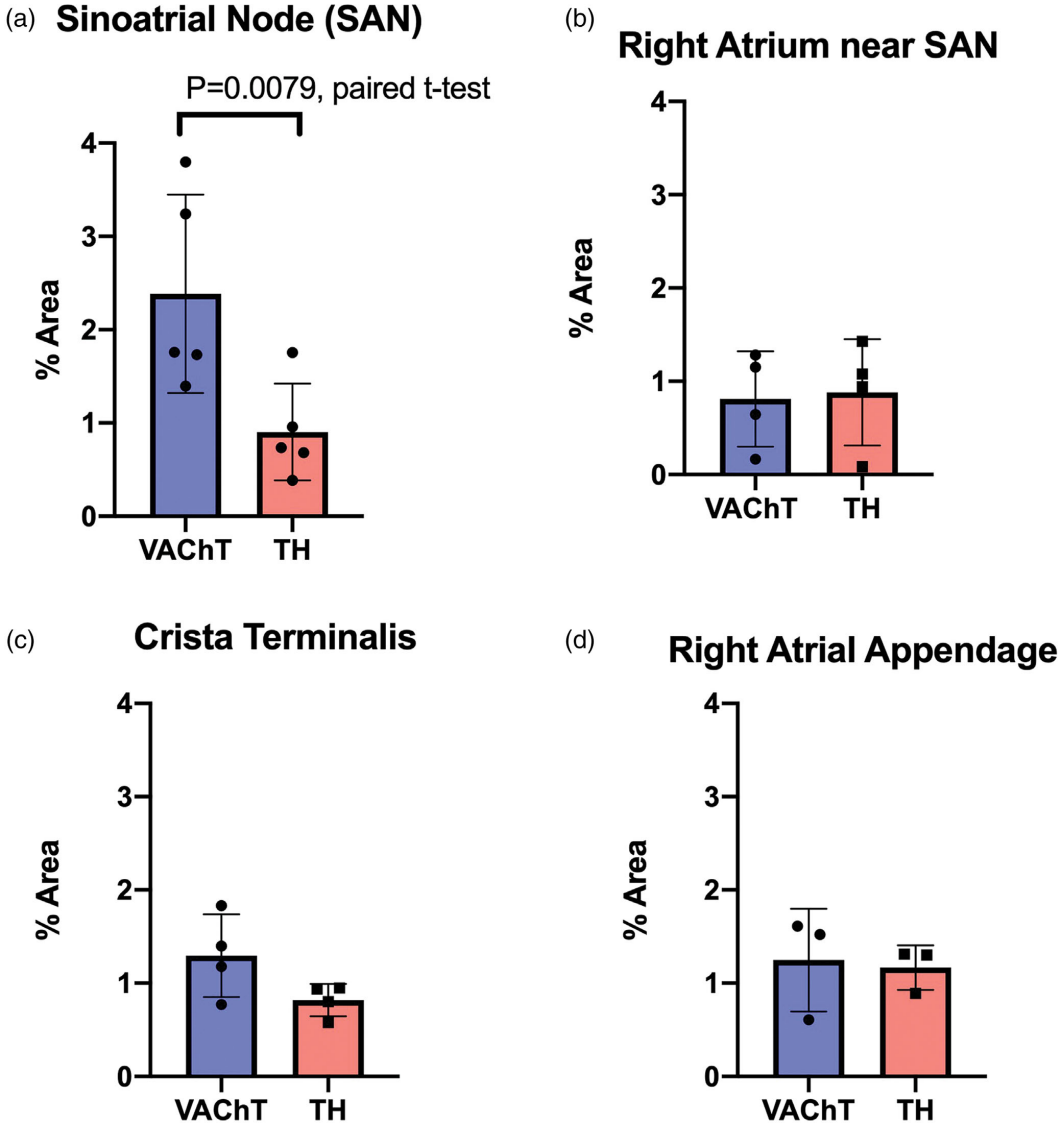
Quantitative analysis of cholinergic and noradrenergic nerve fibers in select right atrial regions. Values are means ± SDs for the number of hearts indicated by points in bar graphs. Each data point was determined from about 6–9 images from at least three sections. Nerve densities are reported as % area of 20× images occupied by stained nerve fibers. For each region, VAChT and TH nerve fiber densities were compared using a paired *t* test. *p* < 0.05 was considered significant.

Cholinergic and noradrenergic nerves were present at a high to moderate density throughout the atria, but there was some regional variation (Table 3). Specifically, both nerve fiber types were generally more abundant on the right side compared to the left (Table 3). The SAN, which was identified based on location and lack of staining for Cx43 (Figures 2a,b and S1, Supporting Information), had the highest density of cholinergic innervation, and VAChT‐positive nerve fibers were significantly more abundant than TH‐positive nerve fibers in this region (Figures 2 and 3a). These nerve fibers were present at similar densities (i.e., cholinergic vs. noradrenergic) in other RA and LA sites (Figures 2d,f and 3b–d and Table 3). One exception appears to be atrial muscle around pulmonary veins, where cholinergic nerve fibers were more abundant than noradrenergic nerve fibers (Figure S2). However, we only had one sample from this region to evaluate. Interestingly, the pulmonary venous ostium received abundant noradrenergic input, but VAChT fibers were very sparse (Figure S2e,f). Several small‐ to medium‐sized ganglia were detected in epicardial fat around the pulmonary vein (Figure S3). Cell bodies in these ganglia stained for VAChT and PGP9.5, and they all received abundant input by varicose cholinergic nerve fibers. Noradrenergic nerve fibers traveled by or through these ganglia, and in some cases formed a varicose plexus around cell bodies.

PGP9.5+ nerve fibers were abundant throughout the atria (Figure 1), where they usually occurred at a greater density than either cholinergic or noradrenergic nerves (Table 3). The density of nerves labeled for this pan‐neuronal marker often approximated the sum of cholinergic and noradrenergic nerves. Scattered atrial myocytes in some but not all regions of control hearts had light to moderate staining for PGP9.5 (see section 3.3).

### 3.3 Atrial innervation in cardiomyopathy

**FIGURE 4:**
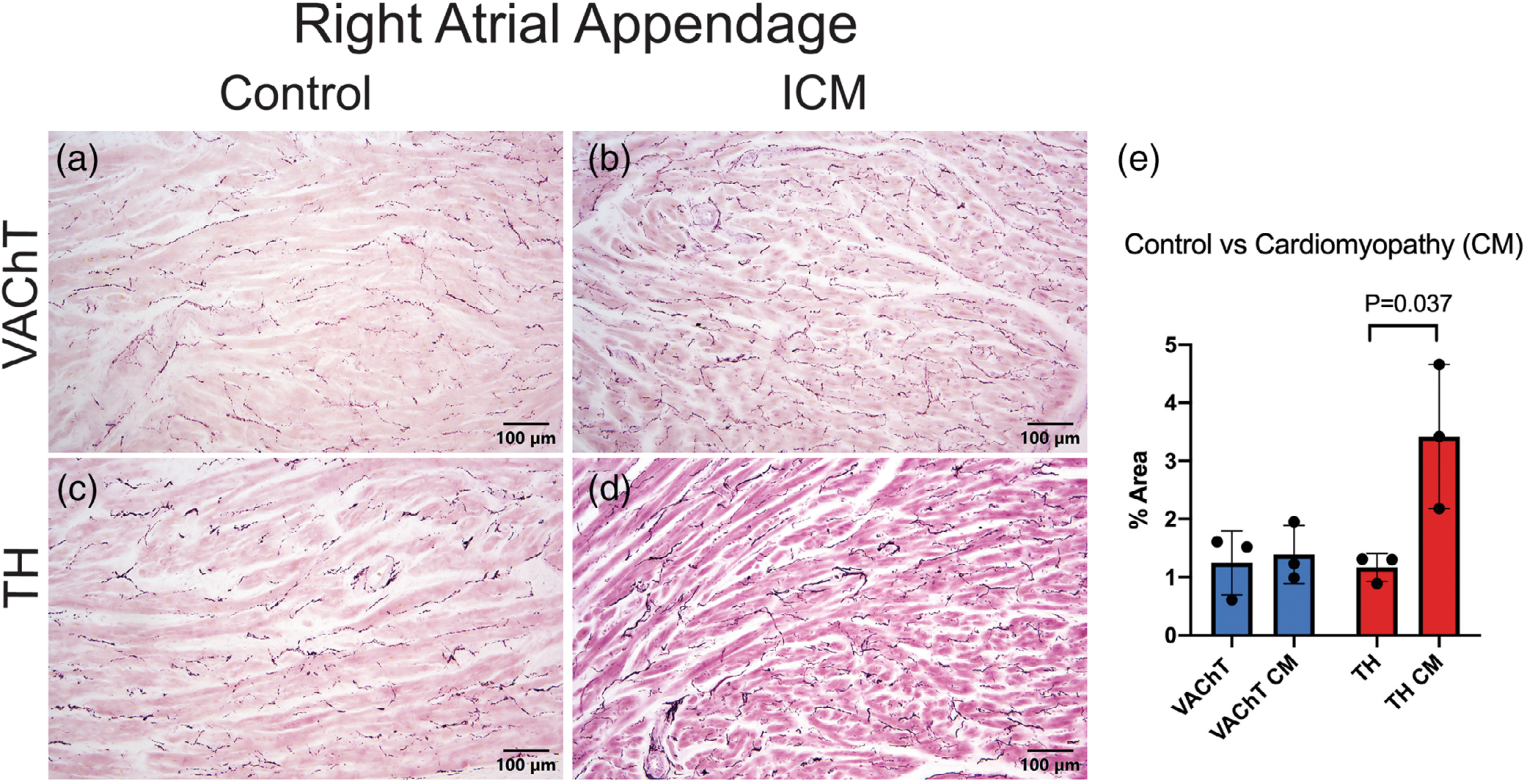
Effect of cardiomyopathy on innervation of human right atrial appendage. (a–d) Representative images showing cholinergic (VAChT) and noradrenergic (TH) nerve fibers in right atrial appendage from a control heart and one with ischemic cardiomyopathy (ICM). (e) Quantitative analysis of nerve fiber densities in tissue from three control hearts and three with cardiomyopathy (2 non‐ischemic and 1 ischemic). Values are means ± SDs. *p* < 0.05 was considered significant.

**FIGURE 5:**
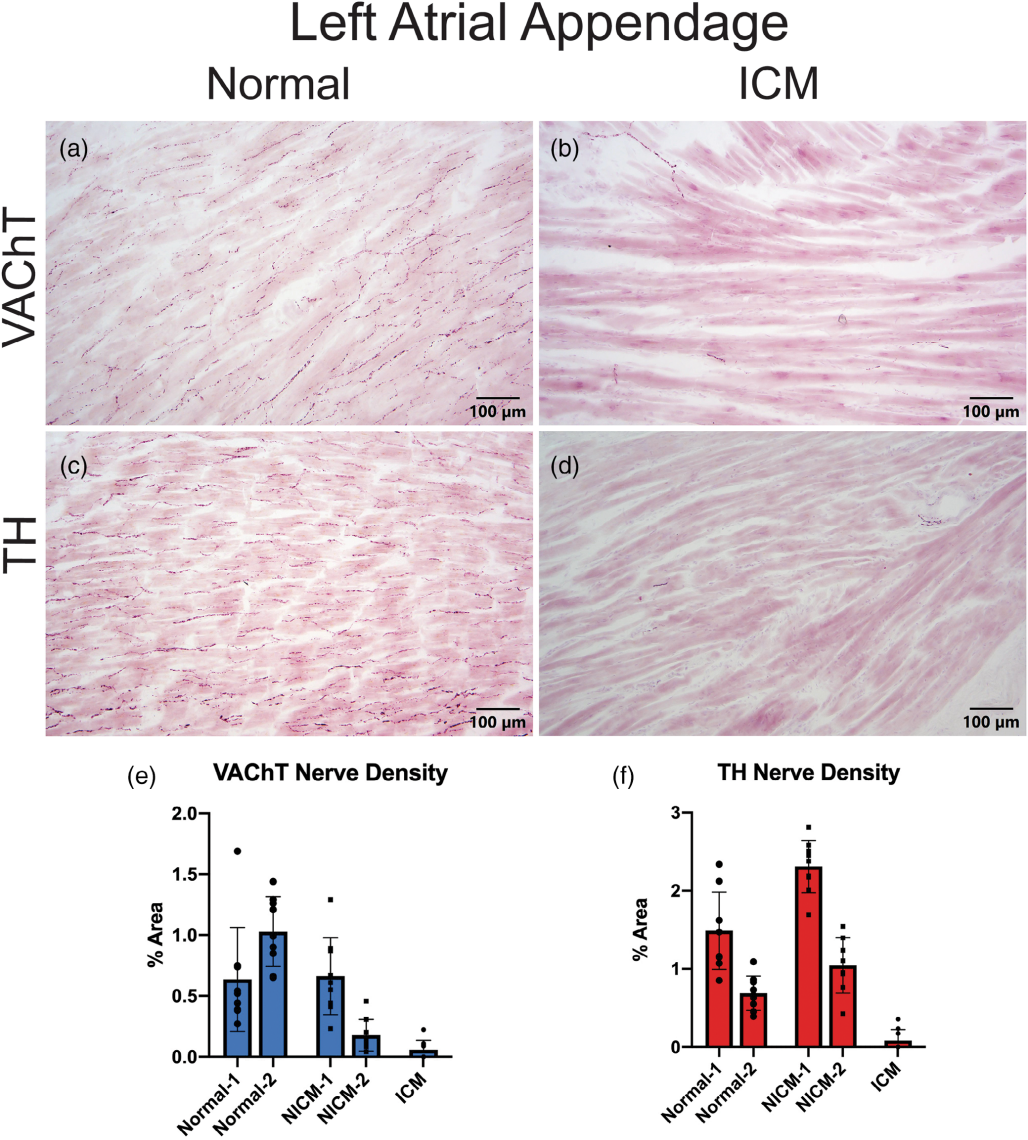
Effect of cardiomyopathy on innervation of human left atrial (LA) appendage. (a–d) Representative images showing cholinergic (VAChT) and noradrenergic (TH) nerve fibers in LA appendage from a control heart and one with ischemic cardiomyopathy (ICM). Note the marked loss of both nerve fiber types in the tissue from an ICM heart. (e, f) Quantitative analysis of nerve fiber densities in tissue from five control hearts and three with cardiomyopathy (2 non‐ischemic and 1 ischemic). Control values are means ± SD for average density for five donors. CM values are shown for each donor as the means ± SD for nine images per donor.

Fewer atrial samples were collected in the CM cases due to the surgical technique used on the recipient hearts, as these patients were undergoing transplant. Two major differences in atrial innervation were noted for these hearts. First, noradrenergic nerve fiber density was increased substantially in RA appendage samples from all cardiomyopathy cases compared to controls (Figure 4). These patients had large reductions of EF (Table 1). In marked contrast, no clear differences in cholinergic innervation density were detected, although VAChT‐positive nerve fibers appeared more abundant in the ICM (CM5) RA appendage (Figure 4a,b). Second, there was a marked loss of cholinergic and noradrenergic nerve fibers in the LA appendage from the ICM heart compared to controls and samples from NICM hearts, and one of the NICM hearts also had a marked loss of cholinergic nerve fibers (Figure 5).

### 3.4 Ganglionated plexuses in epicardial fat pads

**FIGURE 6:**
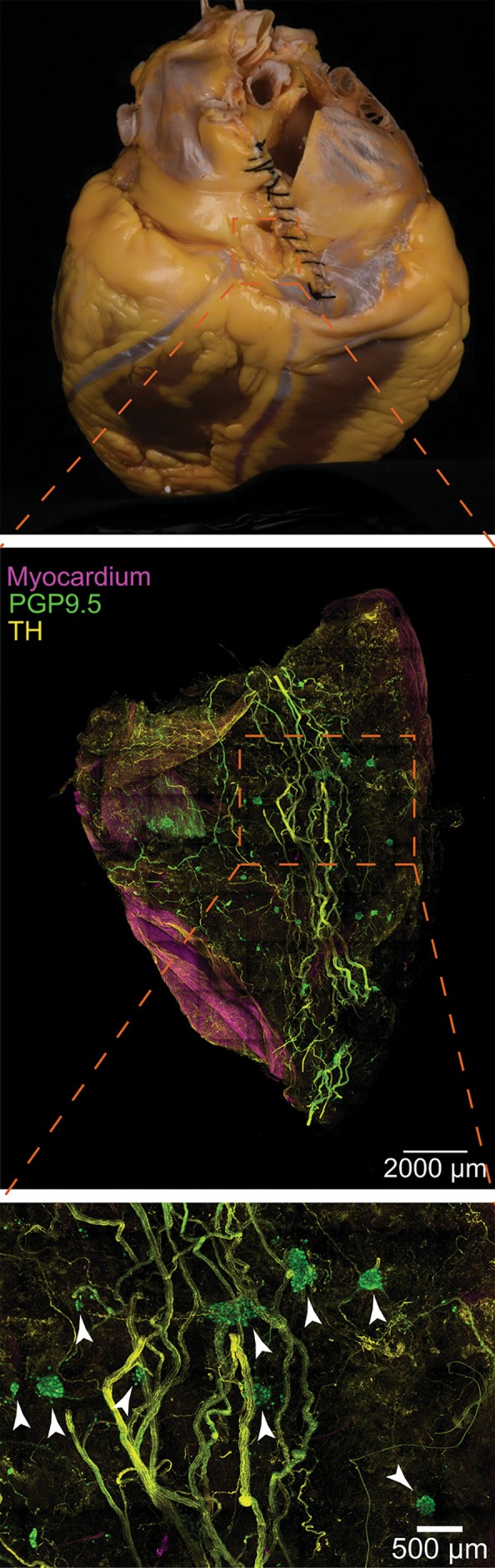
Ganglionated plexuses are founded embedded in epicardial fat pads. Upper panel, posterior–anterior photographic view of region of epicardial fat near the crux of the heart that was dissected. Middle panel, iDISCO+‐cleared image of inferior vena cava‐inferior atrium ganglionated plexus with multiple PGP9.5+ ganglia and nerve fibers (green) interspersed with TH+ (yellow) nerve bundles. Lower panel, magnified image of ganglia (white arrowheads) within the inferior vena cava‐inferior atrium ganglionated plexus.

Tissue‐clearing of epicardial fat pads was used to visualize the ganglionated plexuses. For example, at the crux of the heart, the inferior vena cava–inferior atrial ganglionated plexus was dissected, cleared, and imaging demonstrated several ganglia with large TH+ fibers traversing the fat pad (Figure 6).

### 3.5 Innervation of ventricular regions in control hearts

Cholinergic and noradrenergic innervation were evaluated initially in two perfusion‐fixed control hearts (D1 and D2 in Table 1), using identical methods to those applied to atrial samples collected from the same hearts. Staining and nerve fiber quantification were performed for four areas of right ventricle (RV), three of ventricular septum, seven of left ventricular (LV) free wall, and a papillary muscle (Table 4). Results for ventricular myocardium were more variable than observed with the atria, especially for regions of LV septum and LV free wall. Furthermore, nerve fiber densities were far less for LV free wall samples from D2 compared to D1. Nevertheless, several important aspects of ventricular innervation were revealed.

**TABLE 4:** Quantification of nerve densities in ventricular regions of human hearts.

Region	Control					Non‐ischemic cardiomyopathy		Ischemic cardiomyopathy
	D1	D2	D7	D8	D9	CM1	CM2	CM3
Right ventricular inflow tract (RVIT)								
Subepicardium								
TH	1.03 ± 0.18	0.29 ± 0.18				1.69 ± 0.18	1.27 ± 0.21	0.53 ± 0.07
VAChT	0.21 ± 0.18	0.29 ± 0.18				0.43 ± 0.33	0.15 ± 0.09	0.10 ± 0.03
PGP9.5	1.49 ± 0.34	0.61 ± 0.20				1.86 ± 0.36	1.00 ± 0.09	0.67 ± 0.27
Subendocardium								
TH	1.09 ± 0.50	0.41 ± 0.28				1.22 ± 0.35	1.49 ± 0.25	1.27 ± 0.77
VAChT	0.63 ± 0.37	0.25 ± 0.13				0.42 ± 0.12	0.19 ± 0.04	0.06 ± 0.01
PGP9.5	1.73 ± 0.47	0.98 ± 0.29				1.70 ± 0.45	1.29 ± 0.06	1.26 ± 0.25
Right ventricular outflow tract (RVOT)								
Subepicardium								
TH	0.72 ± 0.22	0.28 ± 0.18				0.99 ± 0.01	1.44 ± 0.09	0.69 ± 0.26
VAChT	0.17 ± 0.10	0.18 ± 0.04				0.47 ± 0.09	0.13 ± 0.06	0.06 ± 0.01
PGP9.5	1.46 ± 0.22	0.56 ± 0.09				1.42 ± 0.09	0.95 ± 0.07	0.38 ± 0.08
Subendocardium								
TH	1.39 ± 0.18	0.40 ± 0.15				2.04 ± 0.03	1.62 ± 0.40	0.49 ± 0.20
VAChT	0.22 ± 0.11	0.25 ± 0.11				0.31 ± 0.13	0.24 ± 0.07	0.08 ± 0.04
PGP9.5	1.71 ± 0.64	0.62 ± 0.29				1.43 ± 0.11	0.94 ± 0.03	0.54 ± 0.16
Basal RV free wall								
Subepicardium								
TH	0.34 ± 0.18	0.59 ± 0.18				2.39 ± 0.37	1.22 ± 0.22	0.80 ± 0.14
VAChT	0.05 ± 0.03	0.15 ± 0.05				0.19 ± 0.08	0.15 ± 0.05	0.02 ± 0.03
PGP9.5	0.88 ± 0.40	1.00 ± 0.24				1.87 ± 0.21	0.79 ± 0.03	0.59 ± 0.08
Midwall								
TH	0.32 ± 0.17	0.37 ± 0.14				2.25 ± 0.59	0.95 ± 0.02	1.25 ± 0.32
VAChT	0.18 ± 0.04	0.12 ± 0.04				0.18 ± 0.01	0.06 ± 0.05	0.02 ± 0.02
PGP9.5	1.67 ± 0.28	0.91 ± 0.01				1.85 ± 0.55	0.98 ± 0.15	0.84 ± 0.31
Subendocardium								
TH	1.07 ± 0.38	0.94 ± 0.08				1.20 ± 0.22	1.00 ± 0.39	0.62 ± 0.26
VAChT	0.12 ± 0.04	0.20 ± 0.03				0.08 ± 0.03	0.12 ± 0.04	0.01 ± 0.02
PGP9.5	1.95 ± 0.33	1.34 ± 0.10				1.13 ± 0.28	0.67 ± 0.18	0.84 ± 0.15
Right ventricular apex								
Subepicardium								
TH	1.14 ± 0.20	0.31 ± 0.17				1.24 ± 0.16	0.56 ± 0.02	0.56 ± 0.13
VAChT	0.12 ± 0.07	0				0.10 ± 0.06	0.03 ± 0	0.01 ± 0.01
PGP9.5	1.88 ± 0.50	0.39 ± 0.17				2.26 ± 0.58	0.58 ± 0.11	0.17 ± 0.03
Midwall								
TH	0.57 ± 0.42	0.39 ± 0.27				1.26 ± 0.29	0.69 ± 0.52	0.84 ± 0.67
VAChT	0.09 ± 0.05	0				0.08 ± 0.06	0.01 ± 0.01	0.02 ± 0.02
PGP9.5	1.80 ± 0.47	0.42 ± 0.29				1.84 ± 0.38	0.48 ± 0.66	0.62 ± 0.81
Subendocardium								
TH	1.07 ± 0.38	0.66 ± 0.10				1.20 ± 0.22	0.96 ± 0.23	1.28 ± 0.76
VAChT	0.10 ± 0.04	0				0.12 ± 0.06	0.02 ± 0.02	0.01 ± 0.02
PGP9.5	2.24 ± 0.25	0.81 ± 0.44				1.51 ± 0.57	0.17 ± 0.26	0.48 ± 0.32
Basal ventricular septum								
Subendocardium								
TH	0.58 ± 0.09	0.42 ± 0.03				0.51 ± 0.21	0.92 ± 0.11	nd
VAChT	0.03 ± 0.04	0.12 ± 0.08				0.03 ± 0.04	0.03 ± 0.04	nd
PGP9.5	1.85 ± 0.59	0.66 ± 0.31				0.60 ± 0.20	0.78 ± 0.01	nd
Mid ventricular septum								
Subendocardium								
TH	0.63 ± 0.03	0.31 ± 0.08				0.76 ± 0.18	0.70 ± 0.21	nd
VAChT	0.06 ± 0.06	0.05 ± 0.04				0.01 ± 0.01	0.01 ± 0.01	nd
PGP9.5	2.42 ± 0.43	0.78 ± 0.50				0.83 ± 0.43	0.99 ± 0.34	nd
Apical septum								
Subendocardium								
TH	0.80 ± 0.52	0.49 ± 0.01				0.88 ± 0.26	0	nd
VAChT	0.19 ± 0.13	0				0.13 ± 0.06	0.03 ± 0.01	nd
PGP9.5	1.28 ± 0.59	0.72 ± 0.43				0.83 ± 0.33	0.23 ± 0.20	nd
Basal LV free wall, anterior								
Subepicardium								
TH	0.39 ± 0.05	0.11 ± 0.13				0.70 ± 0.08	1.15 ± 0.19	0
VAChT	0.20 ± 0.13	0.05 ± 0.04				0.22 ± 0.04	0.11 ± 0.03	0.03 ± 0.03
PGP9.5	1.33 ± 0.06	0.27 ± 0.17				1.18 ± 0.21	1.09 ± 0.02	0.55 ± 0.12
Midwall								
TH	0.51 ± 0.15	0.06 ± 0.91				1.31 ± 0.28	0.97 ± 0.18	0.39 ± 0.16
VAChT	0.16 ± 0.02	0.02 ± 0.04				0.15 ± 0.02	0.11 ± 0.05	0.69 ± 0.21
PGP9.5	1.27 ± 0.25	0				1.38 ± 0.55	0.93 ± 0.11	0.95 ± 0.36
Subendocardium								
TH	0.60 ± 0.18	0.02 ± 0.03				0.83 ± 0.39	0.89 ± 0.26	0.33 ± 0.06
VAChT	0.17 ± 0.06	0				0.08 ± 0.01	0.09 ± 0.06	0.03 ± 0.05
PGP9.5	1.17 ± 0.33	0.18 ± 0.05				1.05 ± 0.13	2.04 ± 0.22	0.72 ± 0.35
Basal LV free wall, lateral								
Subepicardium								
TH	0.5 ± 0.38	0.15 ± 0.07	1.79 ± 0.76	2.23 ± 0.23	0.86 ± 0.57	1.43 ± 0.04	1.46 ± 0.14	0.35 ± 0.20
VAChT	0.09 ± 0.03	0.15 ± 0.04	0.45 ± 0.19	0.42 ± 0.25	0.04 ± 0.04	0.41 ± 0.02	0.04 ± 0.02	0.03 ± 0.03
PGP9.5	1.34 ± 0.10	0.51 ± 0.15	2.06 ± 0.50	1.62 ± 0.45	1.55 ± 0.49	0.82 ± 0.16	1.09 ± 0.22	0.51 ± 0.25
Midwall								
TH	0.18 ± 0.05	0.06 ± 0.02	1.42 ± 0.41	2.69 ± 0.70	1.08 ± 0.70	1.52 ± 0.20	1.01 ± 0.16	0.72 ± 0.28
VAChT	0.08 ± 0.06	0.17 ± 0.08	0.48 ± 0.14	0.46 ± 0.20	0.14 ± 0.05	0.29 ± 0.13	0.11 ± 0.05	0.07 ± 0.07
PGP9.5	1.08 ± 0.12	0.41 ± 0.12	3.12 ± 0.85	1.95 ± 0.49	1.74 ± 0.57	1.49 ± 0.19	1.10 ± 0.06	1.31 ± 0.49
Subendocardium								
TH	0.42 ± 0.16	0.13 ± 0.09	1.08 ± 0.24	1.94 ± 0.90	0.90 ± 0.19	1.71 ± 0.28	1.69 ± 0.70	0.71 ± 0.05
VAChT	0.14 ± 0.02	0.22 ± 0.08	0.32 ± 0.10	0.56 ± 0.70	0.24 ± 0.20	0.26 ± 0.13	0.06 ± 0.02	0.10 ± 0.02
PGP9.5	1.14 ± 0.12	0.36 ± 0.14	2.06 ± 0.50	1.80 ± 0.63	1.80 ± 0.63	1.53 ± 0.23	1.11 ± 0.28	0.89 ± 0.24
Basal LV free wall, posterior								
Subepicardium								
TH	0.14 ± 0.05	0.23 ± 0.10				0.75 ± 0.44	1.35 ± 0.60	0.77 ± 0.04
VAChT	0.47 ± 0.25	0.17 ± 0.50				0.23 ± 0.05	0.12 ± 0.03	0.07 ± 0.02
PGP9.5	1.27 ± 0.19	0.50 ± 0.12				0.84 ± 0.43	1.03 ± 0.22	0.56 ± 0.15
Midwall								
TH	0.14 ± 0.05	0				0.94 ± 0.18	1.00 ± 0.08	0.66 ± 0.34
VAChT	0.18 ± 0.11	0.02 ± 0.03				0.20 ± 0.04	0.08 ± 0.06	0.05 ± 0.04
PGP9.5	1.12 ± 0.20	0.25 ± 0.08				0.81 ± 0.32	0.75 ± 0.19	0.57 ± 0.23
Subendocardium								
TH	1.31 ± 0.34	0.31 ± 0				0.60 ± 0.28	0.60 ± 0.05	1.00 ± 0.30
VAChT	0.14 ± 0.02	0.26 ± 0.10				0.25 ± 0.04	0.08 ± 0.03	0.08 ± 0.04
PGP9.5	1.14 ± 0.12	0.63 ± 0.27				1.38 ± 0.38	1.02 ± 0.65	0.66 ± 0.29
Mid‐LV free wall, anterior								
Subepicardium								
TH	0.32 ± 0.14	0				0.91 ± 0.21	0.98 ± 0.33	0.39 ± 0.15
VAChT	0.16 ± 0.13	0				0.20 ± 0.10	0	nd
PGP9.5	1.15 ± 0.21	0				0.50 ± 0.13	0.39 ± 0.01	nd
Midwall								
TH	0.21 ± 0.05	0.03 ± 0.12				0.76 ± 0.11	0.91 ± 0.23	0.36 ± 0.07
VAChT	0.05 ± 0.04	0.01 ± 0.01				0.11 ± 0.01	0.01 ± 0.02	nd
PGP9.5	1.30 ± 0.18	0.19 ± 0.08				0.56 ± 0.06	0.69 ± 0.18	nd
Subendocardium								
TH	0.79 ± 0.33	0.17 ± 0.02				0.49 ± 0.28	2.20 ± 0.31	0.47 ± 0.18
VAChT	0.18 ± 0.07	0.09 ± 0.03				0.19 ± 0.02	0.01 ± 0.01	nd
PGP9.5	1.45 ± 0.18	0.44 ± 0.24				0.58 ± 0.18	1.07 ± 0.26	nd
Mid‐LV free wall, lateral								
Subepicardium								
TH	0.29 ± 0.08	0.52 ± 0.09	0.97 ± 0.18	2.44 ± 0.49	0.57 ± 0.38	0.95 ± 0.29	0.43 ± 0.06	0.74 ± 0.38
VAChT	0.12 ± 0.06	0.30 ± 0.18	0.18 ± 0.07	0.21 ± 0.02	0.01 ± 0.01	0.21 ± 0.04	0.05 ± 0.06	0.02 ± 0.02
PGP9.5	1.06 ± 0.08	0.48 ± 0.14	3.22 ± 1.13	1.43 ± 0.24	1.03 ± 0.26	1.37 ± 0.32	0.08 ± 0.04	1.16 ± 0.75
Midwall								
TH	0.15 ± 0.02	0.17 ± 0.13	0.88 ± 0.34	1.94 ± 0.30	0.49 ± 0.09	0.94 ± 0.28	0.77 ± 0.41	0.91 ± 0.57
VAChT	0.21 ± 0.08	0.10 ± 0.10	0.16 ± 0.07	0.28 ± 0.16	0.06 ± 0.04	0.15 ± 0.07	0.02 ± 0.03	0.05 ± 0.07
PGP9.5	1.03 ± 0.23	0.31 ± 0.20	1.67 ± 0.37	1.56 ± 0.37	0.81 ± 0.39	0.94 ± 0.13	0.36 ± 0.28	0.70 ± 0.45
Subendocardium								
TH	0.31 ± 0.07	0.35 ± 0.09	0.72 ± 0.31	1.34 ± 0.21	0.68 ± 0.06	0.55 ± 0.04	1.02 ± 0.36	0.68 ± 0.43
VAChT	0.19 ± 0.03	0.19 ± 0.03	0.38 ± 0.16	0.29 ± 0.06	0.25 ± 0.42	0.08 ± 0.07	0.06 ± 0.04	0.03 ± 0.05
PGP9.5	0.75 ± 0.10	0.75 ± 0.39	2.13 ± 0.51	1.53 ± 0.16	1.69 ± 0.61	0.61 ± 0.15	0.97 ± 0.14	0.36 ± 0.27
Mid‐LV free wall, posterior								
Subepicardium								
TH	0.33 ± 0.16	0.22 ± 0.05				0.71 ± 0.11	0.91 ± 0.58	0.71 ± 0.41
VAChT	0.20 ± 0.04	0.17 ± 0.03				0.18 ± 0.12	0.02 ± 0.03	0.01 ± 0.01
PGP9.5	1.03 ± 0.01	0.42 ± 0.10				0.41 ± 0.16	0.50 ± 0.30	0.46 ± 0.20
Midwall								
TH	0	0				0.53 ± 0.08	0.68 ± 0.44	0.18 ± 0.04
VAChT	0.08 ± 0.21	0.15 ± 0.02				0.11 ± 0.07	0.01 ± 0.01	0.01 ± 0.01
PGP9.5	0.98 ± 0.59	0.39 ± 0.10				0.27 ± 0.09	0.78 ± 0.22	0.55 ± 0.11
Subendocardium								
TH	0.40 ± 0.09	0.31 ± 0.21				0.58 ± 0.12	1.28 ± 0.62	0.39 ± 0.36
VAChT	0.11 ± 0.05	0.17 ± 0.09				0.15 ± 0.03	0.22 ± 0.04	0.03 ± 0.05
PGP9.5	1.27 ± 0.40	0.32 ± 0.10				0.85 ± 0.59	0.91 ± 0.31	0.55 ± 0.70
LV apex								
Subepicardium								
TH	0.69 ± 0.16	0.39 ± 0.02	0.62 ± 0.26	1.49 ± 0.56	0.40 ± 0.07	0.68 ± 0.06	0.79 ± 0.39	nd
VAChT	0.03 ± 0.04	0.01 ± 0.01	0.47 ± 0.25	0.15 ± 0.07	0.21 ± 0.13	0.08 ± 0.03	0.01 ± 0.01	nd
PGP9.5	1.45 ± 0.54	0.38 ± 0.05	1.72 ± 0.96	1.99 ± 0.62	1.37 ± 0.44	0.65 ± 0.14	0.63 ± 0.12	nd
Midwall								
TH	0.22 ± 0.08	0	0.87 ± 0.23	1.93 ± 0.64	0.84 ± 0.33	0.49 ± 0.27	1.10 ± 0.14	nd
VAChT	0.05 ± 0.07	0	0.63 ± 0.13	0.22 ± 0.08	0.07 ± 0.11	0.06 ± 0.02	0.05 ± 0.08	nd
PGP9.5	1.38 ± 0.31	0.39 ± 0.10	2.97 ± 0.40	1.92 ± 0.79	1.41 ± 0.40	0.50 ± 0.14	0.98 ± 0.44	nd
Subendocardium								
TH	0.67 ± 0.52	0.37 ± 0.22	1.28 ± 0.41	1.80 ± 0.62	1.16 ± 0.41	0.70 ± 0.03	0.65 ± 0.25	nd
VAChT	0.13 ± 0.11	0.03 ± 0.05	0.32 ± 0.10	0.56 ± 0.70	0.24 ± 0.20	0	0.01 ± 0.01	nd
PGP9.5	1.49 ± 0.30	0.68 ± 0.36	2.13 ± 0.51	1.53 ± 0.16	1.69 ± 0.61	0.49 ± 0.16	0.33 ± 0.06	nd
Papillary muscle								
TH	0.31 ± 0.16	0.30 ± 0.18						
VAChT	0.10 ± 0.09	0.03 ± 0.05						
PGP9.5	1.47 ± 0.50	0.42 ± 0.19						

**FIGURE 7:**
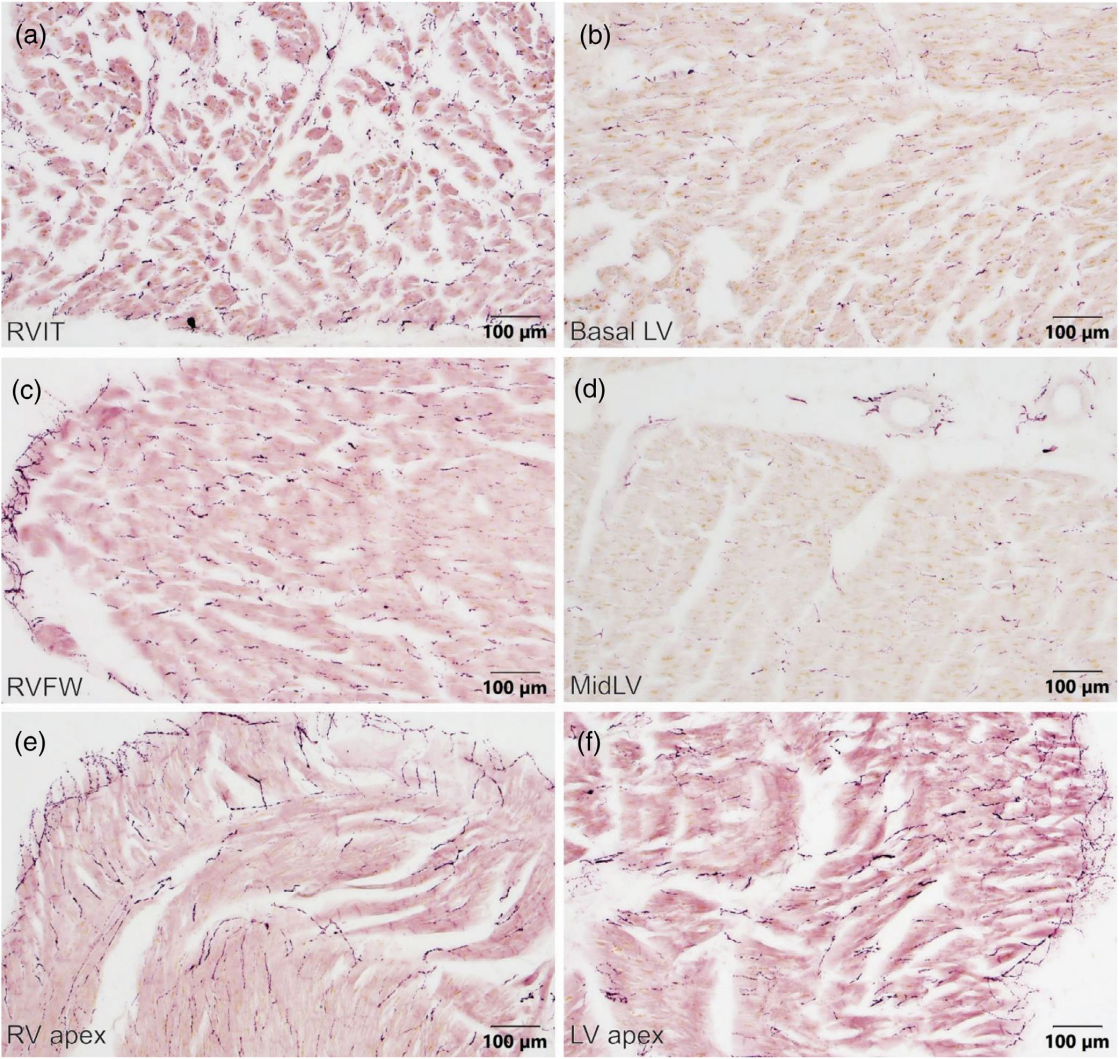
Noradrenergic nerve fibers are present in right and left ventricular regions from the base of the heart to the apex. Representative photomicrographs of sections from D1 heart (control) stained for TH. (a) Right ventricular inflow tract (RVIT) subepicardium. (b) Basal left ventricular (LV) free wall subepicardium. (c) Right ventricular free wall (RVFW) epicardium and subepicardium. (d) Mid‐LV free wall epicardium and subepicardium. Note TH+ nerve fibers innervating epicardial arteries. (e) RV apex epicardium and subepicardium. (f) LV apex epicardium and subepicardium.

**FIGURE 8:**
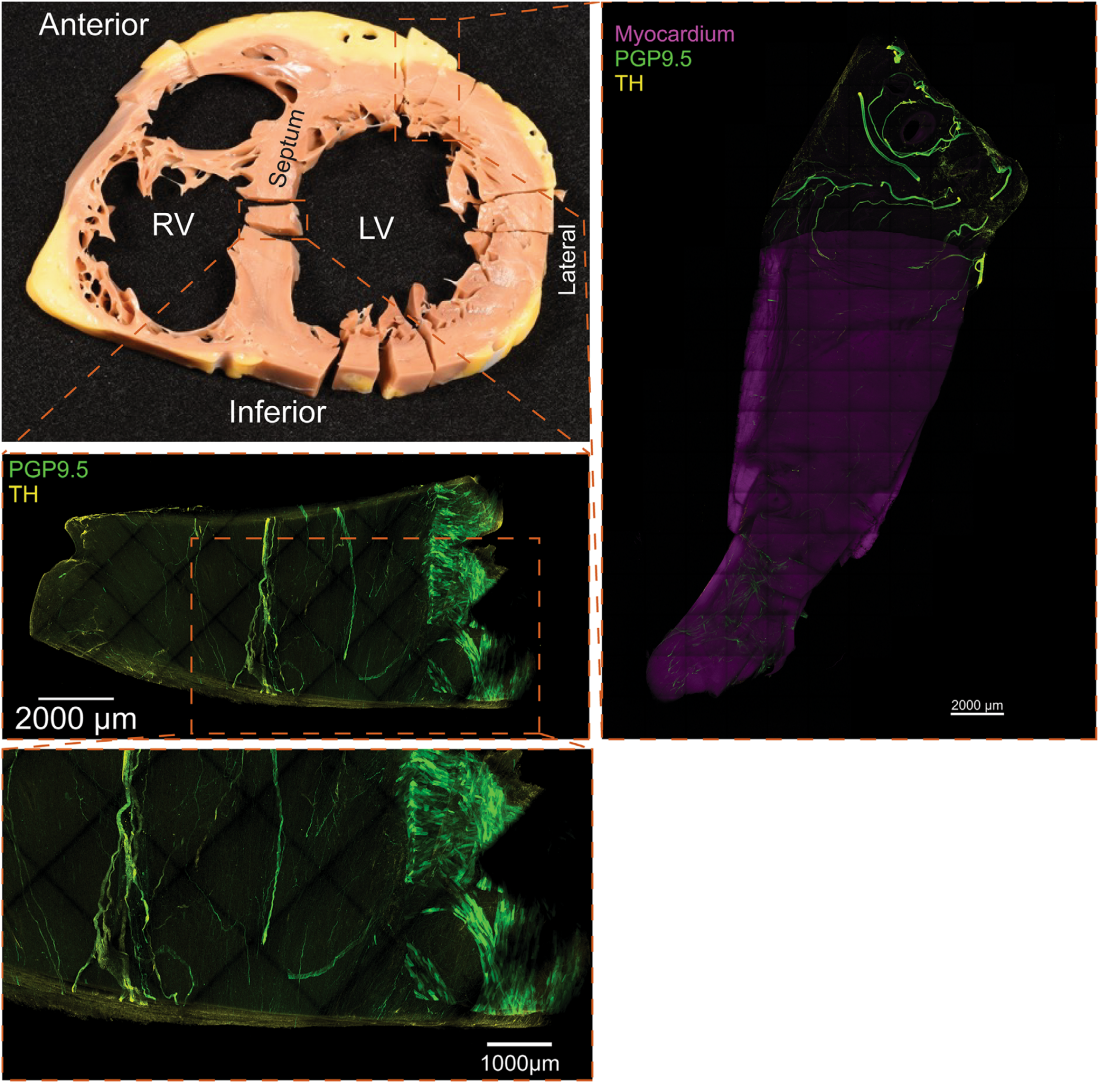
Short axis of left and right ventricles of a nonfailing human heart at the level of the mid‐chamber. Right panel, confocal image of iDISCO+‐cleared mid‐anterior left ventricular wall demonstrating course of PGP9.5+ (green) and TH+ (yellow) fibers in the epicardial fat and around a coronary vessel before penetrating into the myocardium (magenta). Note the PGP9.5+ myocytes at the endocardial aspect of the section. Middle left panel, confocal image of iDISCO+‐cleared mid‐interventricular septum demonstrating course of PGP9.5+ (green) and TH+ (yellow) fibers down the center of the septum. Lower left panel, note the significant PGP9.5+ staining of the endocardial aspect of the septum in the His‐Purkinje system.

**FIGURE 9:**
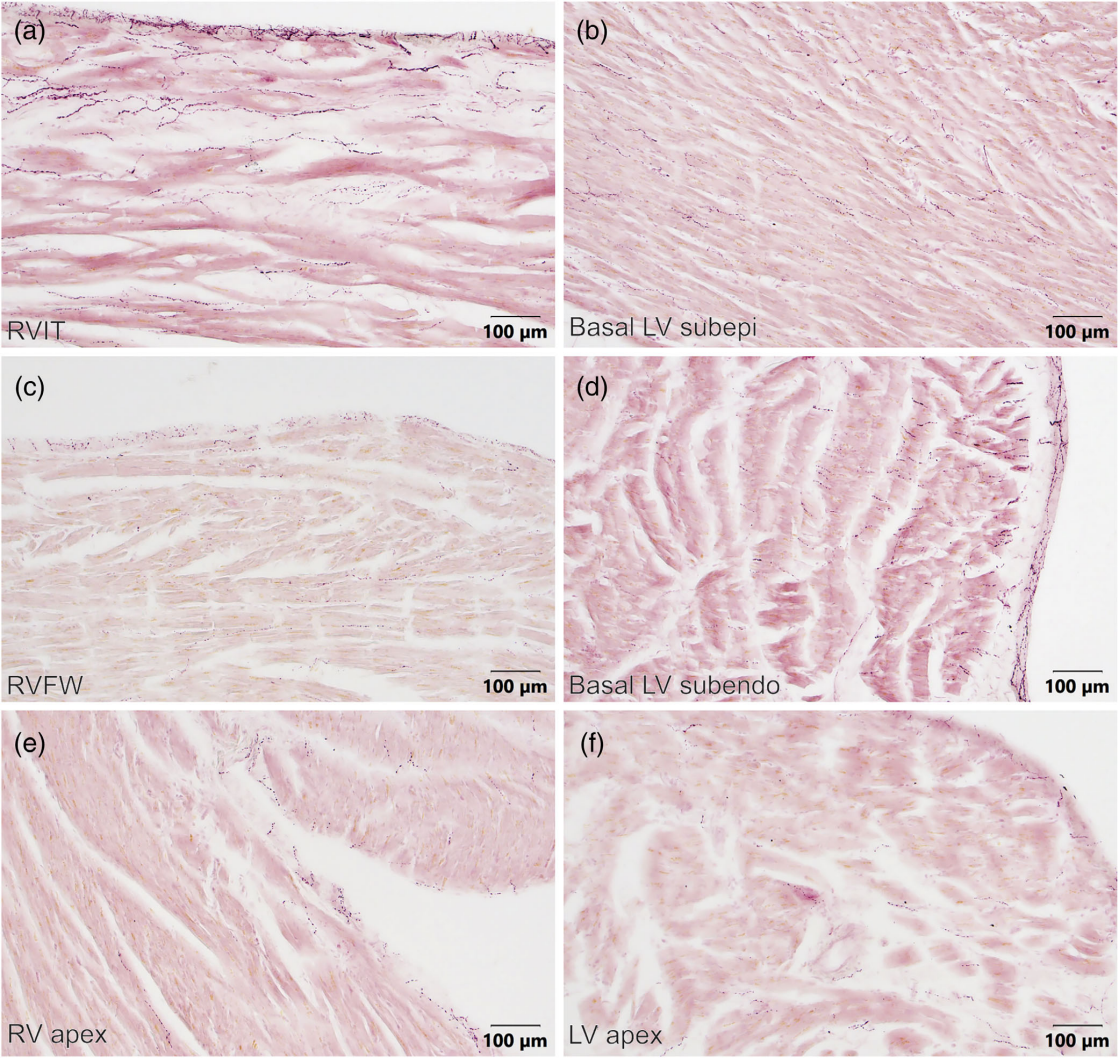
Cholinergic nerve fibers are present in right and left ventricular regions from the base of the heart to the apex, but at a much lower density than noradrenergic nerve fibers. Representative photomicrographs of sections from D1 heart (control) stained for VAChT. (a) Right ventricular inflow tract (RVIT) endocardium and subendocardium. (b) Basal left ventricular (LV) free wall subepicardium. (c) Right ventricular free wall (RVFW) endocardium and subendocardium. (d) Basal LV free wall endocardium and subendocardium. (e) RV apex endocardium and subendocardium. (f) LV apex endocardium and subendocardium.

Noradrenergic nerve fibers were distributed in the right and left ventricles from the base to the apex (Figure 7 and Table 4). Both the LV free wall and the septum were innervated by TH+ nerve fibers. While PGP9.5+ nerve fibers had a similar distribution, they were often more abundant, especially in the midwall region of transmural sections (Figures 8 and S4 and Table 4). Cholinergic nerve fibers were also detected in the RVs and LVs from the base to the apex (Figure 9 and Table 4), but they were far less abundant than noradrenergic fibers. Ventricular cholinergic nerve fibers were most concentrated in the endocardium and subendocardium. Some transmural presence of cholinergic fibers occurred at basal LV levels but rarely at more apical regions.

**FIGURE 10:**
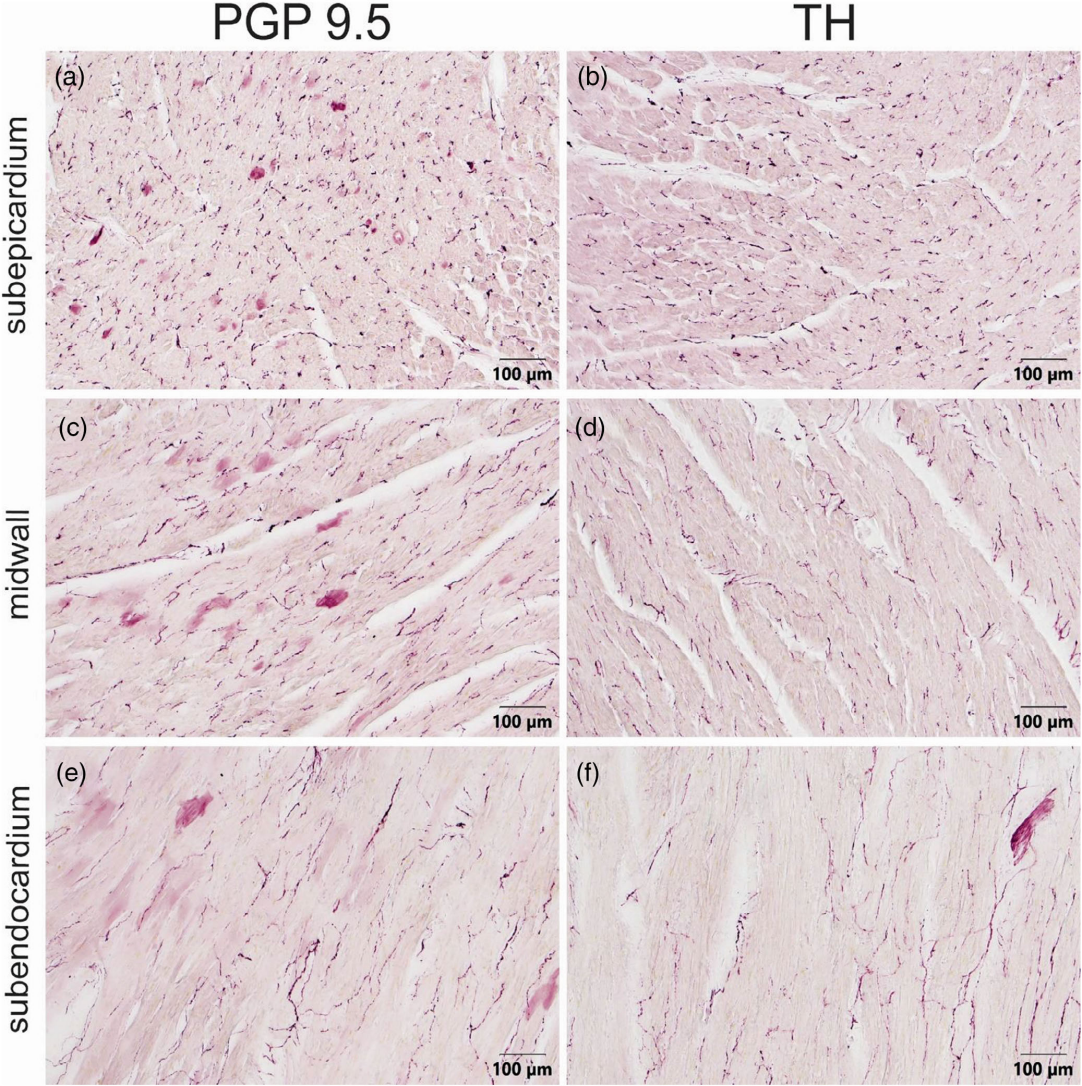
Abundant TH+ and PGP9.5+ nerve fibers occurred at comparable densities across the lateral mid‐LV free wall of the control heart from donor D8. Note that a few cardiomyocytes also showed light to moderate staining for PGP9.5 in this donor.

Given the variable LV innervation observed within and between donors, we evaluated LV free wall and apex samples from three additional control donors (D7–D9 in Table 1). All these samples had more abundant noradrenergic innervation, and it was more uniform across the wall Figure 10b,d,f and Table 4). Even so, the density of noradrenergic innervation still varied between these donors, with samples from D9 clearly being the lowest. Cholinergic nerve fiber density was slightly higher in these LV samples except for those from the donor with the lowest TH innervation (i.e., D9; Table 4). Cholinergic innervation at the LV apex was likewise higher in samples from two of these donors, with such nerves detected at a low density throughout the muscle and around blood vessels (Figure S5), including the microvasculature (Figure S5c,d).

While PGP9.5 is recognized as a pan‐neuronal marker, previous studies noted its expression by cardiomyocytes, especially in the conducting system (Airey et al., 2004; El Sharaby et al., 2001; Gill et al., 2007; Mueller et al., 2003). We detected intense staining of myocytes in the ventricular conducting system of control hearts (Figures 8 and S6a), but it was also expressed at variable intensity in several atrial myocytes in control hearts (Figure S6c,e). PGP9.5 was rarely or sparsely present in intramural ventricular myocytes of control hearts (Figures 10, S4, and S7), except at the apex (Figure S6b). We also observed PGP9.5 staining of presumed fibroblasts in the atrial epicardium of control hearts (Figure S6f).

Cholinergic and noradrenergic innervation of coronary vessels was detected throughout the ventricles (Figures 7d and S5b–d). Generally, the presence or absence of coronary innervation corresponded to the presence of muscle innervation by the same nerve type.

### 3.6 Ventricular innervation in cardiomyopathy

Innervation of the ventricles, most notably the LV, was clearly altered in all hearts with CM. It should be noted that the age and etiology of pathology varied for these hearts, as did the extent of pathology, but they all had substantial reductions of ejection fraction (EF; Table 1). While specific neural changes will be noted for each heart, the dominant form of remodeling was regional or heterogeneous nerve fiber loss. Such changes made quantification of regional innervation densities difficult for LV regions, and meaningful quantification was impossible at some sites (Table 4). Heterogeneous innervation patterns are reflected in higher SD values for nerve fiber densities. The extent of neuronal remodeling generally tracked with the degree of cardiac pathology. For the hearts with NICM, gross and microscopic ventricular pathology was much less in CM1 compared to CM2. LV damage and neuronal remodeling were most extensive for CM2 (NICM) and CM5 (ICM).

TH+ and PGP9.5+ innervation of RV regions was maintained in the CM hearts except at the apex where marked heterogeneity occurred (Table 4). Cholinergic nerve fibers were absent at the RV apex of all CM hearts and from other RV regions for CM2 and CM5. The same pattern for cholinergic nerve fiber loss occurred in LV regions with some preservation in CM1 and depletion of these nerve fibers in CM2 and CM3 (Table 4).

PGP9.5+ myocytes were rare or absent in proximal RV regions of CM1, but numbers increased at the RV apex as seen in controls. PGP9.5+ myocytes increased slightly from CM2 to CM5, but a sharper increase occurred in the RA appendage (Figure S8).

**FIGURE 11:**
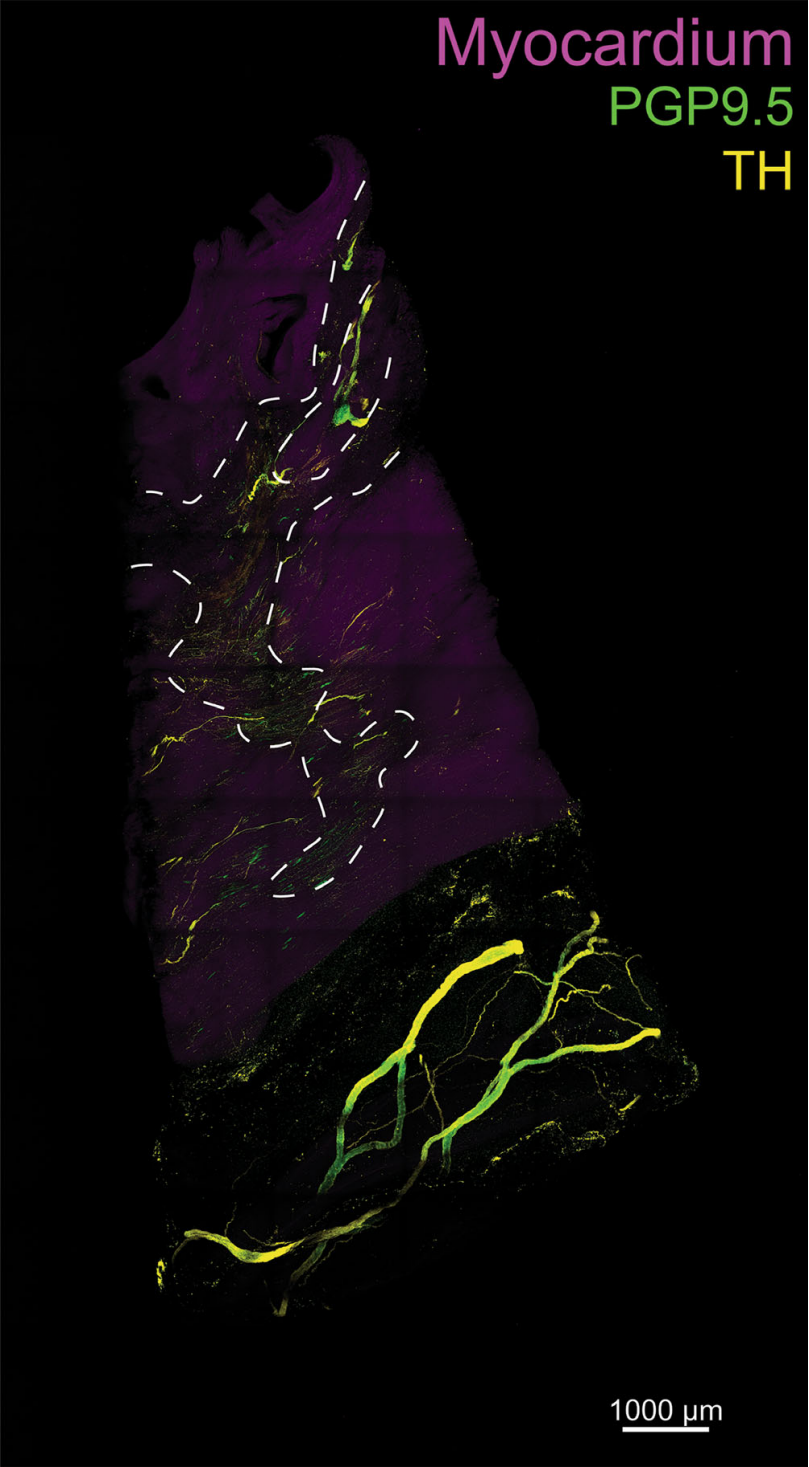
Confocal image of iDISCO+‐cleared mid‐lateral left ventricle from CM2. Note the large PGP9.5+ (green) and TH+ (yellow) fibers in the epicardial fat. Patchy infarcts (dashed white lines) were noted in the myocardium (magenta) with surrounding PGP9.5+ myocytes.

**FIGURE 12:**
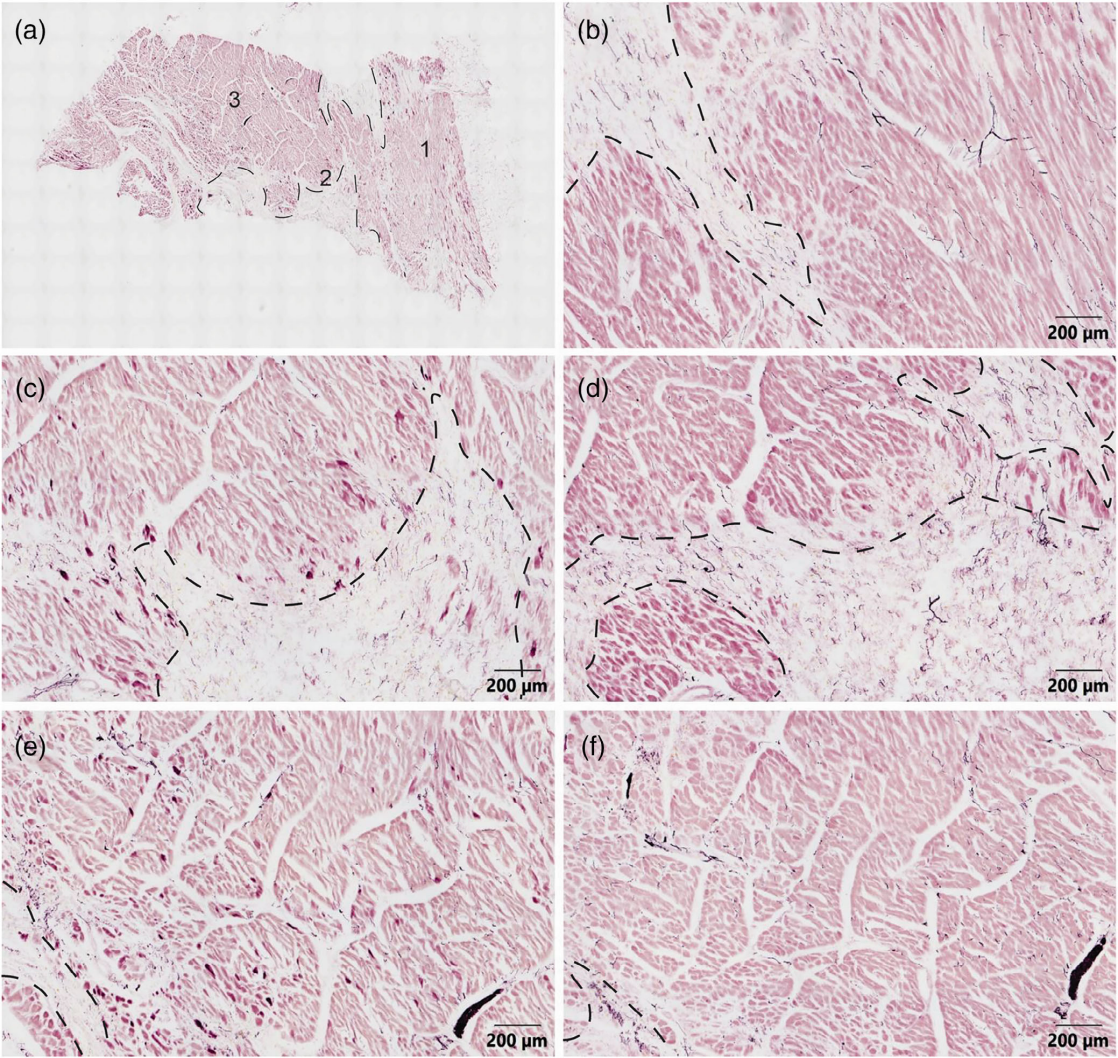
Neuronal and cardiac remodeling in the mid‐LV inferior wall of CM2 (NICM). (a) Full scan of section stained for PGP9.5 for orientation and to show regional labeling of nerve fibers and myocytes in region containing a subacute infarct, denoted by relative absence of cardiomyocytes (dashed black line). Higher resolution scans of this section and corresponding TH section are available as Figures S9 and S10, respectively. (b) Image of TH+ nerve fibers at region 1 in panel (a). Note the juxtaposition of innervated and non‐innervated myocardium. (c, d) PGP9.5 (c) and TH (d) staining in region indicated by 2 in panel (a). This region has granulation tissue at lower right corner, sparse innervation of myocardium, and upregulation of PGP9.5 by myocytes. (e, f) Images of PGP9.5 (e) and TH (f) staining at the subendocardial/midwall region indicated by 3 in panel (a). Note regional loss of innervation and upregulation of PGP9.5 by scattered myocytes near areas of subacute infarct (dashed black line).

CM1 and CM2 had minimal coronary stenoses, and CM2 had mild coronary artery disease. However, both had dilated CM and microscopic evidence of LV injury. Pathology noted multifocal, subendocardial ischemic injury for CM1, and CM2 had patchy subacute subendocardial infarcts and granulation tissue, which forms during the healing process following infarction, in the lateral mid‐LV (Figures 11, 12, S9, and S10). CM5, which was from an older patient with a history of coronary artery disease and multiple stents, the anatomic pathologist noted patchy white discoloration of the LV wall and septum, healed transmural infarct, and apical aneurysm.

**FIGURE 13:**
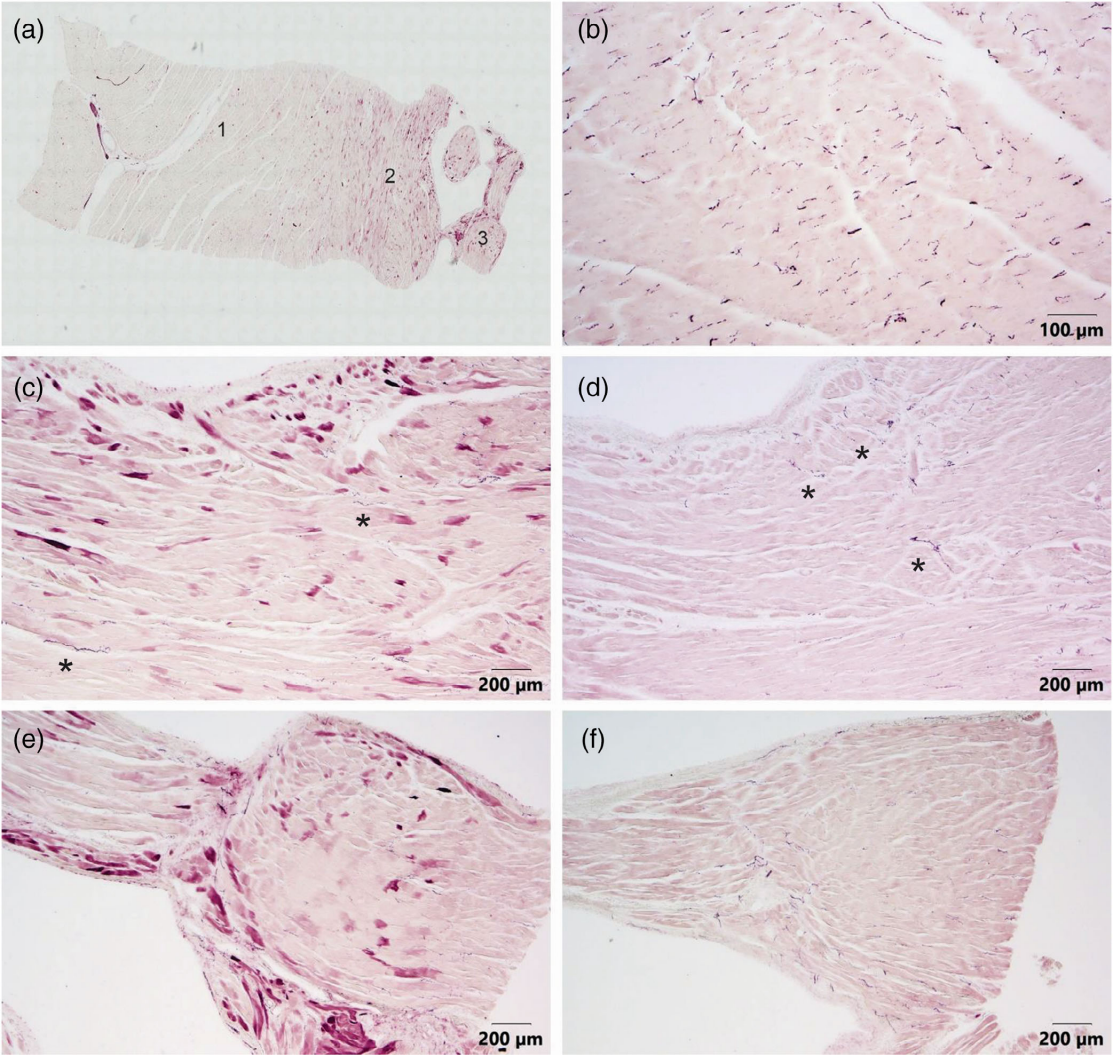
Neuronal and cardiac remodeling in the mid‐LV anterior wall of CM1 (NICM). (a) Full scan of section stained for PGP9.5 for orientation and to show regional staining of nerve fibers and myocytes. Higher resolution scans of this section and corresponding TH section are available as Figures S11 and S12, respectively. (b) Image of TH+ nerve fibers at the midwall region indicated by 1 in panel (a). Density of noradrenergic innervation appears normal here, in other midwall regions, and the subepicardium (left of panel (a)). (c, d) PGP9.5 and TH staining in the region indicated by 2 in panel (a). This region had a marked decrease in innervation and several myocytes that stained for PGP9.5. Scattered TH+ nerve fibers were present at only a few sites (asterisks). (e, f) TH and PGP9.5 staining in the region indicated by 3 in panel (a). There is prominent staining of myocytes for PGP9.5 (e) and marked regional loss of TH+ nerve fibers (f).

**FIGURE 14:**
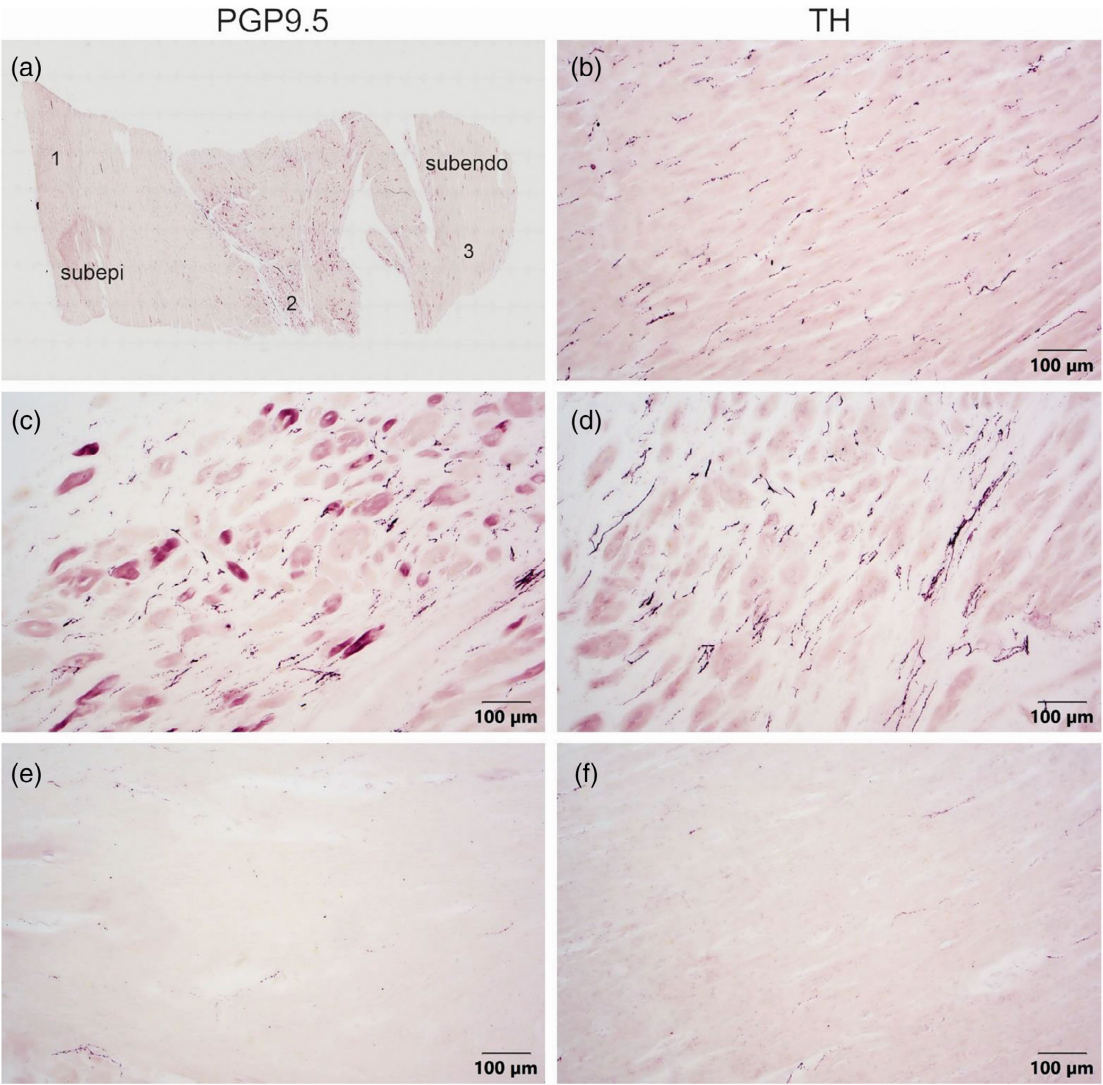
Neuronal and cardiac remodeling in the mid‐LV lateral wall of CM1 (NICM). (a) Full scan of section stained for PGP9.5 for orientation and to show regional staining of nerve fibers and myocytes. Higher resolution scans of this section and corresponding TH section are available as Figures S13 and S14, respectively. (b) Image of TH+ nerve fibers at the subepicardial region indicated by 1 in panel (a). Density of noradrenergic innervation appears normal here, throughout the subepicardium, and well into the midwall. (c, d) PGP9.5 and TH staining in region indicated by 2 of panel (a). This region had fibrotic changes, upregulation of PGP9.5 by myocytes (c), and prominent innervation by PGP9.5+ and TH+ nerve fibers (c and d, respectively). (e, f) Images of PGP9.5 and TH staining at the subendocardial region indicated by 3 in panel (a). Nerve fiber loss is prominent in this region.

LV samples from CM1 had the best preservation of TH+ and PGP9.5+ nerve fibers, but abnormal innervation was evident at several locations. For example, marked loss of these nerves occurred in large regions of the anterior mid‐LV subendocardium (Figures 13a,c–f, S11, and S12). Several myocytes in the same region and extending into the midwall were also stained for PGP9.5, whereas subepicardial myocytes remained negative for PGP9.5 (Figure S11). Noradrenergic innervation appeared normal in midwall (Figures 13b and S12) and subepicardium, as did the presence of PGP9.5+ nerve fibers. Areas with normal innervation and nerve loss often abutted in this sample and others. A similar pattern of nerve loss and PGP9.5+ myocytes occurred in other LV regions (Figure S13). Additionally, we observed an area of fibrosis in the lateral mid‐LV subendocardium (Figure 14). This region received prominent innervation and contained several PGP9.5+ myocytes (Figures 14, S13, and S14).

**FIGURE 15:**
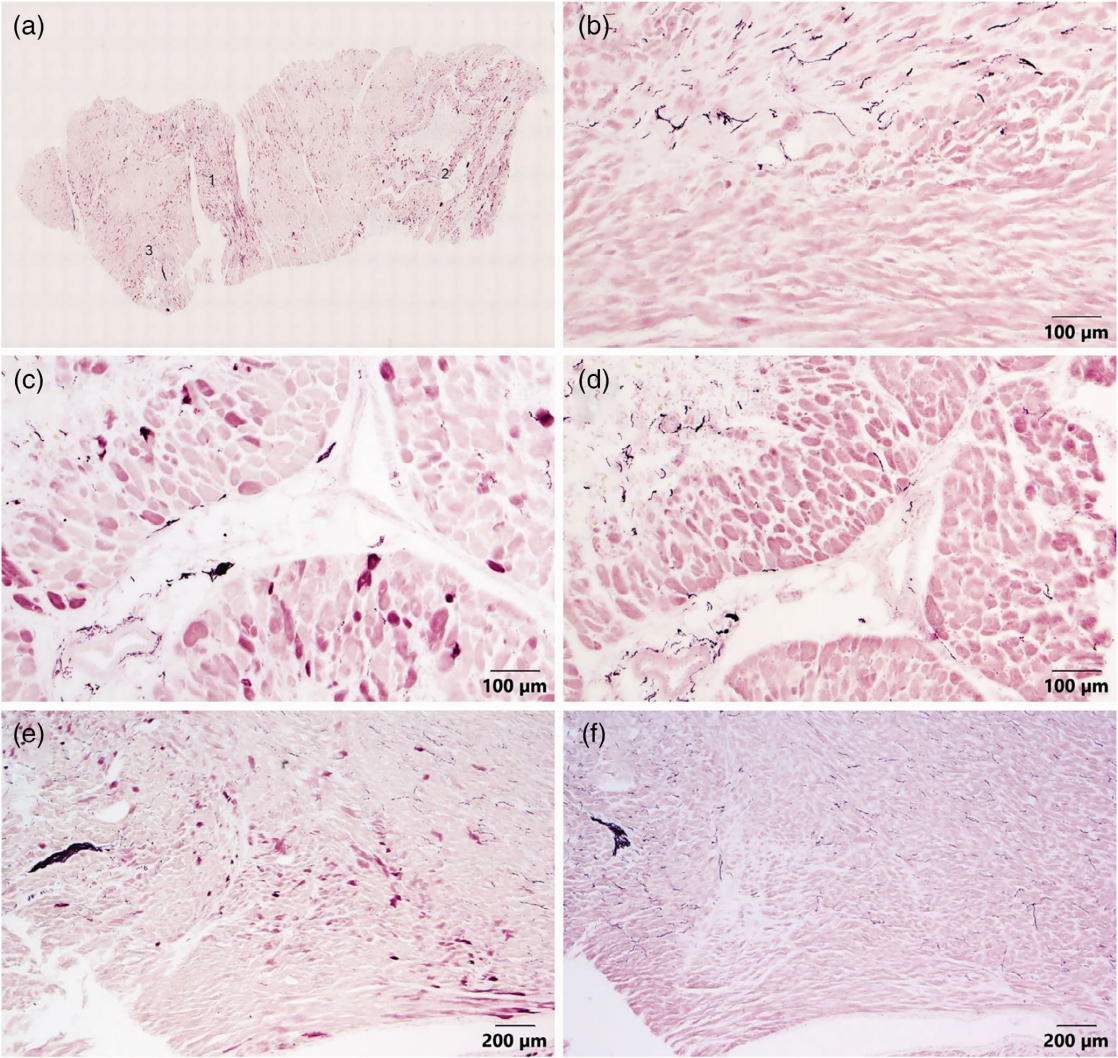
Neuronal and cardiac remodeling in the mid‐LV inferior wall of CM2 (NICM). (a) Full scan of section stained for PGP9.5 for orientation. Higher resolution scans of this section and corresponding TH section are available as Figures S15 and S16, respectively. (b) Image of TH+ nerve fibers at region 1 in panel (a). Note the juxtaposition of innervated and non‐innervated myocardium. (c, d) PGP9.5 (c) and TH (d) staining in the region indicated by 2 in panel (a). This region has granulation tissue in the upper left corner, sparse innervation of myocardium, and upregulation of PGP9.5 by myocytes. (e, f) Images of PGP9.5 (e) and TH (f) staining at the subendocardial region indicated by 3 in panel (a). Note regional loss of innervation and upregulation of PGP9.5 by scattered myocytes.

The LV free wall of CM2 had more widespread areas of spotty TH+/PGP9.5 nerve fiber loss and scattered PGP9.5+ myocytes throughout the transmural sections (Figures 12, 15, S9, S10, S15, and S16). Numerous infarcts were evident in agranulation tissue, which was often surrounded by PGP9.5+ myocytes (Figures 11, 12, 15, S9, S10, S15, and S16). Some of these infarcts had prominent noradrenergic innervation, but nerve fibers were less abundant in bordering myocardium. Of note, many border zone muscle regions seemed to have fewer noradrenergic nerve fibers than normal myocardium.

**FIGURE 16:**
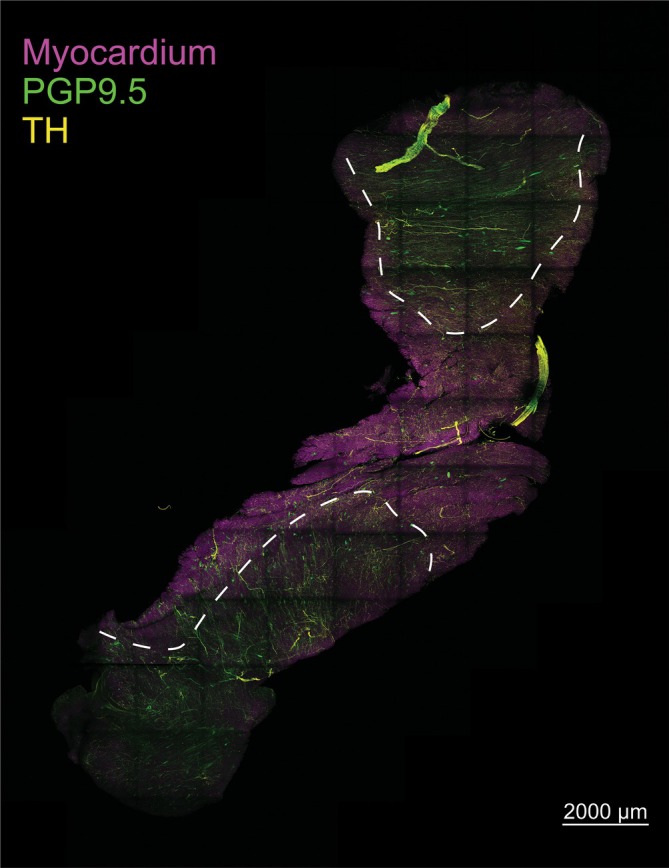
Confocal image of iDISCO+‐cleared basal‐lateral left ventricle from CM5. Noted the reduced myocardium (magenta) signifying areas of infarct and fibrosis (dashed white lines). Increased PGP9.5+ (green) cardiomyocytes were noted in the regions of infarct and endocardial aspect of the specimen, in addition to the PGP9.5+ fibers (green). Large TH+ (yellow) nerve bundles are also shown.

**FIGURE 17:**
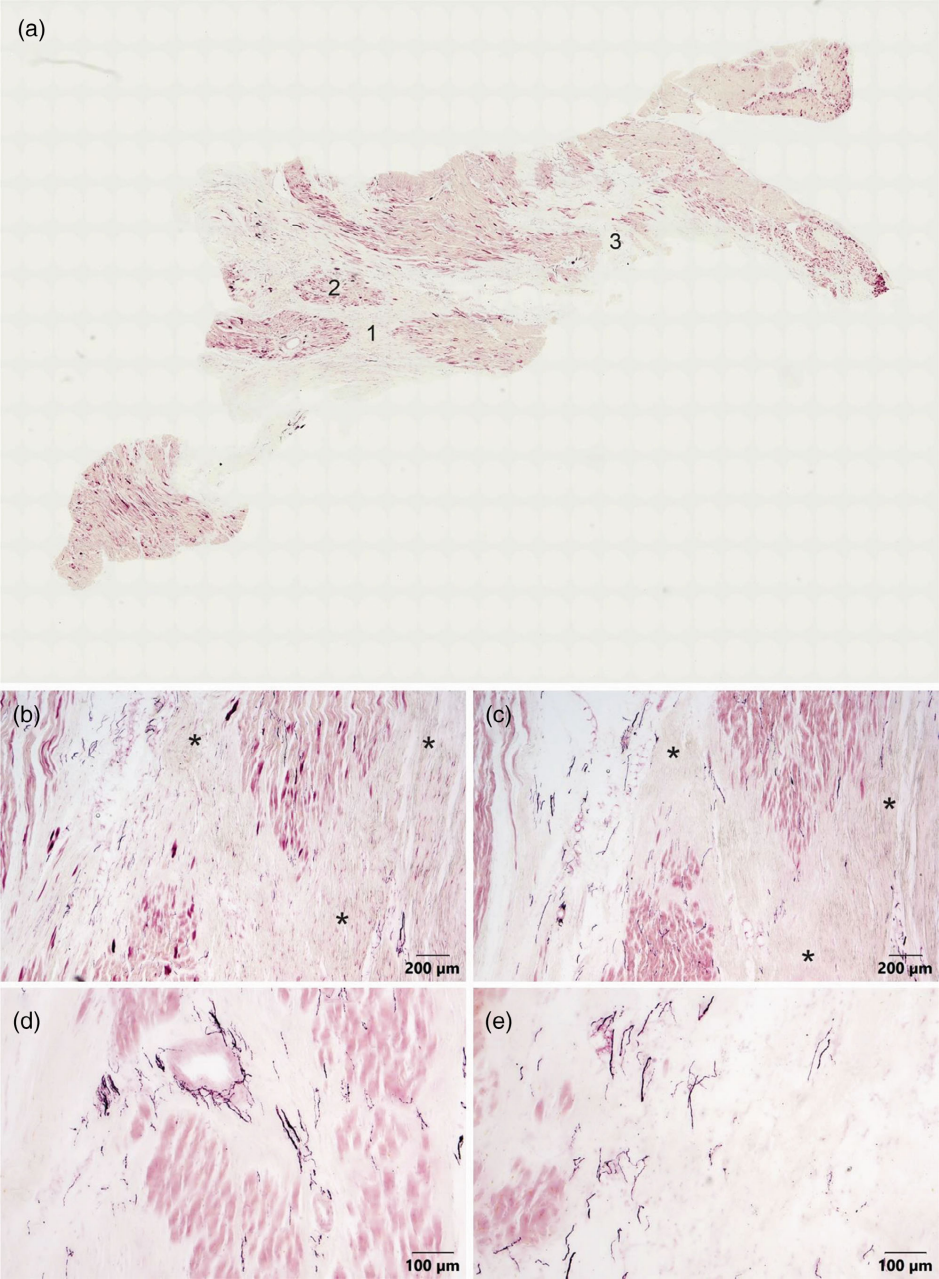
Neuronal and cardiac remodeling in the mid‐LV septum of donor CM5 (ICM). (a) Full scan of section stained for PGP9.5 for orientation. Higher resolution scans of this section and corresponding TH section are available as Figures S17 and S18, respectively. (b, c) PGP9.5 and TH staining in region indicated by 1 in panel (a). This is a border zone region of intact muscle with healed scar (asterisks). Note PGP9.5+ myocytes (b), variable noradrenergic innervation of border zone (c), and sparsity of TH+ nerve fibers in scar regions. (d) TH staining in region indicated by 2 in panel (a). Note prominent innervation of subendocardial arteriole and variable innervation of adjacent myocardium. (e) Noradrenergic innervation of granulation tissue indicated by 3 in panel (a).

Donor CM5 had the most extensive pathology of LV regions, including the septum, and this was reflected in widespread heterogeneous loss of TH+/PGP9.5+ nerve fibers and upregulation of PGP9.5 by cardiomyocytes (Figure 16). Much of the septum contained a healed infarct, which, in contrast to granulation tissue, lacked noradrenergic nerve fibers (Figure 17). Surviving septal muscle had heterogeneous innervation with scattered PGP9.5+ myocytes throughout (Figures 17, S17, and S18).

### 3.7 Presence of lipofuscin in cardiomyocytes

During this investigation, we noted the presence of lipofuscin in cardiomyocytes, which is not surprising since accumulation of this pigment is considered an aging phenomenon (De Meyer et al., 2010; Kakimoto et al., 2019; Li et al., 2021). However, it is noteworthy that lipofuscin was most evident in LV samples, and its occurrence was not tightly associated with age. For example, minimal lipofuscin was detected in LV free wall from the youngest patients with NICM (CM1 and CM2) and the 52‐year‐old control donor (D8), but lipofuscin+ myocytes were very prominent in the 49‐year‐old control donor (D2; Figure S19).

## 4 DISCUSSION

Although critical autonomic neural input to the heart is mediated by an integrated network that interfaces with the myocardium directly through sympathetic and parasympathetic nerves, surprisingly little is known about the precise distribution of these nerves within the complex and asymmetric landscape of the human heart. By immunohistochemical analysis of tissue sections from numerous regional samples dissected from donor human hearts, we have generated a comprehensive picture of the distribution of cholinergic and noradrenergic nerve fibers in the heart. Specifically, we have shown: (1) Both types were ubiquitous throughout the atria of control hearts and occurred at similar densities, except at the SAN, where cholinergic nerve fibers were more abundant. (2) In the ventricles, cholinergic and noradrenergic nerve fibers were detected throughout, but cholinergic fibers were far less abundant, especially in the septum and LV. (3) Complex remodeling of autonomic innervation occurs in CM for atrial and ventricular regions. Such remodeling has major implications for aberrant neural control in the diseased heart.

While autonomic innervation of the heart has been the topic of intense investigation in several species, less information is available regarding the detailed regional innervation of human hearts. Previous work has focused primarily on epicardial structures of intact tissue (Pauza et al., 2000) or a limited number of regions in sectioned tissues (Chow et al., 2001; Crick et al., 1994; Kawano et al., 2003; Wharton et al., 1990). Furthermore, many previous studies used AChE histochemistry to identify intracardiac nerve fibers. This approach has the limitation that AChE is not a specific marker for cholinergic nerves (Koelle, 1955) and, therefore, may overestimate cholinergic innervation. While some experimental evidence supports the use of AChE (Coote, 2013), we chose to circumvent this problem by targeting the specific cholinergic protein, VAChT (Prado et al., 2002; Usdin et al., 1995) by using immunohistochemistry (Aras et al., 2022; Downs et al., 2016; Hanna et al., 2021). Thus, parallel immunostaining for VAChT and the specific noradrenergic marker TH has provided a precise and detailed picture showing the relative abundance of noradrenergic and cholinergic innervation throughout the heart.

### 4.1 Normal atrial innervation

Cholinergic and noradrenergic nerve fibers were detected throughout control atria and occurred at comparable densities at most sites. This pattern would facilitate balanced neural regulation of dromotropic and inotropic functions. However, cholinergic nerve fibers were more abundant than noradrenergic fibers at the SAN, which matches our recent finding for porcine SAN (Hanna et al., 2021). Our findings for LA regions differ from some previous studies, which found that cholinergic nerve fibers were less abundant than noradrenergic fibers (Depes et al., 2022; Nguyen et al., 2009). This discrepancy might be due to their use of a ChAT antibody. Although ChAT is a specific cholinergic marker, commercial antibodies to this protein do not label peripheral ChAT as well as brain ChAT and might underestimate cholinergic nerve fiber density. Overall, our findings provide a structural foundation for chronotropic, dromotropic, and inotropic effects of sympathetic and parasympathetic nerves in human atria.

### 4.2 Normal ventricular innervation

Our findings for innervation of the ventricles present a more complex picture, but the evidence clearly supports a dominant role for noradrenergic innervation throughout the ventricular myocardium. While cholinergic nerve fibers were always less abundant than noradrenergic nerve fibers, sparse to low densities were detected at multiple transmural locations in RV and LV sections from most donors. Although our number of donors was limited for RV regions, similar results were found for most RV samples from NICM hearts. While significant cholinergic innervation of the RV outflow tract was detected in our recent study with a larger donor population (Aras et al., 2022), noradrenergic nerve fibers were more abundant. Noradrenergic innervation generally occurred at moderate to high density across the ventricular walls from the base to the apex in the present study. Both the distribution and abundance of PGP9.5+ nerve fibers paralleled that of TH+ nerve fibers, although there were some discrepancies. The latter might be attributed in part to sampling differences, since separate sections were labeled for PGP9.5 and TH. A further consideration is that PGP9.5 would also label sensory nerve fibers (Wharton et al., 1990). It is also possible that expression of PGP9.5 and TH are differentially regulated, since these enzymes have different functions within the nerve fibers (i.e., deubiquitination vs. tyrosine hydroxylation, respectively). Overall, our study provides structural evidence to support a dominant role of noradrenergic innervation in control of ventricular contractility and excitability. This view is supported by evidence that noradrenergic nerve fibers dominate in nerve bundles that supply the human ventricles (Petraitiene et al., 2014).

While direct cholinergic neuroregulation of RV function is likely based on our anatomical findings, especially in areas such as the RV inflow and outflow tracts, the extent to which sparse cholinergic innervation directly affects LV function is unclear. One possibility is that neurally released ACh might produce broad regional effects by diffusion (i.e., volume transmission). Naturally, this mechanism would be supported by the lack of ACh reuptake by cholinergic nerves. Another novel possibility is that cholinergic effects might be amplified by the synthesis and release of ACh by cardiomyocytes (Habecker et al., 2016; Kakinuma et al., 2009; Oikawa et al., 2021). Although this mechanism has been documented in rodent studies, similar work has not been reported for human cardiomyocytes (Xie et al., 2024).

### 4.3 Changes in neural network in cardiomyopathy

Neuronal remodeling is a major contributor to adverse events in patients with cardiovascular disease and has been the topic of numerous preclinical and clinical studies. However, much of our clinical knowledge about remodeling comes from imaging studies for sympathetic nerves in patients (Bengel, 2005; Bravo et al., 2015; Fallavollita et al., 2014; Fallavollita et al., 2017; Seo et al., 2022; Thackeray & Bengel, 2013), and limited data is available at the microscopic and cellular levels for the human heart (Cao, Chen, et al., 2000; Cao, Fishbein, et al., 2000; Chen et al., 2001; Oki et al., 1994; Park et al., 2002). In this study, we found prominent changes in the innervation of atrial and LV samples from donors with NICM and ICM. Remodeling of noradrenergic nerves appeared as increases or decreases in nerve density, while that of cholinergic nerves manifests as decreases only.

Side‐dependent changes in atrial innervation were prominent in the CM hearts, with a substantial increase in noradrenergic nerve density appearing in the RA appendage of pooled NICM and ICM samples, and total denervation of the ICM LA appendage. Our findings are consistent with a previous study that found noradrenergic hyperinnervation of the RA appendage in patients with rheumatic heart disease (Li et al., 2013). In that study, the abundance of nerve fibers staining for growth‐associated protein 43, like that for TH, was also increased, suggesting that nerve growth occurred. Cholinergic innervation was not altered in this study, also in accord with our results. These authors suggested a link between noradrenergic hyperinnervation and atrial fibrillation in their patients. This concept is supported by earlier work that showed sympathetic hyperinnervation of the RA appendage and elevated tissue norepinephrine levels in patients with persistent atrial fibrillation (Gould et al., 2008). Surprisingly, the same parameters were not elevated in the LA appendage. Our finding of increased density of noradrenergic nerves in the RA appendage might be due to upregulation of nerve growth factor (see below), while loss of all nerves from the LA appendage of the donor with ICM could have multiple contributing factors. Based on these findings, it is reasonable to predict that neuronal remodeling will be common throughout the atria in CM and that the resulting heterogeneity of innervation contributes to atrial arrhythmias.

Substantial remodeling of ventricular autonomic innervation was also associated with CM in our study. Cholinergic innervation of the LV myocardium, which was already low in control hearts, was largely eliminated in CM. This change is expected to further compromise cholinergic function, which is already reduced in cardiovascular disease, as evidenced by decreased heart rate variability (Thayer et al., 2010). Remodeling of ventricular noradrenergic innervation in CM was more complex and manifested as (1) heterogeneous nerve loss in regions of intact myocardium and (2) hyperinnervation of granulation tissue. Clinical evidence for the presence of compromised and heterogeneous sympathetic innervation of the ventricles in heart disease comes from numerous in vivo imaging studies using tracers such as ^123^I‐meta‐iodobenzylguanidine and ^11^C‐meta‐hydroxyephedrine (Thackeray & Bengel, 2013). These tracers are substrates for the norepinephrine transporter (NET), which is responsible for reuptake of NE after it is released from sympathetic nerves. However, since NET can be downregulated independently, decreased uptake of these tracers cannot discriminate between sympathetic dysfunction and nerve loss (Haider et al., 2010; Kristen et al., 2006; Thackeray & Bengel, 2013). Downregulation of NET can contribute to cardiac pathology, which ultimately leads to nerve loss (Habecker et al., 2016; Himura et al., 1993; Kaye et al., 2000; Kimura et al., 2010). Decreased TH staining in our cases of severe CM is an indicator of actual nerve loss. This observation agrees with a previous report of noradrenergic nerve loss in endocardial biopsies from patients with dilated CM (Oki et al., 1994). However, we identified heterogeneous nerve loss by evaluating full transmural sections. It is thought that such heterogeneity of noradrenergic innervation can contribute to ventricular arrhythmias (Fallavollita et al., 2014; Fukuda et al., 2015).

Previous studies have reported that hyperinnervation by noradrenergic nerves occurs at the border zone of infarcts (Cao, Fishbein, et al., 2000; Hasan et al., 2006), and these and other investigators proposed that this remodeling increases risk for ventricular arrhythmias and sudden cardiac death (Cao, Chen, et al., 2000; Cao, Fishbein, et al., 2000; Chen et al., 2001; Chen et al., 2007; Hasan et al., 2006; Ieda & Fukuda, 2009). In accordance with this work, we also found hyperinnervation at border zones in our CM samples, but this occurred only on the infarct side of the border zone in granulation tissue. In contrast, we usually found reduced innervation of myocardium at the border zone. We speculate that such juxtaposition of richly innervated granulation tissue and sparsely innervated myocardium would favor arrhythmogenesis, especially if adjacent muscle is denervated and develops b‐adrenergic receptor supersensitivity (Habecker et al., 2016). It should be noted that hyperinnervation of the border zone is transient in animal studies, occurring in early days after MI (Hasan et al., 2006). This is followed by loss of noradrenergic nerves as animals develop heart failure. Likewise, loss of noradrenergic nerves occurs with severe heart failure in humans and in preclinical models (Kaye et al., 2000; Kimura et al., 2010).

Although PGP9.5 is a well‐established pan‐neuronal marker, we also observed prominent labeling of some cardiomyocytes for this protein at discrete sites in control hearts and dramatically increased staining of LV myocytes for this marker in CM. Previous work has identified PGP9.5 as a marker for Purkinje cells (Airey et al., 2004; El Sharaby et al., 2001; Gill et al., 2007; Mueller et al., 2003), and we also observed staining of some epicardial fibroblasts for this marker. Expression of PGP9.5 by non‐neuronal cells is expected given its role as an essential regulator of ubiquitin signaling through its action to remove ubiquitin from proteins (Damgaard, 2021). Expression of PGP9.5 by Purkinje cells can serve as a marker for these elements of the conducting system, but it might also suggest a higher need for regulation of ubiquitin signaling in these cells. The expression of PGP9.5 by atrial myocytes could relate to their conductive function, but this is only speculation. However, in line with this concept, SAN myocytes did not express PGP9.5, but myocytes projecting from the node did. Regarding pathological conditions, several previous studies have reported increased PGP9.5 expression by ventricular myocytes in animal models of MI and in humans with CM (Bi et al., 2020; Drobysheva et al., 2013; Weekes et al., 2003; Wu et al., 2022). Immunohistochemical evidence presented in previous studies was limited but showed PGP9.5+ myocytes in the peri‐infarct region. We confirmed this finding and showed a much broader distribution of PGP9.5+ myocytes across the LV walls of all CM hearts and very prominent expression in the RA appendage of the ICM heart. It remains unknown if upregulation of PGP9.5 by cardiomyocytes has a beneficial or detrimental effect. However, recent work with cardiomyocyte selective deficiency of PGP9.5/UCHL1 suggests a protective effect of the enzyme in a post‐MI heart failure model (Wu et al., 2022). In this regard, upregulation of PGP9.5/UCHL1 would counter the hyperubiquitination of proteins that occurs in patients with dilated CM (Weekes et al., 2003).

## 5 LIMITATIONS

While the control donors in this study all appeared to have preserved pump function, most had evidence of acute or chronic pathology in their histories. We cannot eliminate the possibility that other diseases might affect the innervation of the heart. The age of donors varied over five decades, and it is possible that age might affect the innervation of the heart, possibly independent of disease. In fact, normal LV areas from the youngest donor with NICM had among the most robust innervation. Nevertheless, the range of control and CM donors was sufficient to improve our understanding of the normal cardiac innervation pattern and identify major neuronal remodeling that occurs in end‐stage heart failure.

## 6 CONCLUSIONS

This study provides extensive mapping of cholinergic and noradrenergic innervation of human atria and ventricles determined by immunohistochemical analysis of donor tissue. Control hearts had comparable abundance of both nerve types throughout the atria, except at the SAN where the density of cholinergic nerves was greater. Noradrenergic nerves were dominant throughout the ventricles, where cholinergic innervation was far less abundant. Major remodeling of atrial and ventricular innervation occurred in patients with end‐stage heart failure. Such changes are likely to be proarrhythmic and contribute to reduced pump function.

## AUTHOR CONTRIBUTIONS

**Peter Hanna:** Methodology; writing – review and editing; writing – original draft; funding acquisition; investigation; conceptualization; visualization; data curation. **Donald B. Hoover:** Conceptualization; investigation; funding acquisition; writing – original draft; writing – review and editing; visualization; methodology; supervision; formal analysis. **Logan G. Kirkland:** Data curation; investigation; formal analysis. **Elizabeth H. Smith:** Investigation; data curation; formal analysis. **Megan D. Poston:** Investigation; data curation; formal analysis. **Stanley G. Peirce:** Investigation; data curation; formal analysis. **Chloe G. Garbe:** Investigation; data curation; formal analysis. **Tasha K. Phillips:** Investigation; data curation. **Steven Cha:** Investigation; data curation. **Shumpei Mori:** Data curation; investigation. **Jaclyn A. Brennan:** Investigation; data curation. **John Andrew Armour:** Conceptualization. **Eric Rytkin:** Data curation; investigation. **Igor R. Efimov:** Conceptualization; funding acquisition; supervision. **Olujimi A. Ajijola:** Conceptualization; funding acquisition; writing – review and editing; supervision. **Jeffrey L. Ardell:** Conceptualization; funding acquisition; writing – review and editing; supervision. **Kalyanam Shivkumar:** Conceptualization; investigation; funding acquisition; writing – review and editing; supervision; resources.

## CONFLICT OF INTEREST STATEMENT

K.S. is a consultant for nFerence, Anumana, and Boston Scientific. O.A.A. has ownership interest in and stock ownership in nFerence and Neufera. All other authors have nothing to disclose.

## Supporting information


**Figure S1.** Slide scan of section stained for connexin 43 (Cx43) showing the localization of the sinoatrial node (SAN) and nearby contractile muscle of the right atrium (RA). Contractile muscle stains for Cx43 but nodal cells do not. Nodal artery is indicated by “a.” Scale bar is in lower left corner.

**Figure S2.** Comparison of cholinergic and noradrenergic innervation in left atrial (LA) muscle near a pulmonary vein. (a) Slide scan showing a full section containing LA and a portion of a pulmonary vein (PV). Number 1–3 indicated regions of LA where nerve fiber densities were measured. (b) Bar graph shows nerve fiber densities in sections from a single donor. Values are means ± SDs for at least nine images per data point. Note that VAChT+ cholinergic nerve fibers are more abundant than TH+ noradrenergic nerve fibers in the LA muscle, but TH+ nerve fibers outnumber VAChT+ nerve fibers vastly in the vein. (c, d) Representative images showing VAChT+ and TH+ nerve fibers, respectively, in region 2 of LA. (e, f) Representative images showing VAChT+ and TH+ nerve fibers, respectively, in the PV region indicated in panel (a).

**Figure S3.** Ganglia located in epicardial fat around the atrium/PV sample shown in Figure S1a–c: Staining for the cholinergic marker VAChT shows cholinergic cell bodies surrounded by cholinergic varicosities and nerve fibers. (a, b) Low and higher magnification images of the same ganglion. Note variable staining intensity of cell bodies. (c) Small ganglion embedded in nerve bundle at branching point. (d) Ganglion stained for the panneuronal marker PGP9.5. Note prominent staining of cell bodies and axons. Varicosities are not evident. (e, f) Two ganglia in sections stained for TH. Cell bodies exhibit mainly background staining but strong staining of nerve fibers is present. (e) TH+ nerves associated with this ganglion occur mainly in a bundle at the edge and few penetrate the ganglion. (f) Higher magnification of TH staining in this ganglion shows that varicose nerve fibers innervate several of the cell bodies, which are presumed to be cholinergic.

**Figure S4.** Transmural differences in staining for TH and PGP9.5 in the lateral mid‐LV free wall of control heart from D1. Note decreased staining for TH but not PGP9.5 at the midwall level.

**Figure S5.** A low density of VAChT+ cholinergic nerves occurred at many regions of LV apex in control donor heart D9. (a) Subepicardium. (b) VAChT+ nerves surround an artery and innervate muscle at a midwall region. (c) VAChT+ nerve fibers traverse connective tissue and surround a micro vessel above muscle at top right. (d) Higher magnification image of same field as panel (c) showing cholinergic innervation of micro vessel. (e) Nerve bundle at the lower left contains several VAChT+ nerve fibers. (f) Single cholinergic neuron observed in the heart was the only neuron associated ventricular tissue among all the hearts evaluated.

**Figure S6.** Expression of PGP9.5 by cardiomyocytes occurred at specific sites in control hearts. (a) Purkinje cells and nerve fibers in the mid‐LV septum had intense staining for PGP9.5 that was noted in sections and tissue cleared specimens as the endocardial aspect of the mid‐left ventricular anterior wall. (b) Several myocytes at the LV apex stained strongly for PGP9.5, but such staining was not detected at other ventricular sites beyond the conducting system. (c) Low magnification image of the SA node (d) and surrounding right atrium (e) showing staining pattern for PGP9.5. (d) Higher magnification of the SA node region (d in panel (c)) where PGP9.5 is localized only to nerve fibers. (e) Higher magnification of the surrounding right atrium (e in panel (c)) where PGP9.5 is localized to cardiomyocytes and nerves. (f) PGP9.5 staining of fibroblasts (arrows) in subepicardium near the SAN. All these images were from control donor D1.

**Figure S7.** Montage image showing PGP9.5 stain of a transmural section of anterior mid‐LV from donor D1. Montage was created by stitching multiple 10× images using an Olympus BX41 microscope equipped with an Olympus DP74 digital camera and cellSens Dimension software.

**Figure S8.** Variable staining of PGP9.5+ myocytes were observed in the right atrium of normal and cardiomyopathy hearts. (a) Image showing typical myocyte staining pattern within the RA of D1. (b) Limited quantities of PGP9.5+ myocytes were observed in D2. (c) Representative image showing lack of PGP9.5+ myocytes in donor CM1. (d) Extensive quantities of PGP9.5+ myocytes were observed in the RA in donor CM5.

**Figure S9.** Montage image showing PGP9.5 stain of a large section of lateral mid‐LV from donor CM2 (NICM).

**Figure S10.** Montage image showing TH stain of a transmural section of lateral mid‐LV from donor CM2 (NICM).

**Figure S11.** Montage image showing PGP9.5 stain of a transmural section of anterior mid‐LV from donor CM1 (NICM). Montage was created by stitching multiple 10× images using an Olympus BX41 microscope equipped with an Olympus DP74 digital camera and cellSens Dimension software.

**Figure S12.** Montage image showing TH stain of a transmural section of anterior mid‐LV from donor CM1 (NICM).

**Figure S13.** Montage image showing PGP9.5 stain of a transmural section of lateral mid‐LV from donor CM1 (NICM).

**Figure S14.** Montage image showing TH stain of a transmural section of lateral mid‐LV from donor CM1 (NICM). TH+ nerves were observed across the entirety of the tissue, from subendocardium to subepicardium. Note the heterogeneity of TH+ nerve density across the tissue wherein large areas of tissue have little‐to‐no innervation while others are densely innervated. Montage was created by stitching multiple 10× images using an Olympus BX41 microscope equipped with an Olympus DP74 digital camera and cellSens Dimension software.

**Figure S15.** Montage image showing PGP9.5 stain of a transmural section of posterior mid‐LV from donor CM2 (NICM).

**Figure S16.** Montage image showing TH stain of a transmural section of posterior mid‐LV from donor CM2 (NICM).

**Figure S17.** Montage image showing PGP9.5 stain of a transmural section of mid septum from donor CM5 (ICM).

**Figure S18.** Montage image showing TH stain of a transmural section of mid septum from donor CM5 (ICM).

**Figure S19.** Lipofuscin deposits were found in varying quantities in the mid‐LV of two donors (D9 and D2). (a) Image of subepicardium from D9 showing limited amounts of lipofuscin (orange dots) within cardiomyocytes. (b) Image of subepicardium showing lipofuscin deposits (arrow) in D2. (c) Image of midwall PGP9.5 stain showing lipofuscin deposits in D9. Note the increased amount of lipofuscin staining in panel (c) compared to panels (a) or (e). (d) Lipofuscin deposits (arrow) within the midwall of D2. (e) PGP9.5 staining of subendocardium in D9 shows limited amount of intramuscular lipofuscin. (f) PGP9.5 staining shows large lipofuscin deposits (arrow) in subendocardium in D2.
