# Supplementary figures and images for: Noradrenergic and cholinergic innervation of the normal human heart and changes associated with cardiomyopathy
Source: Anat Rec (Hoboken). 2025 May 14;309(2):417–50. doi: 10.1002/ar.25686 (PMC12353864; doi:10.1002/ar.25686)

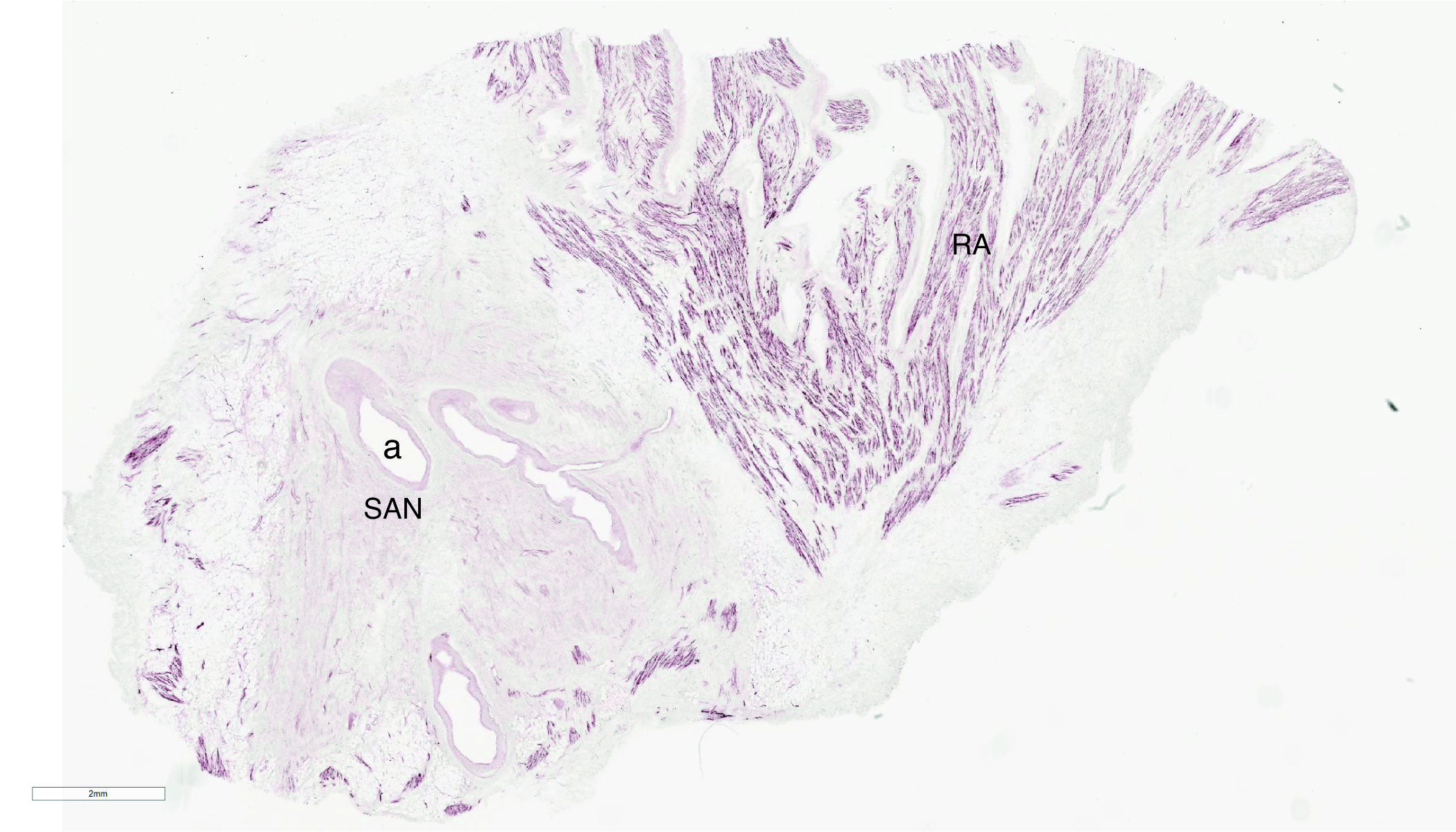

Supplement: Supplementary file 1 — Figure S1. Slide scan of section stained for connexin 43 (Cx43) showing the localization of the sinoatrial node (SAN) and nearby contractile muscle of the right atrium (RA). Contractile muscle stains for Cx43 but nodal cells do not. Nodal artery is indicated by “a.” Scale bar is in lower left corner. [file AR-309-417-s003.jpg]

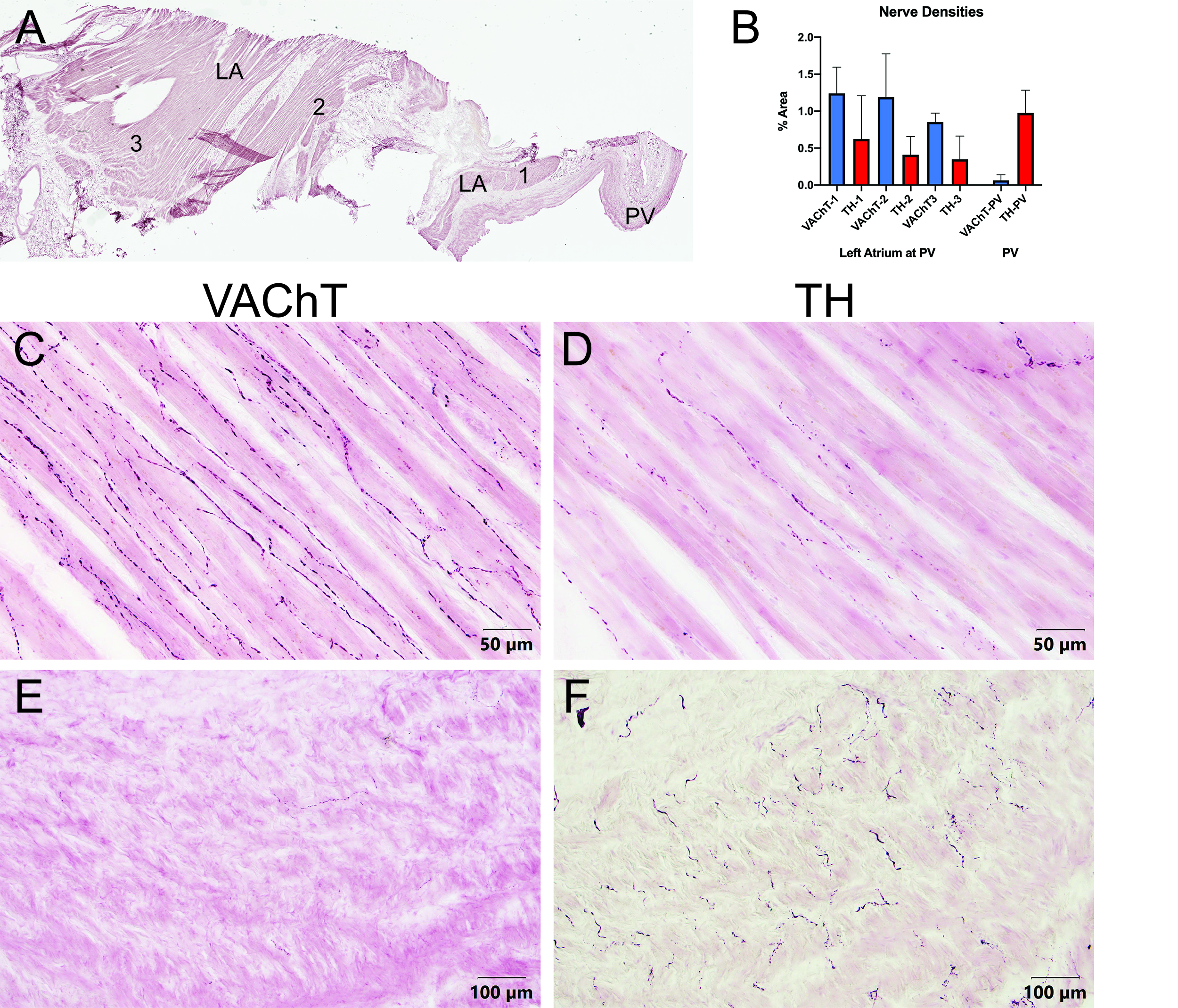

Supplement: Supplementary file 2 — Figure S2. Comparison of cholinergic and noradrenergic innervation in left atrial (LA) muscle near a pulmonary vein. (a) Slide scan showing a full section containing LA and a portion of a pulmonary vein (PV). Number 1–3 indicated regions of LA where nerve fiber densities were measured. (b) Bar graph shows nerve fiber densities in sections from a single donor. Values are means ± SDs for at least nine images per data point. Note that VAChT+ cholinergic nerve fibers are more abundant than TH+ noradrenergic nerve fibers in the LA muscle, but TH+ nerve fibers outnumber VAChT+ nerve fibers vastly in the vein. (c, d) Representative images showing VAChT+ and TH+ nerve fibers, respectively, in region 2 of LA. (e, f) Representative images showing VAChT+ and TH+ nerve fibers, respectively, in the PV region indicated in panel (a). [file AR-309-417-s001.tif]

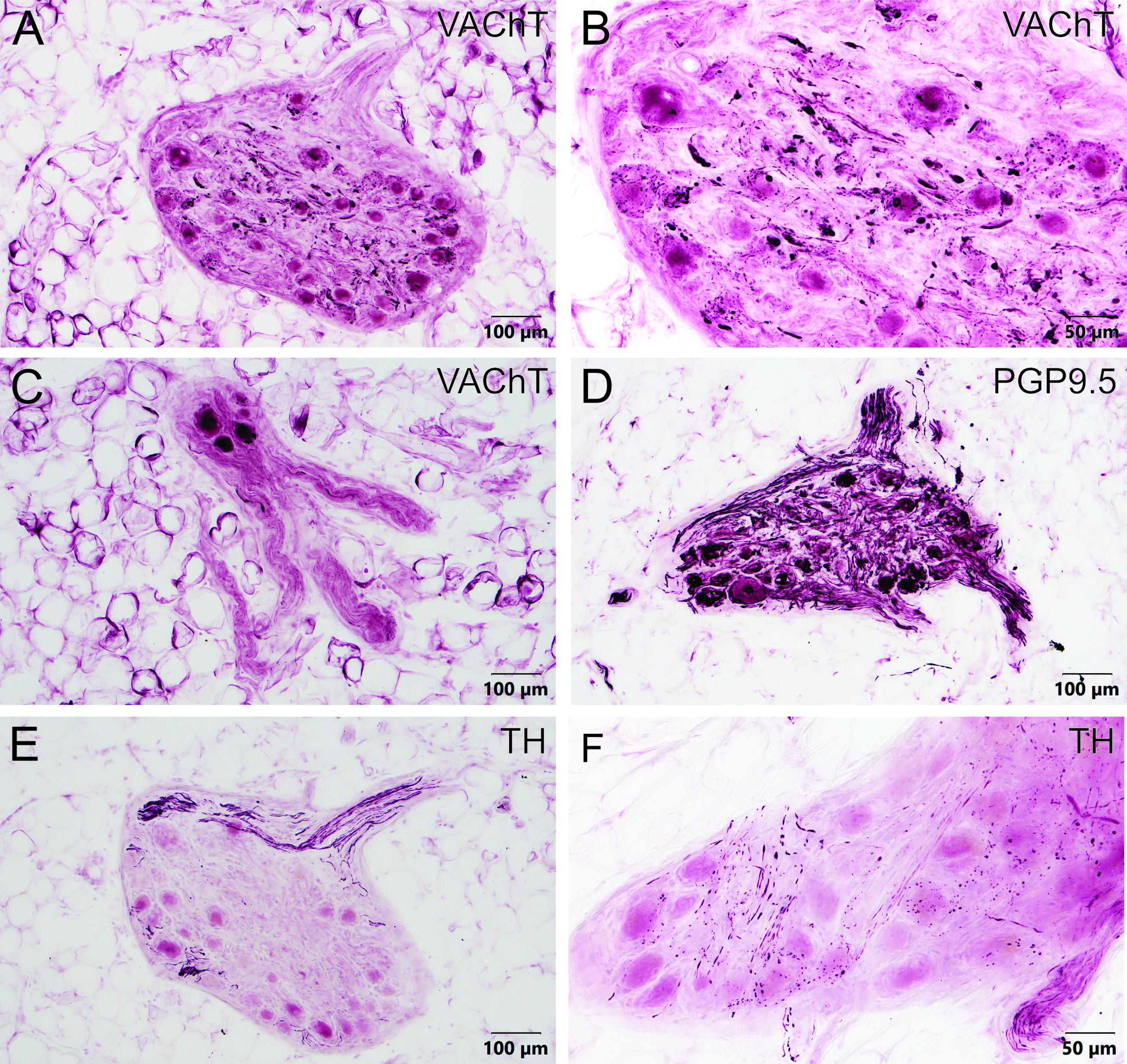

Supplement: Supplementary file 3 — Figure S3. Ganglia located in epicardial fat around the atrium/PV sample shown in Figure S1a–c: Staining for the cholinergic marker VAChT shows cholinergic cell bodies surrounded by cholinergic varicosities and nerve fibers. (a, b) Low and higher magnification images of the same ganglion. Note variable staining intensity of cell bodies. (c) Small ganglion embedded in nerve bundle at branching point. (d) Ganglion stained for the panneuronal marker PGP9.5. Note prominent staining of cell bodies and axons. Varicosities are not evident. (e, f) Two ganglia in sections stained for TH. Cell bodies exhibit mainly background staining but strong staining of nerve fibers is present. (e) TH+ nerves associated with this ganglion occur mainly in a bundle at the edge and few penetrate the ganglion. (f) Higher magnification of TH staining in this ganglion shows that varicose nerve fibers innervate several of the cell bodies, which are presumed to be cholinergic. [file AR-309-417-s013.tif]

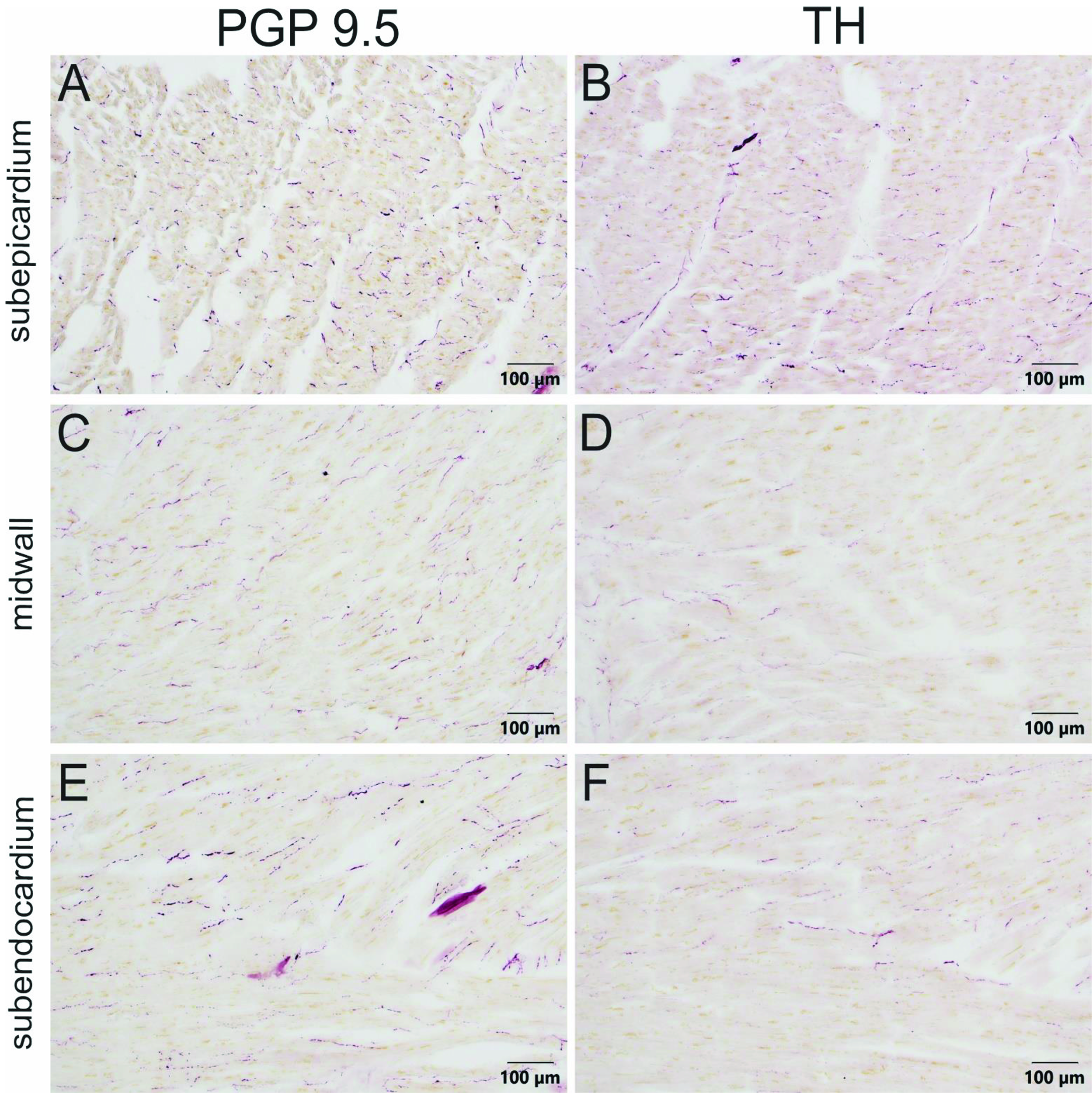

Supplement: Supplementary file 4 — Figure S4. Transmural differences in staining for TH and PGP9.5 in the lateral mid‐LV free wall of control heart from D1. Note decreased staining for TH but not PGP9.5 at the midwall level. [file AR-309-417-s015.tif]

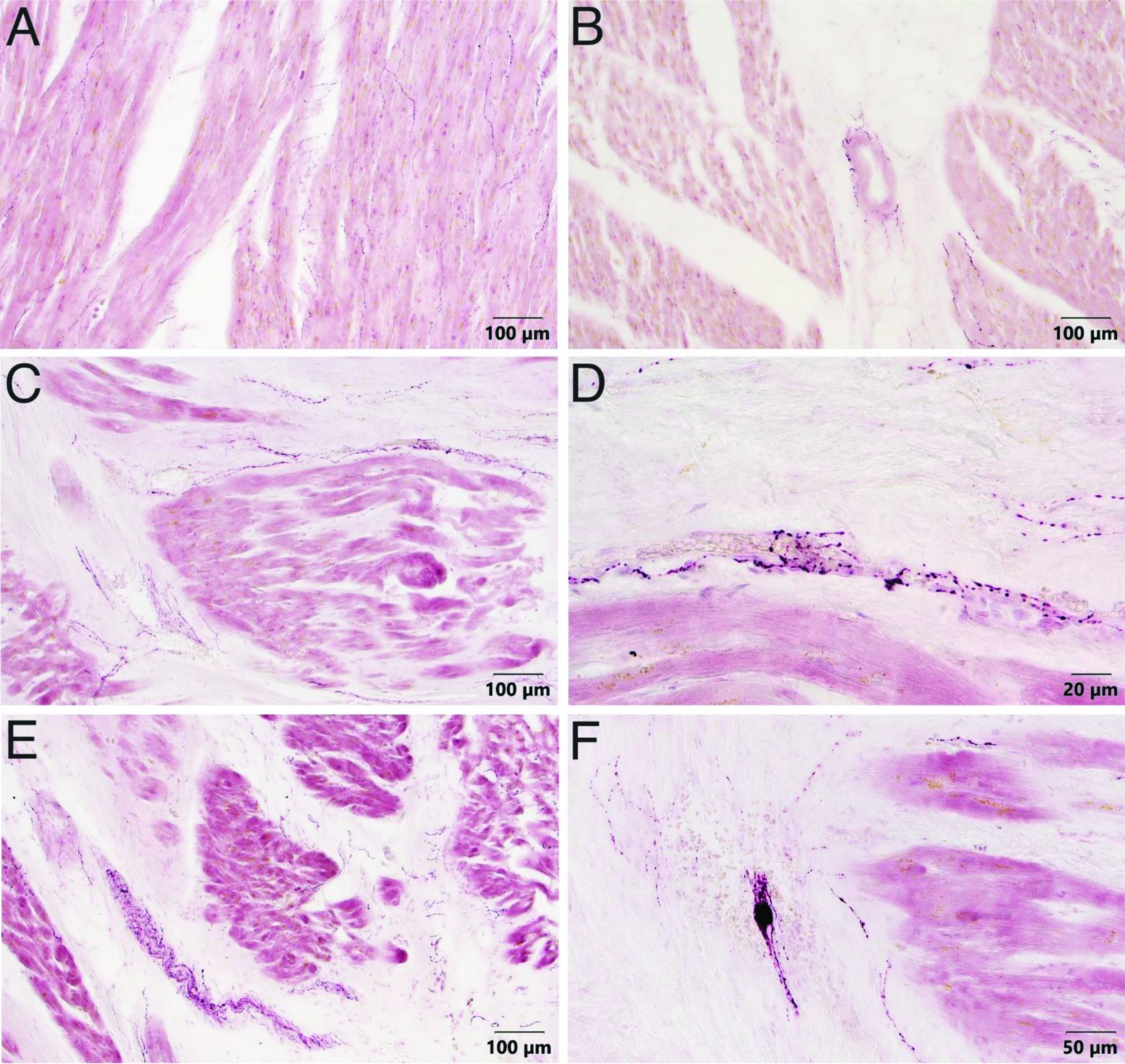

Supplement: Supplementary file 5 — Figure S5. A low density of VAChT+ cholinergic nerves occurred at many regions of LV apex in control donor heart D9. (a) Subepicardium. (b) VAChT+ nerves surround an artery and innervate muscle at a midwall region. (c) VAChT+ nerve fibers traverse connective tissue and surround a micro vessel above muscle at top right. (d) Higher magnification image of same field as panel (c) showing cholinergic innervation of micro vessel. (e) Nerve bundle at the lower left contains several VAChT+ nerve fibers. (f) Single cholinergic neuron observed in the heart was the only neuron associated ventricular tissue among all the hearts evaluated. [file AR-309-417-s019.tif]

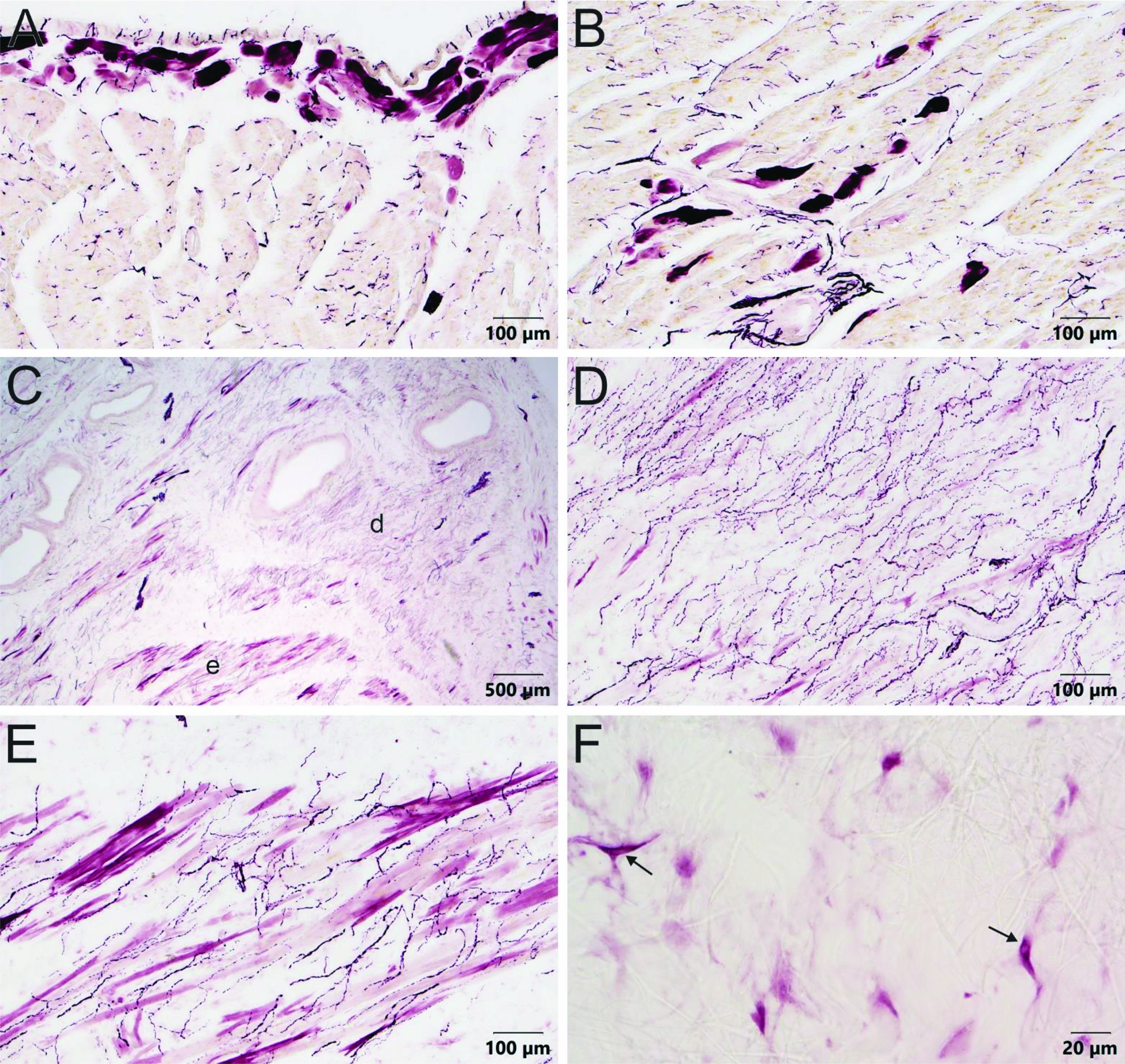

Supplement: Supplementary file 6 — Figure S6. Expression of PGP9.5 by cardiomyocytes occurred at specific sites in control hearts. (a) Purkinje cells and nerve fibers in the mid‐LV septum had intense staining for PGP9.5 that was noted in sections and tissue cleared specimens as the endocardial aspect of the mid‐left ventricular anterior wall. (b) Several myocytes at the LV apex stained strongly for PGP9.5, but such staining was not detected at other ventricular sites beyond the conducting system. (c) Low magnification image of the SA node (d) and surrounding right atrium (e) showing staining pattern for PGP9.5. (d) Higher magnification of the SA node region (d in panel (c)) where PGP9.5 is localized only to nerve fibers. (e) Higher magnification of the surrounding right atrium (e in panel (c)) where PGP9.5 is localized to cardiomyocytes and nerves. (f) PGP9.5 staining of fibroblasts (arrows) in subepicardium near the SAN. All these images were from control donor D1. [file AR-309-417-s006.tif]

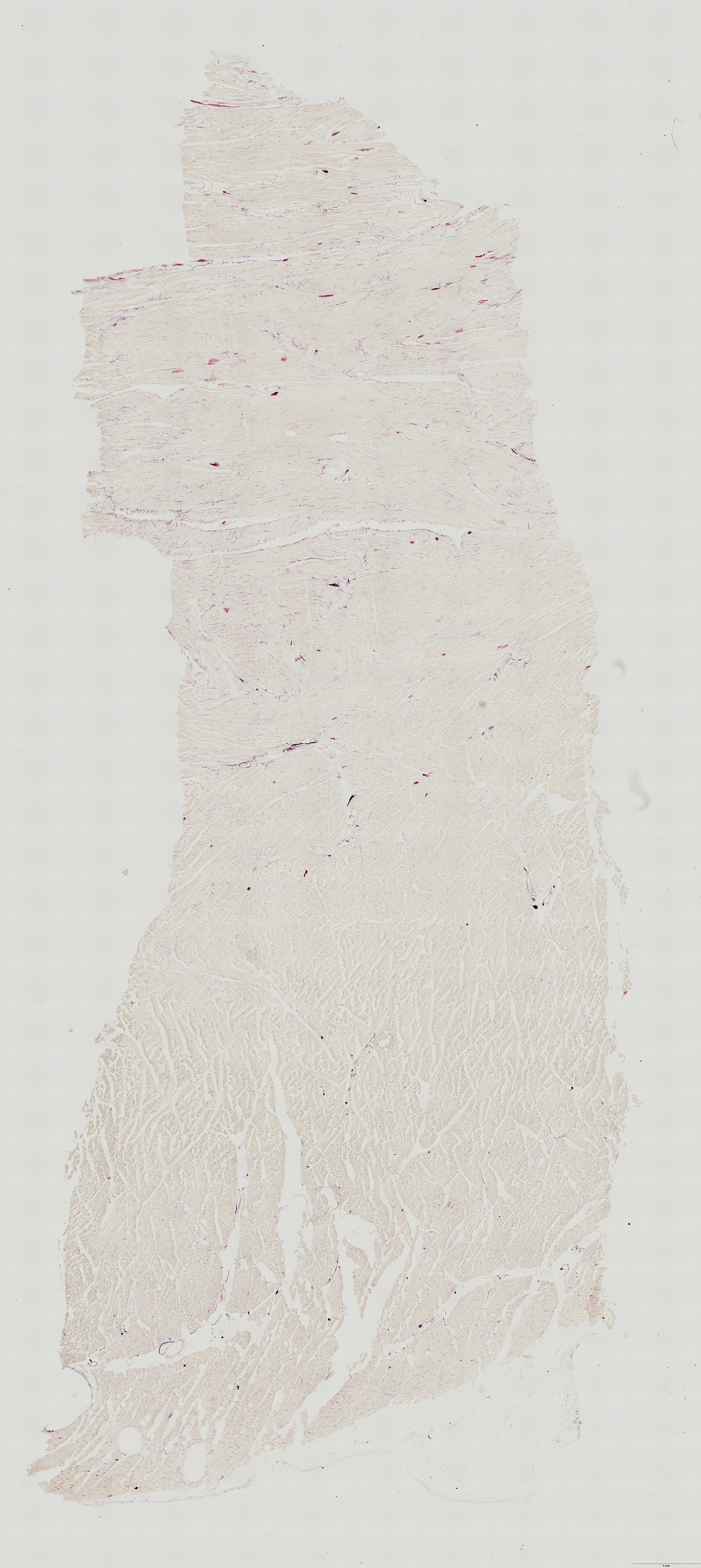

Supplement: Supplementary file 7 — Figure S7. Montage image showing PGP9.5 stain of a transmural section of anterior mid‐LV from donor D1. Montage was created by stitching multiple 10× images using an Olympus BX41 microscope equipped with an Olympus DP74 digital camera and cellSens Dimension software. [file AR-309-417-s012.jpg]

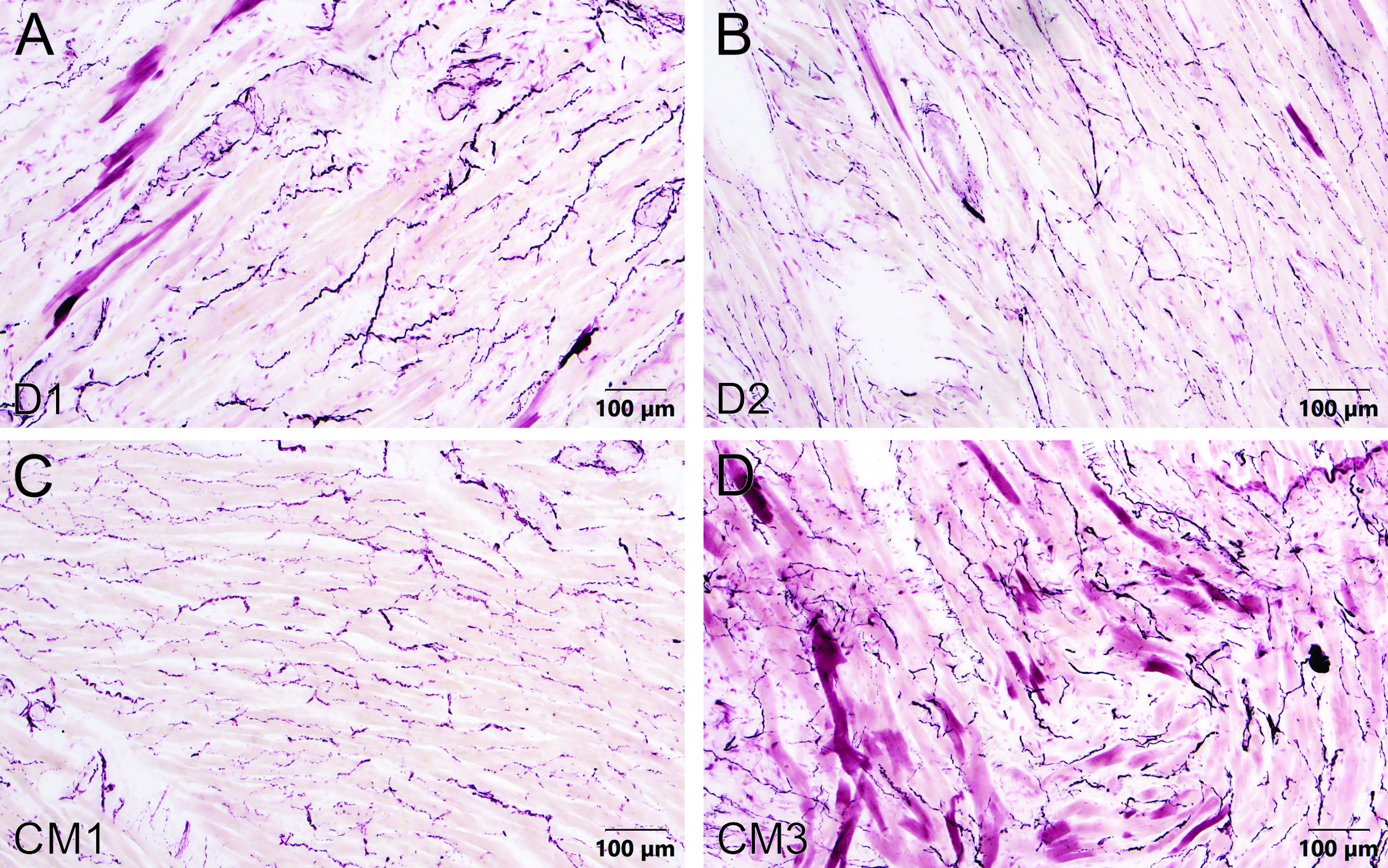

Supplement: Supplementary file 8 — Figure S8. Variable staining of PGP9.5+ myocytes were observed in the right atrium of normal and cardiomyopathy hearts. (a) Image showing typical myocyte staining pattern within the RA of D1. (b) Limited quantities of PGP9.5+ myocytes were observed in D2. (c) Representative image showing lack of PGP9.5+ myocytes in donor CM1. (d) Extensive quantities of PGP9.5+ myocytes were observed in the RA in donor CM5. [file AR-309-417-s007.tif]

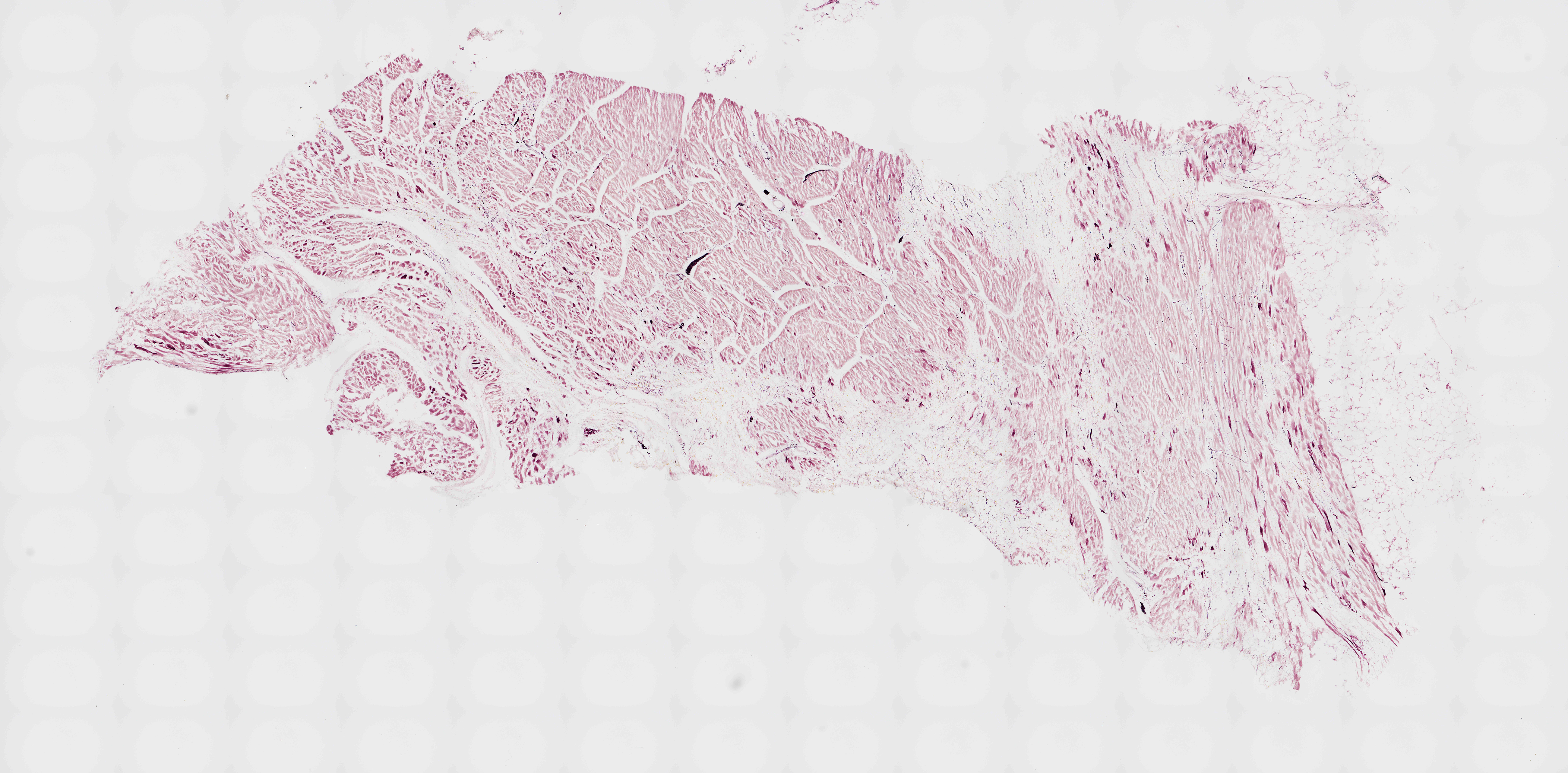

Supplement: Supplementary file 9 — Figure S9. Montage image showing PGP9.5 stain of a large section of lateral mid‐LV from donor CM2 (NICM). [file AR-309-417-s018.jpg]

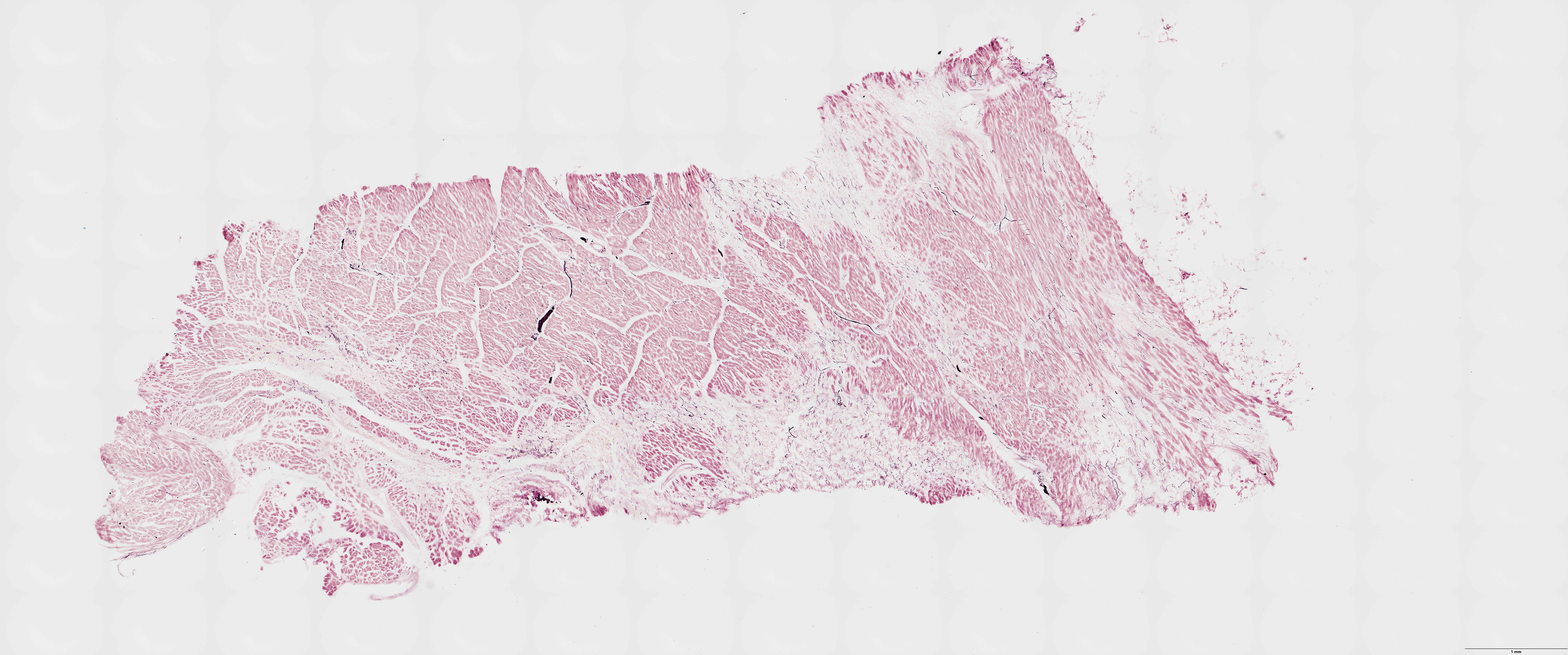

Supplement: Supplementary file 10 — Figure S10. Montage image showing TH stain of a transmural section of lateral mid‐LV from donor CM2 (NICM). [file AR-309-417-s014.jpg]

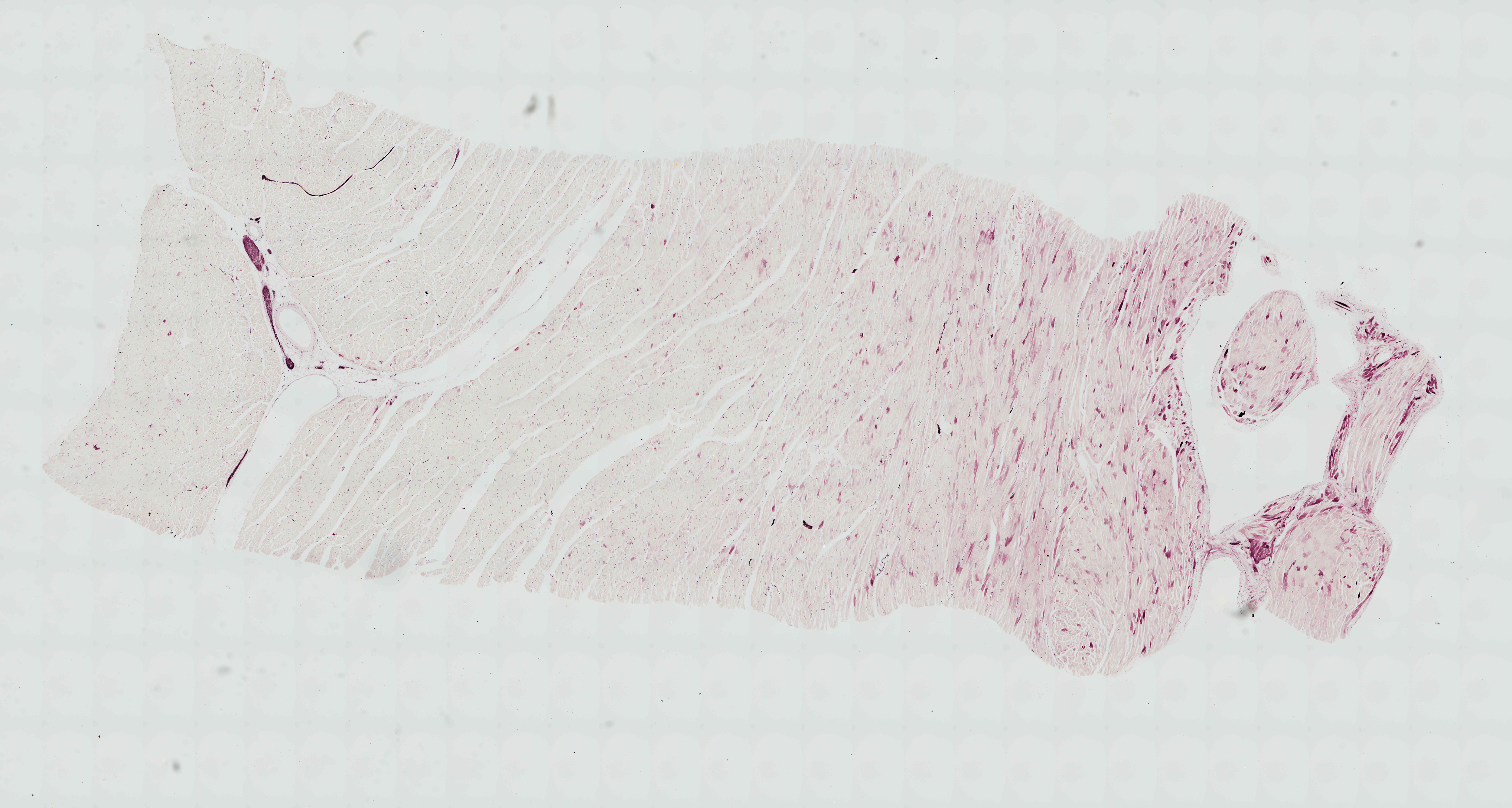

Supplement: Supplementary file 11 — Figure S11. Montage image showing PGP9.5 stain of a transmural section of anterior mid‐LV from donor CM1 (NICM). Montage was created by stitching multiple 10× images using an Olympus BX41 microscope equipped with an Olympus DP74 digital camera and cellSens Dimension software. [file AR-309-417-s004.jpg]

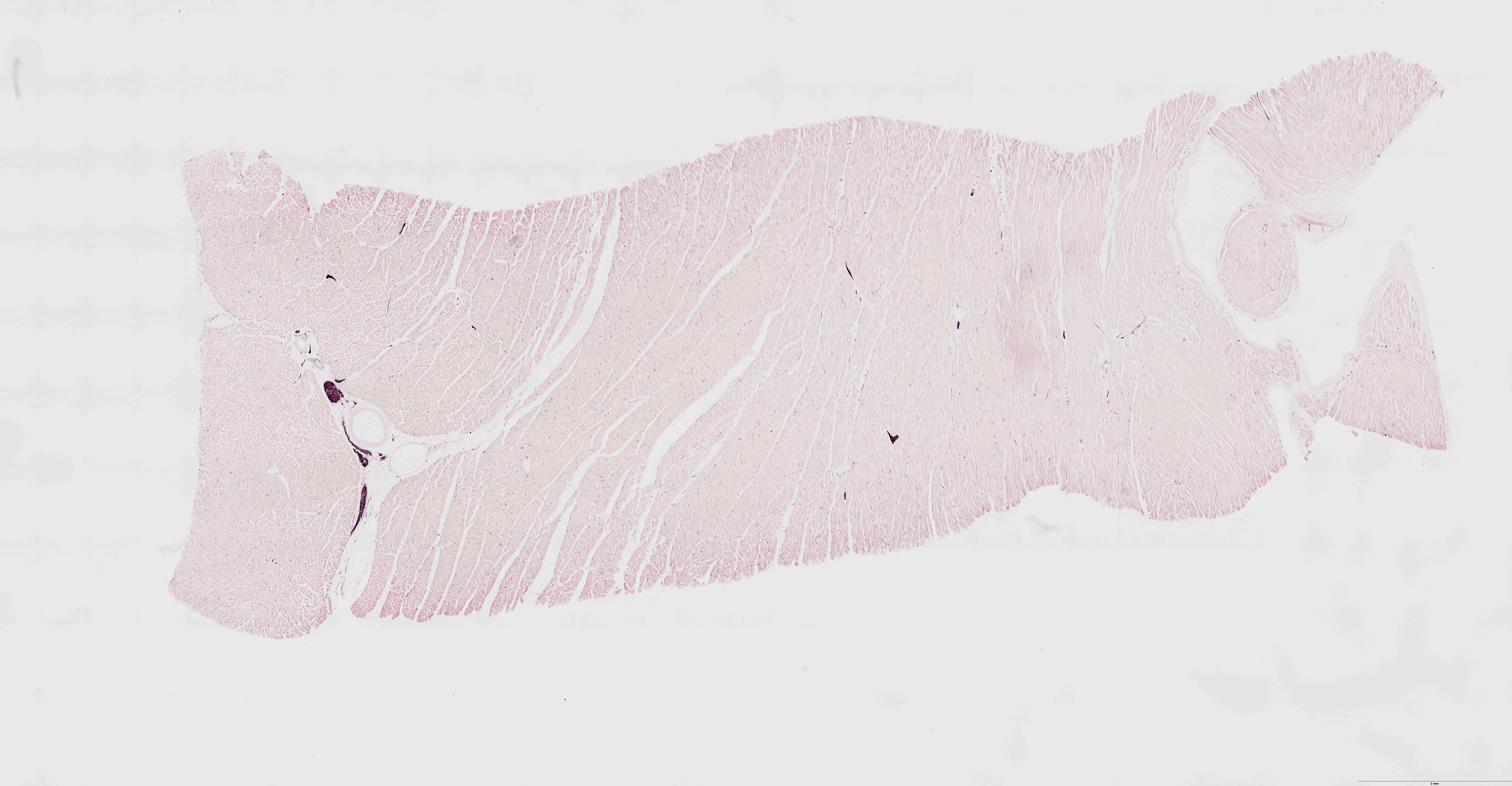

Supplement: Supplementary file 12 — Figure S12. Montage image showing TH stain of a transmural section of anterior mid‐LV from donor CM1 (NICM). [file AR-309-417-s008.jpg]

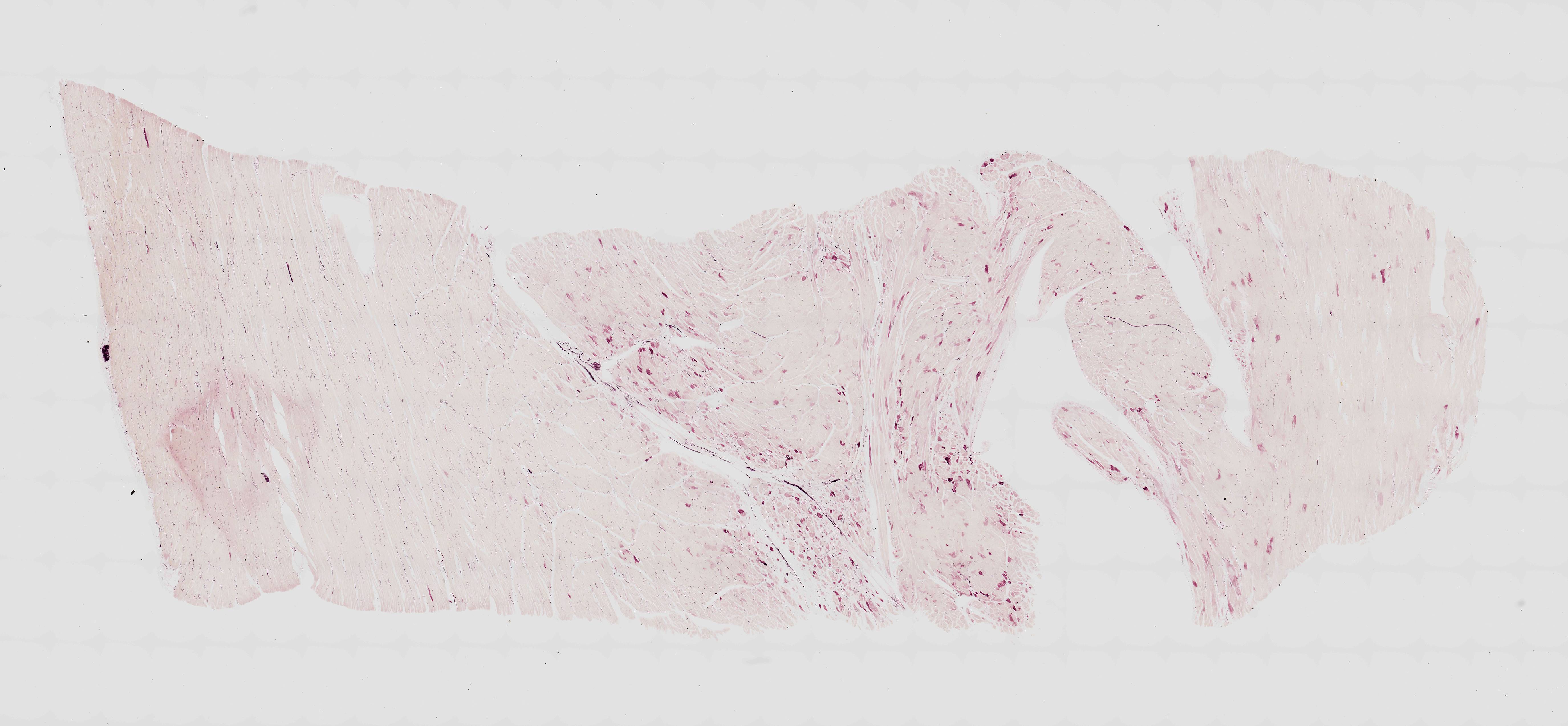

Supplement: Supplementary file 13 — Figure S13. Montage image showing PGP9.5 stain of a transmural section of lateral mid‐LV from donor CM1 (NICM). [file AR-309-417-s009.jpg]

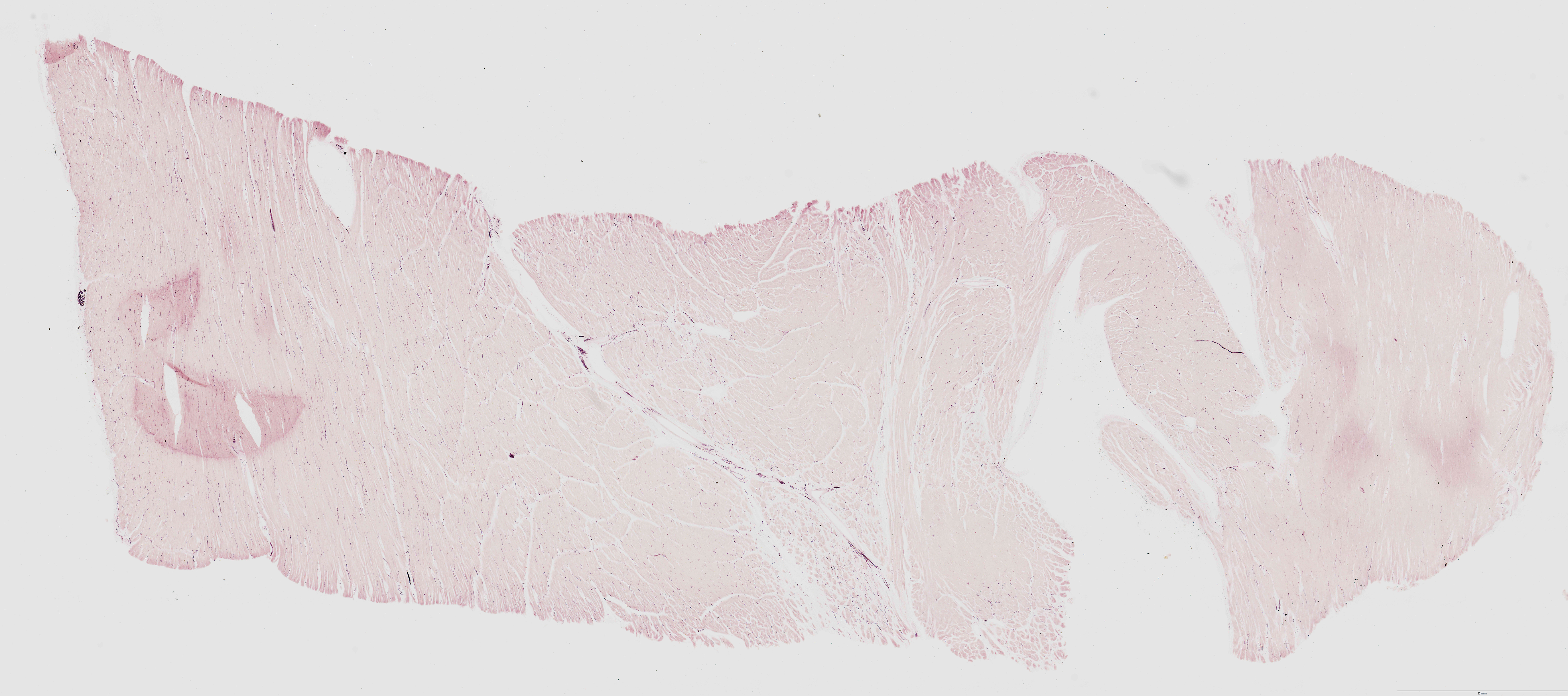

Supplement: Supplementary file 14 — Figure S14. Montage image showing TH stain of a transmural section of lateral mid‐LV from donor CM1 (NICM). TH+ nerves were observed across the entirety of the tissue, from subendocardium to subepicardium. Note the heterogeneity of TH+ nerve density across the tissue wherein large areas of tissue have little‐to‐no innervation while others are densely innervated. Montage was created by stitching multiple 10× images using an Olympus BX41 microscope equipped with an Olympus DP74 digital camera and cellSens Dimension software. [file AR-309-417-s017.jpg]

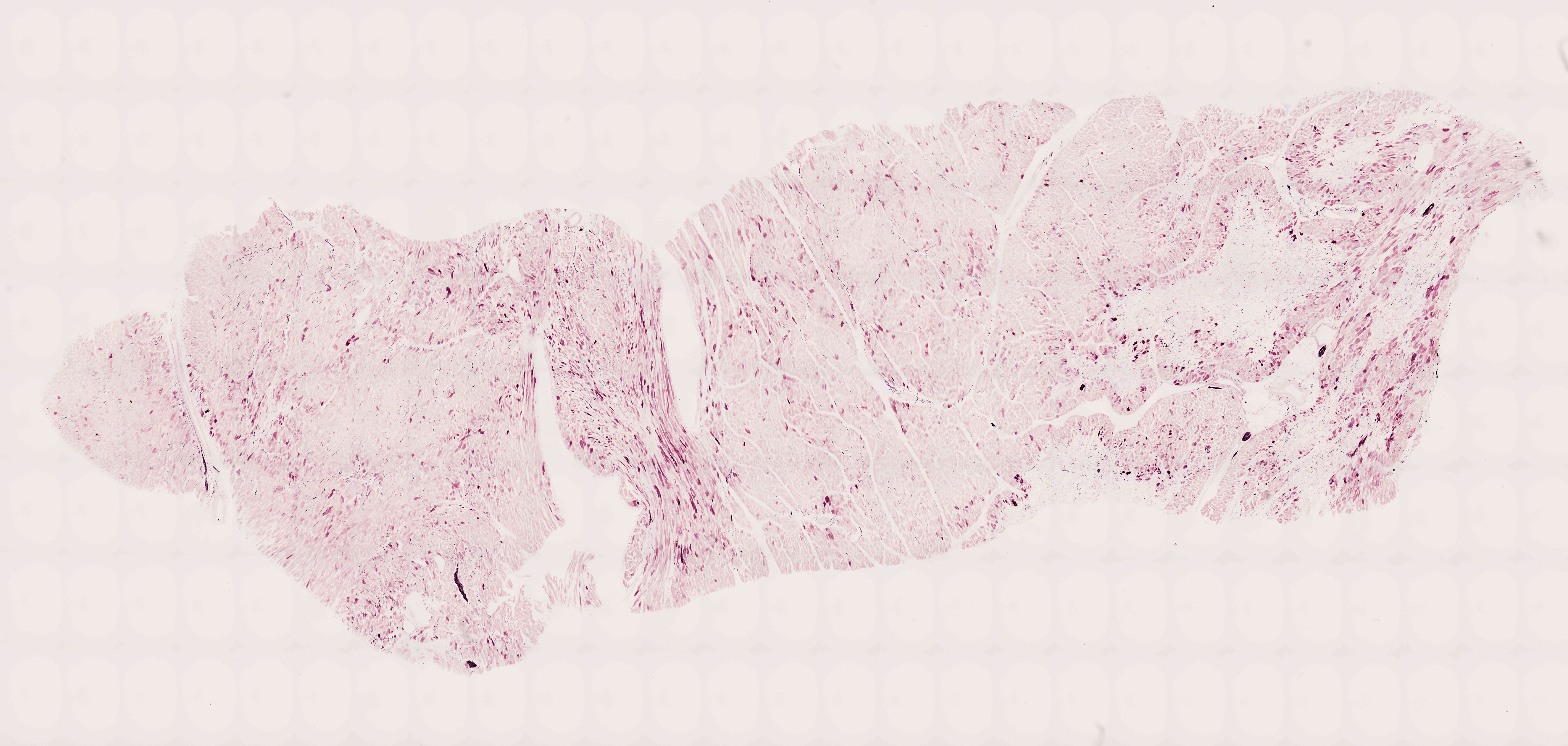

Supplement: Supplementary file 15 — Figure S15. Montage image showing PGP9.5 stain of a transmural section of posterior mid‐LV from donor CM2 (NICM). [file AR-309-417-s010.jpg]

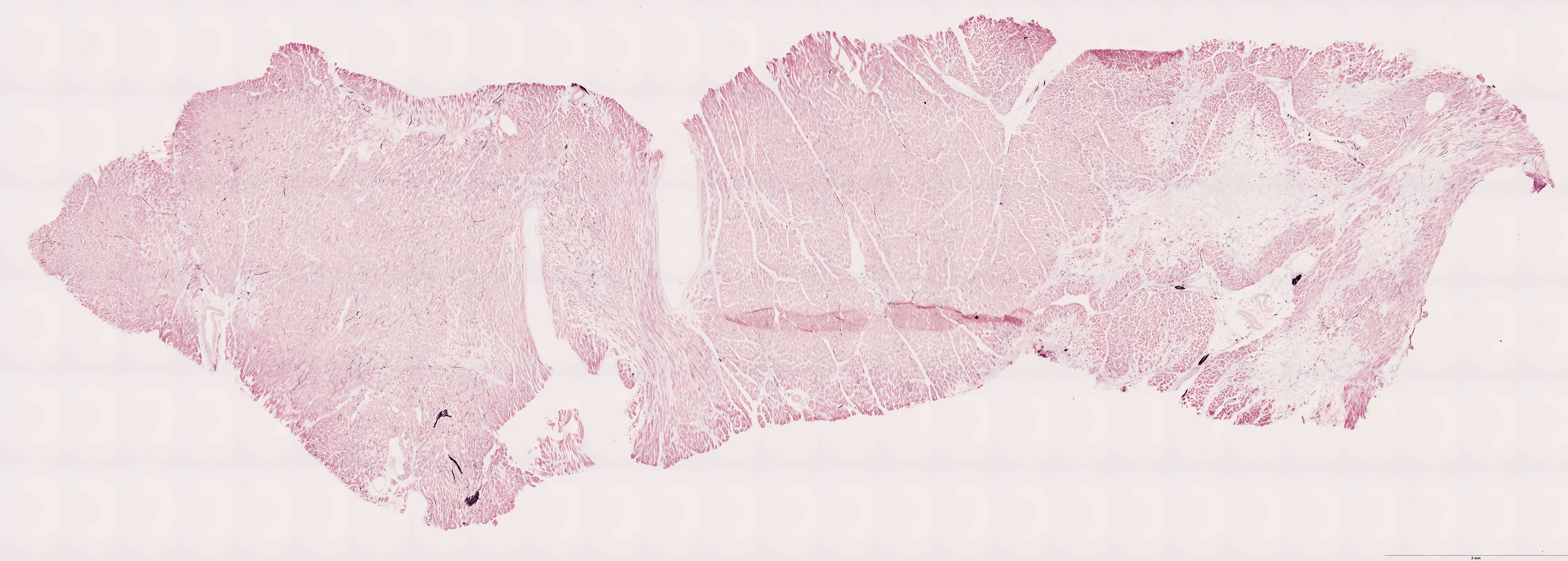

Supplement: Supplementary file 16 — Figure S16. Montage image showing TH stain of a transmural section of posterior mid‐LV from donor CM2 (NICM). [file AR-309-417-s005.jpg]

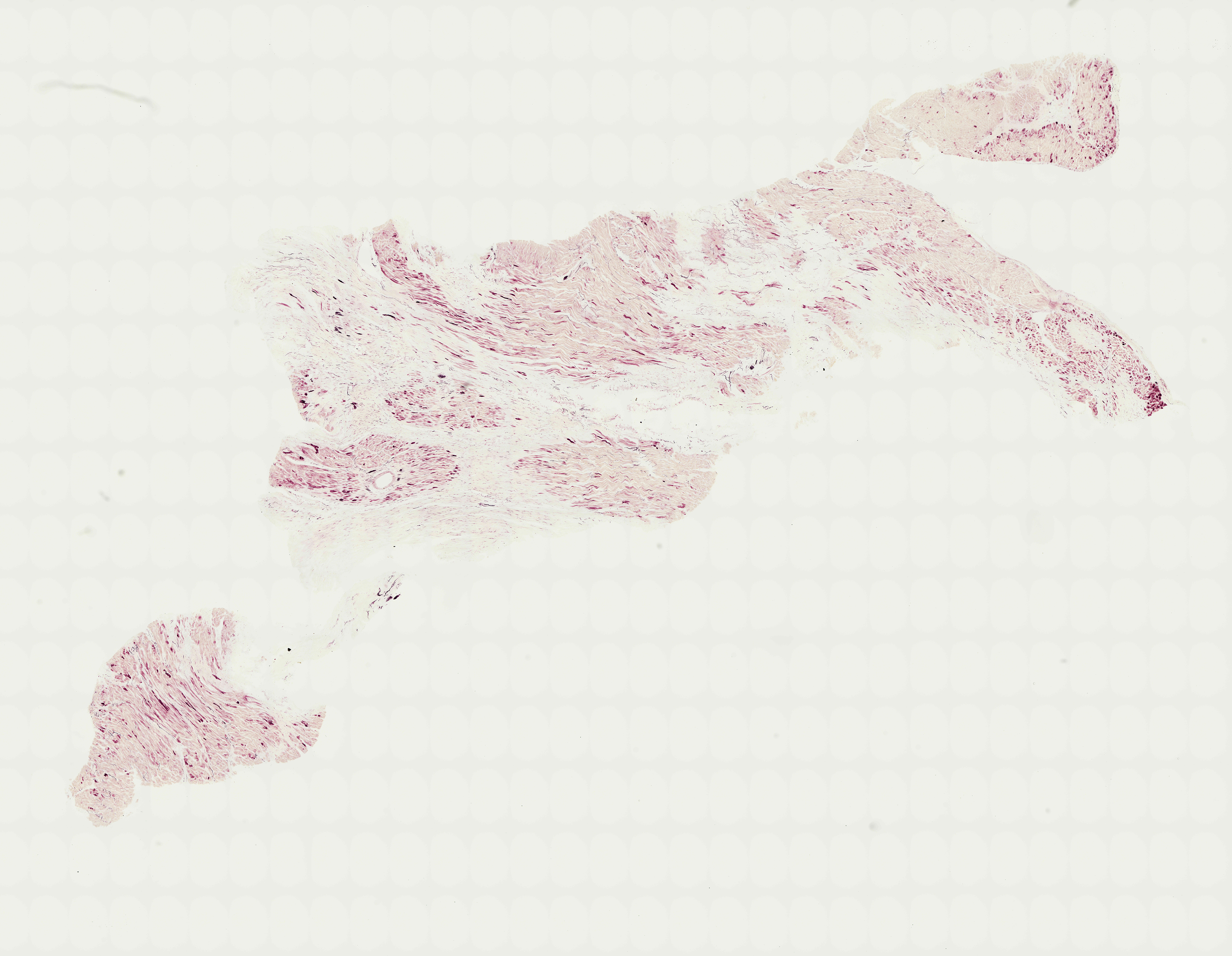

Supplement: Supplementary file 17 — Figure S17. Montage image showing PGP9.5 stain of a transmural section of mid septum from donor CM5 (ICM). [file AR-309-417-s016.jpg]

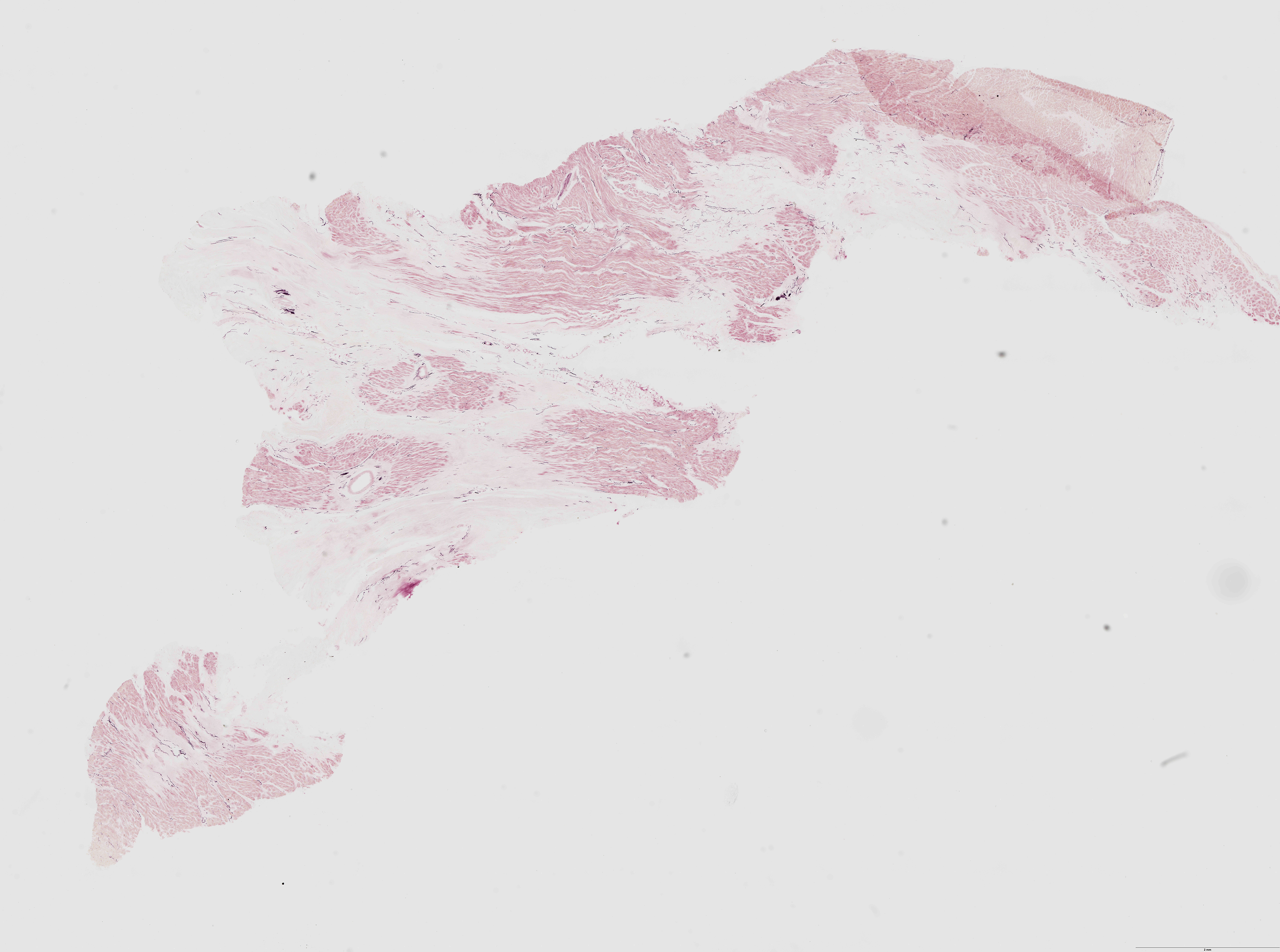

Supplement: Supplementary file 18 — Figure S18. Montage image showing TH stain of a transmural section of mid septum from donor CM5 (ICM). [file AR-309-417-s011.jpg]

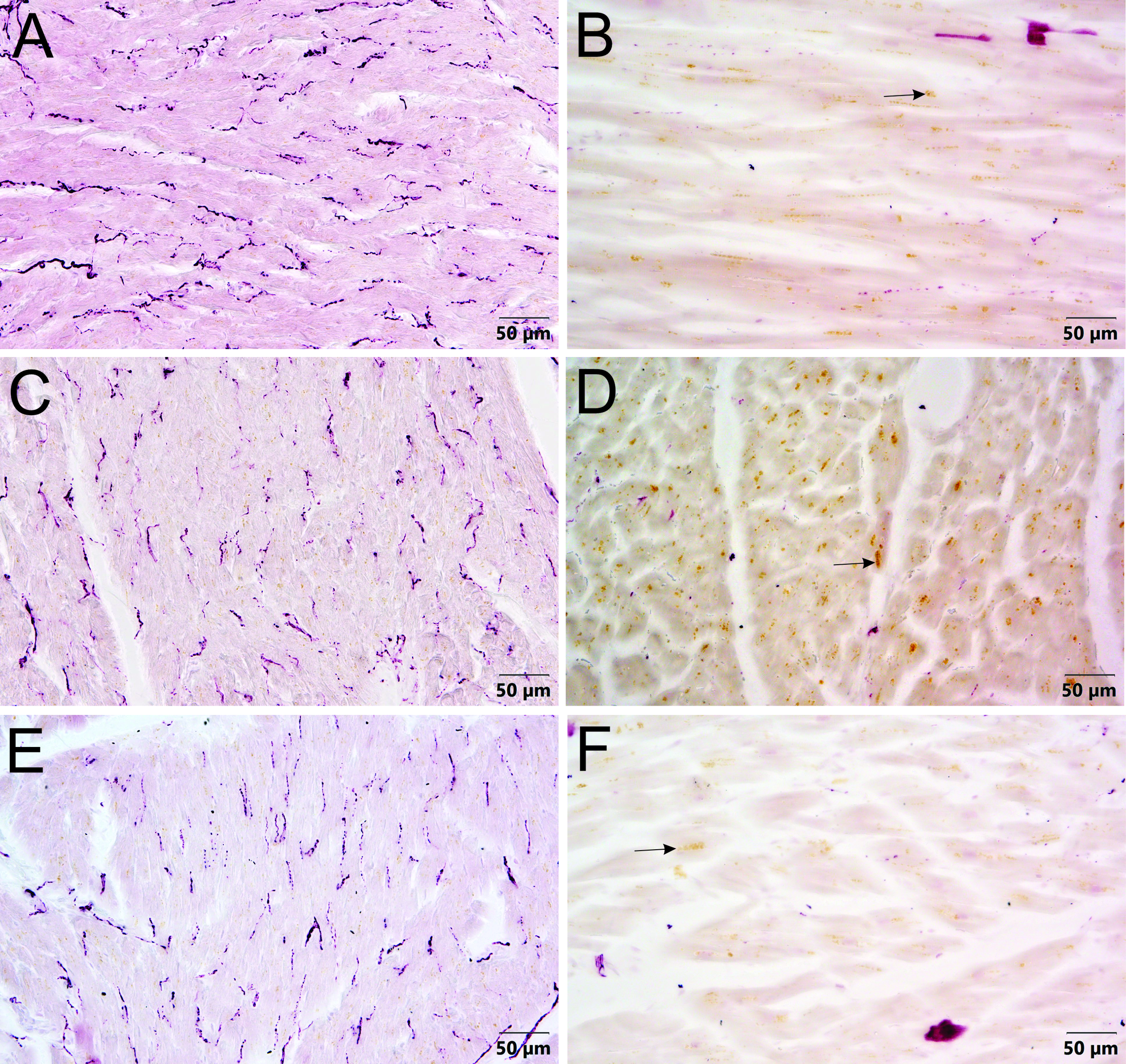

Supplement: Supplementary file 19 — Figure S19. Lipofuscin deposits were found in varying quantities in the mid‐LV of two donors (D9 and D2). (a) Image of subepicardium from D9 showing limited amounts of lipofuscin (orange dots) within cardiomyocytes. (b) Image of subepicardium showing lipofuscin deposits (arrow) in D2. (c) Image of midwall PGP9.5 stain showing lipofuscin deposits in D9. Note the increased amount of lipofuscin staining in panel (c) compared to panels (a) or (e). (d) Lipofuscin deposits (arrow) within the midwall of D2. (e) PGP9.5 staining of subendocardium in D9 shows limited amount of intramuscular lipofuscin. (f) PGP9.5 staining shows large lipofuscin deposits (arrow) in subendocardium in D2. [file AR-309-417-s002.tif]
